# Identification of immune-associated genes with altered expression in the spleen of mice enriched with probiotic *Lactobacillus* species using RNA-seq profiling

**DOI:** 10.5713/ab.24.0280

**Published:** 2024-08-26

**Authors:** Anh Duc Truong, Ha Thi Thanh Tran, Nhu Thi Chu, Lanh Phan, Hoai Thi Phan, Thu Huong Dang, Hoang Vu Dang, La Anh Nguyen

**Affiliations:** 1 Department of Biochemistry and Immunology, National Institute of Veterinary Research, Dong Da, Hanoi 100000, Vietnam; 2 Department of Microbial Biotechnology, Food Industries Research Institute, Thanh Xuan Distr., Hanoi 100000, Vietnam

**Keywords:** Differentially Expressed Gene, Immune Response, *Lactobacillus* spp., Probiotic, Signaling Pathway

## Abstract

**Objective:**

Probiotics are living microorganisms that can provide health benefits when consumed. Here, we investigated the effects of probiotics on gene expression in the spleen of mice using RNA-sequencing analysis between negative control and probiotic groups (including 4 *Lactobacillus* strains: *Lactobacillus fermentum*, *L. casei*, *L. plantarum*, and *L. brevis*).

**Methods:**

Mice exposed with probiotic in 4 weeks by intragastric administration. Then, spleen tissues of the control and probiotics groups were collected on days 14 and 28 for RNA sequencing.

**Results:**

In total, 665, 186, and 81 differentially expressed genes (DEGs) were significantly expressed on day 14 vs control, day 28 vs control groups, and probiotics day 28 vs day 14 groups, respectively. On the other hand, 12 toll-like receptor genes underwent additional validation through quantitative real-time polymerase chain reaction (qRT-PCR), affirming the increased alignment between qRT-PCR and RNA-Seq findings. In addition, the Kyoto encyclopedia of genes and genomes and gene ontology analyses revealed that the DEGs were predominantly enriched in defense responses to pathogens, including inflammatory bowel diseases, malaria, leukaemia virus 1, and herpes virus, as well as immune processes related to immune response and signal transduction. This study represents the first investigation into mice’s gene expression in the spleen exposed to probiotics using *Lactobacillus* spp. isolated from a field strain in Vietnam.

**Conclusion:**

Our results provide valuable insights into the impacts and functions of probiotics on mammalian development, offering crucial information for the potential therapeutic use of probiotics in defending against pathogens in Vietnam. The findings from this study highlight the potential of probiotics in modulating gene expression in the spleen, which may have implications for immune function and overall health in mice.

## INTRODUCTION

Probiotics have demonstrated their capacity to impact both innate and adaptive immune responses through direct interactions with epithelial and immune cells or by modulating the composition and functionality of the gut microbiota [1]. Their protective effects are mediated by a multitude of mechanisms, encompassing both immune-related and non-immune pathways [2]. These mechanisms encompass a range of actions, such as direct antimicrobial activity against pathogens [3], augmentation of phagocytosis [3], modulation of cytokine production across various cell populations [4], and enhancement of immunoglobulin production [5]. Among the fundamental mechanisms that probiotics employ to guard against gastroenteric infections, modulation of pro-inflammatory factors like interferon γ (IFN-γ) and tumor necrosis factor α (TNF-α) and the anti-inflammatory cytokine interleukin (IL)-10 stands out [5]. However, the exact pathways and cell types orchestrating these mechanisms remain to be fully elucidated [6]. Importantly, it is evident that distinct microorganisms wield varying effects on their host, and probiotic attributes are contingent upon the specific strain and host context. In this regard, it’s essential to recognize that findings related to one probiotic strain cannot be universally extrapolated to another, nor can the effects against a specific pathogen be assumed to apply uniformly to other pathogens [5].

Lactic acid-producing bacteria (LAB) are regularly present in mammals’ and avian species’ small and large intestines [7]. Notably, *Lactobacillus*, a Gram-positive facultative anaerobic bacteria genus, dominates the LAB group [8]. LAB, commonly harnessed as probiotics, has garnered extensive research attention, particularly concerning the host’s reaction to these microbes [9]. Host responses manifest after exposure to live LAB, diverse structural components of LAB, and the byproducts synthesized by these bacteria, both *in vivo* and *in vitro* contexts [10,11]. However, the precise response hinges on the species and even the specific strain of bacteria employed. In the context of mammals, probiotic LAB has been observed to bolster intestinal mucosal immunity [12], amplify the serum antibody response [13], and induce the immune-related genes associated with immune responses [14]. The immunomodulatory effects of probiotic bacteria could be attributed to their capacity to stimulate cytokine production, thereby orchestrating regulation within innate and adaptive immune reactions. In the mammalian context, the gastrointestinal microbiota’s capability to influence the equilibrium of distinct T-helper (Th) cell subsets (Th1, Th2, Th3, and T regulatory [Treg]) and their correlated cytokines is widely acknowledged [11,15,16]. Certain species and strains of LAB have been shown to induce cytokines that support Th1 effector functions, such as IL-12 [15,16], while others prompt the generation of immunoregulatory cytokines, including IL-10 and transforming growth factor β (TGF-β) [15]. Within the mammal’s realm, *Lactobacillus fermentum*, *L. casei*, *L. plantarum*, *L. brevis* constitute native inhabitants of the mice intestine [17]. Selected strains from these four bacterial species have positively impacted the host’s immune systems.

In Vietnam, the research on global gene expression in the spleen of mice following *Lactobacillus* spp. supplementation isolated in Vietnam still needs to be done. In our study, mice were intragastrically administered *Lactobacillus* spp. at 14 and 28 days, their spleens were harvested for total RNA extraction. Using RNA-seq, we explored a differential gene library and identified immune-related genes. We also employed quantitative real-time polymerase chain reaction (qRT-PCR) to validate these genes, seeking to pinpoint essential immunity-regulating genes influenced by *Lactobacillus* spp., bolstering its broader use in mice production.

## MATERIALS AND METHODS

### Animal ethics

All animal experiments adhered to the guidelines for the care and utilization of laboratory animals and received approval from the Ministry of Agriculture and Rural Development, Vietnam (TCVN 8402:2010).

### Laboratory animals and probiotic strains

Eight-week-old, specific pathogen-free Swiss mice weighing 22 to 26 g were obtained from a local company in Hanoi, which has been validated and confirmed to be disease-free by the Vietnam National Institute of Veterinary Research (NIVR). Our research groups successfully isolated Vietnam’s *L. fermentum*, *L. casei*, *L. plantarum*, and *L. brevis* strains. Specifically, Lactobacillus strains, including L. plantarum NCDC3 and L. brevis NCTH24 (originated from fermented pork meat), *L. fermentum* SBV2 (originated from fermented milk), and *L. casei* PK2 (originated from the faeces of breastfed infants), were sourced from FIRI’s culture collection. These strains demonstrated desirable probiotic characteristics, including tolerance to low pH and resistance to bile salts, persistence in the gastrointestinal tract, adhesion to simulated intestinal mucus, and inhibition of the growth of enteric pathogens. All *Lactobacillus* strains were preserved in 40% glycerol stocks at −80°C and reactivated by culturing in de Man, Rogosa Sharpe (MRS) broth at 37°C for 24 hours [18]. The probiotic mixture consisted of four strains of *L. fermentum*, *L. casei*, *L. plantarum*, and *L. brevis* (1×10^9^ CFU/g; 8%).

### Experimental design

A total of one hundred diseases-free Swiss mice, aged eight weeks (weighting 22 to 26 g), were purchased from a local company in Hanoi capital, Vietnam. The animals were separated into control and probiotic complex groups, with equal mean body weights (Fifty mice per group, 10 mice per case). A probiotic complex was administered with a mix of *Lactobacillus* spp. (1.0×10^9^ CFU/mL, 0.2 mL) by intragastric administration for 28 days, whereas the control group was given phosphate-buffered saline (0.2 mL). On days 14 and 28, three mice in each group were euthanized, and their spleens were sampled and immediately taken into liquid nitrogen and stored at −80°C for analysis. Commercial feed (21% crude protein, 5% to 7% crude lipid, 5% to 6% crude fiber, and 6% to 8% mineral; Food Industries Research Institute, Hanoi, Vietnam) was purchased as the basal component of the diet. During the experimental period, 12 h light per day was provided, and water and feed were offered *ad libitum* throughout the experiment. The initial room temperature was set at 25°C±2°C and humidity at 55%±5%. The body weight was individually evaluated by weight measured weekly.

### RNA extraction, library construction, and sequencing

Total RNA extraction from spleen samples of both control and treated groups was conducted using TRizol reagent (Invitrogen, Carlsbad, CA, USA) [19]. Subsequently, RNA concentrations were determined using a NanoDrop spectrophotometer (NanoDrop Technologies, Waltham, MA, USA), aiming for a 260/280 nm ratio within the range of 1.7 to 2.0. The integrity of the total RNA was assessed using Agilent 2100 (Agilent Technologies, Inc., Santa Clara, CA, USA) and Tecan F2000 (Tecan Group Ltd., Männedorf, Switzerland) devices, with only samples having an RNA integrity number >7.0 and exhibiting high-quality RNA (28S/18S >1) being utilized for subsequent experiments. Subsequently, 1 μg of total RNA was used for library preparation. Poly(A) mRNA isolation was carried out using Oligo(dT) beads, followed by mRNA fragmentation using divalent cations and high temperatures. Priming utilized Random Primers. First-strand cDNA and second-strand cDNA synthesis was conducted. The resulting double-stranded cDNA was end repaired and underwent dA-tailing in a single reaction, followed by T-A ligation to add adaptors to both ends. Size selection of Adaptor-ligated DNA was performed using DNA Clean Beads. Each sample was PCR amplified using P5 and P7 primers, and the PCR products were validated. Subsequently, libraries with different indexes were multiplexed and loaded onto an Illumina HiSeq instrument for sequencing using a 2×150 paired-end configuration following the manufacturer’s instructions.

### Quality validation and read mapping

The quality evaluation of the raw sequence data was carried out using FastQC version 0.11.5, available at http://www.bioinformatics.babraham.ac.uk/projects/fastqc/. Following this, sequence adapters and low-quality bases were removed from the raw reads using Cutadapt version 1.9.1. The parameters set for Cutadapt included a phred quality score cutoff of 20, an error rate of 0.1, an adapter overlap minimum of 1 base pair, a minimum sequence length of 75 bases, and a maximum proportion of undetermined bases (N) of 0.1. This process ensured the production of high-quality, clean data. The resulting high-quality reads were then aligned to the mouse (Mus musculus) genome assembly GRCm39, accessed from the UCSC database at https://genome.ucsc.edu, using Hisat2 version 2.2.1. Sequencing libraries were constructed utilizing the Illumina strand-specific library preparation kit and aligned against the GRCm39 reference genome. Gene expression was quantified in terms of fragments per kilobase of transcript per million mapped reads (FPKM), following the method described by Mortazavi et al [20]. The DEGs were identified using the DESeq2 package from Bioconductor, which applies a model based on the negative binomial distribution to account for dispersion and logarithmic fold changes, with data-driven prior distributions. An adjusted p-value (Padj) threshold of ≤0.05 was employed to determine significant differences in gene expression.

### Biological function analysis

Differentially expressed genes (DEGs) between the infected and control groups were analysed, and functionally categorised genes were examined for their biological roles using gene ontology (GO) categories, encompassing biological process, cellular component, and molecular function. This analysis was performed using GOSeq (v1.34.1), with a significance threshold of padj ≤0.05. Additionally, the cellular pathways associated with the DEGs were investigated using the Kyoto encyclopedia of genes and genomes (KEGG) pathway-mapping database, tailored to organism-specific parameters (Mus musculus). The significance threshold for pathway analysis was set at padj ≤0.05.

### cDNA synthesis and quantitative real-time polymerase chain reaction

To assess mRNA transcripts, two micrograms of total RNA extracted from the spleen was subjected to DNase I treatment (Sigma, St. Louis, MO, USA), according to the manufacturer’s guidelines, to remove potential genomic DNA contamination. After this, the purified RNA was reverse transcribed using the Maxima First Strand cDNA Synthesis Kit from Thermo Scientific, as per the manufacturer’s protocols. Primer sequences for various cytokines, previously identified by Lin et al [21], were employed to evaluate cytokine transcripts. The qRT-PCR was performed using the 2× Ampigen SYBR Green Master Mix from Enzo Life Sciences, based in Farmingdale, NY, USA, following the product’s instructions on a QuantStudio 5 Real-Time PCR System by Thermo Scientific. The glyceraldehyde 3-phosphate dehydrogenase (*GAPDH*) gene was the internal control to normalize the expression levels of the target genes. Relative gene expression quantification was calculated using the 2^−ΔΔCt^ method, with normalization to the GAPDH levels in mice. All qRT-PCR assays were carried out in triplicate to ensure accuracy and reproducibility.

### Statistical analysis

Statistical analyses were performed using IBM SPSS software (version 26.0; IBM Corporation, Armonk, NY, USA). The data are expressed as mean values±standard error for each group (n = 3) and were compared between groups utilising Student’s t-test. Statistical significance was defined as * p< 0.05, ** p<0.01, and *** p<0.001.

## RESULTS

### Effect of probiotics *Lactobacillus* spp. on body weight of mice

The body weight of mice significantly increased in both groups on a weekly basis (Supplementary Figure S1). As a result of the multiple comparisons, there was no significant difference in body weight between the two groups before starting this experiment. In contrast, body weight after one week was significantly different between all groups and higher in the probiotic group than control groups (Supplementary Figure S1). During two weeks of body weight rate in mice, the control increased by 13.04%, probiotic *Lactobacillus* spp. group had increased 19.64% compared to starting the experiment. In the 3- and 4-weeks, the body weight of mice increased by 20.36%, 26.86%, and 29.75%, 45.77% in the control and probiotic groups, respectively, compared to the first week of the experiment (Supplementary Figure S1) (p<0.05).

### Sequencing and data analysis

To identify differentially expressed mRNAs in the spleen of mice, we constructed 12 sequencing libraries, comprising control day 14 (D14) group (M01, M02, and M03), D28 group (M07, M08, and M09), probiotics *Lactobacillus* spp. D14 group (M14, M15, and M16), and probiotics *Lactobacillus* spp. D28 group (M22, M24, and M25). A total of 152,675,128, 162,241,458, 42,600,826, and 147,919,264 raw reads were generated from the control D14, control D28, probiotics D14, and probiotics D28 groups, respectively (Supplementary Table S1). After removing interfering data, approximately 141,978,686, 152,198,330, 146,296,178, and 161,187,602 clean reads were obtained, which were then aligned to the whole genome of mice using HISAT2. The Q20 scores of clean bases for all samples exceeded 94.60% to 97.19%, indicating high-quality sequencing data for this study. Multiple quality control assessments were performed to evaluate the RNA-Seq data comprehensively. The gene coverage method was utilized to analyze the results, focusing on the distribution and consistency of read coverage across the genes to ensure accuracy in the data interpretation, revealing consistent patterns in gene coverage and the distribution of distinct reads across all 12 RNA-Seq datasets. Our findings indicated that the ratios of clean reads mapped to the genome ranged from 57.20% to 62.90% (Supplementary Table S1). These gene profiles are significant as variations in gene expression levels among the mice could provide insights into the underlying mechanisms of protective immune response and metabolic synthesis associated with probiotic supplementation in mice.

### Differentially expressed genes between control and probiotics *Lactobacillus* spp. groups

**Figure 1:**
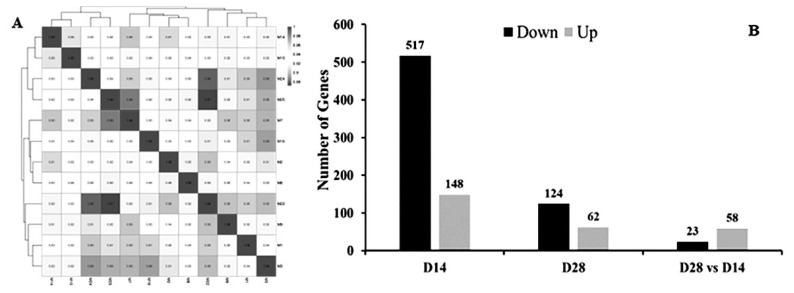
(A) Assessment of RNA-Seq concordance across three groups utilizing Pearson correlation coefficient, a measure of linear association between variables. (B) Evaluation of differentially expressed genes (DEGs) across all groups, with statistical significance thresholds set at p<0.05 and an absolute log2 fold change≥1. The comparison includes day 14 probiotic versus control, day 28 probiotic versus control, and day 28 versus day 14 within the probiotic group.

**Figure 2:**
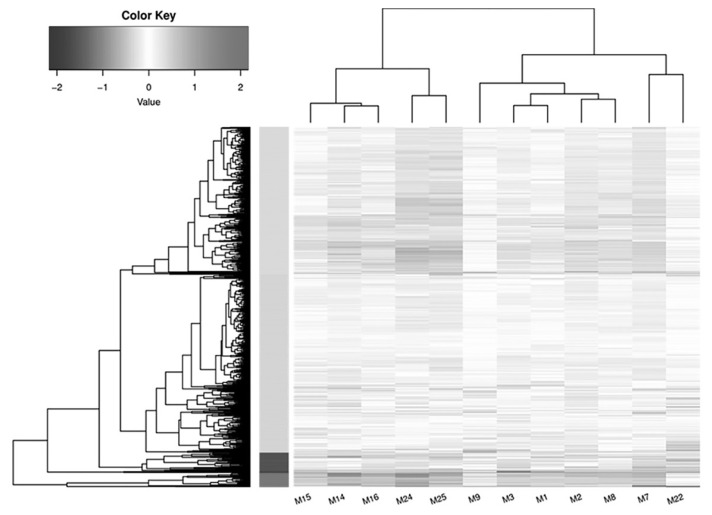
Representation of expressed genes via Heatmap for each group with p<0.05 and Log10(FPKM + 1) values are used for clustering. Genes with increased expression are denoted in black, while those with decreased expression are indicated in white.

The correlation coefficient was calculated between two replicates to assess the consistency across samples. A correlation coefficient close to 1 signifies strong repeatability between two parallel experiments. The analysis revealed that the three biological replicates from each of the four groups demonstrated high repeatability, indicating consistent and reliable experimental results. The R^2^ values ranged from 0.91 to 0.94 for the control D14 group, 0.90 to 0.94 for the control D18 group, 0.92 to 0.96 for the probiotics D14 group, and 0.87 to 0.94 for the probiotics D28 group (Figure 1A). In this study, the mapped reads of each sample were quantified and normalized to FPKM to evaluate gene expression levels. Based on the FPKM values, a total of 30,336 genes were obtained from the 12 sequencing libraries representing the D14- and D28-control and probiotics groups. Among these, 2,945 genes were exclusively expressed in the probiotics group, while 2,416 genes were exclusively expressed in the control group. Additionally, hierarchical clustering clearly delineated the genes/transcripts into distinct groups corresponding to the spleen samples from the control and probiotics groups (Figure 2).

Genes meeting the false discovery rate (FDR) criteria <0.05 and a |log2 fold change| ≥ 2 were deemed DEGs/transcripts, indicating their statistical significance. At day 14 post-induction, a total of 665 genes exhibited differential expression between the control and probiotics groups (Figure 1B). Among these, 148 genes were significantly upregulated with fold changes ranging from 2.01 to 14.0, while 517 genes were significantly downregulated with fold changes ranging from −2.0 to −99.3 (Figure 1B; Supplementary Table S2A). Following 28 days of induction, 186 DEGs were identified between the control and probiotics groups (Figure 1B), with 124 genes downregulated and 62 genes upregulated (Figure 1B; Supplementary Table S2B). Comparison between probiotics groups on days 14 and 24 revealed 58 upregulated and 23 downregulated DEGs (Figure 1B; Supplementary Table S2C).

### Innate immune responses to probiotic *Lactobacillus* spp.-induced mice

**Figure 3:**
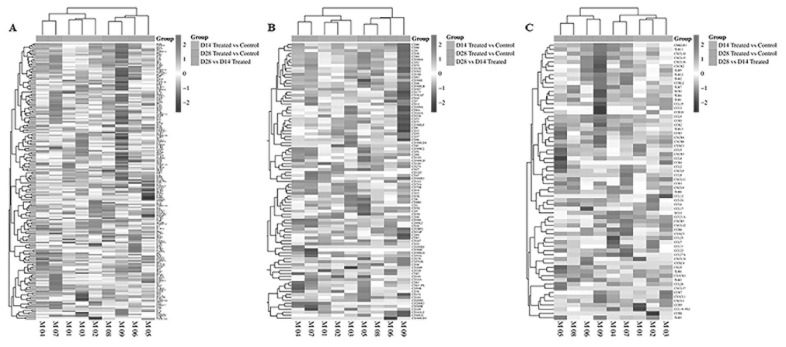
Hierarchical clustering of gene expression for cytokine (A), chemokine (B), and CD molecular genes (C) in spleen samples from mice treated with probiotics. The clusters were generated using Euclidean distance and correlation analyses, with inclusion criteria for significant differential expression set with p<0.01 and |log2 fold change| ≥2). Upregulated Genes are highlighted in black, and downregulated genes are highlighted in white. Day 14 of probiotic vs control group, day 28 of probiotic vs control group, and day 28 vs day 14 probiotic groups.

Transcriptome analysis of 249 cytokines, 61 chemokines, and 102 cluster of differentiation (CD) molecules revealed the fold change induced by probiotics compared to the uninfected control for each mouse, as depicted in Figure 3 and detailed in Supplementary Table S3A–C. In control vs probiotics groups at day 14, the expression of 18 chemokines (such as CXCL2, CXCL5, CCL4, CCL5, CXCL10, etc.) was upregulated, while three chemokines (CCL1, CCL28, and CXCL2) were downregulated, each with p<0.05 and a |log2 fold change| ≥2 (Figure 3C; Supplementary Table S3C). Conversely, the expression of 12 CD molecules (CD3e, CD8a, CD8b1, CD22, etc.) was modulated, with eight CD molecules (CD163, CD59b, CD24a, etc.) significantly downregulated, each with p<0.05 and a |log2 fold change| ≥2 (Figure 3B; Supplementary Table S3B). Furthermore, the expression levels of 28 cytokines/receptors (including IL-1a, IL-10, IL-7, IL-21, IL-27, IFNk, etc.) were notably increased, while 14 cytokines/receptors (such as IL-9R, IL-17D, IL-11RA2, etc.) were significantly decreased in the probiotics groups compared to the control groups, each with a p<0.05 and a |log2 fold change|≥2 (Figure 3A; Supplementary Table S3A).

At day 28 post-induction, comparing probiotics vs control groups revealed significant upregulation in the expression levels of 35 cytokines/receptors (including IL-1B, IL-17F, IL-11, IL-17C, IFNg, etc.), nine chemokines (CCR5, CCL8, CCL3, CXCL9, etc.), and 12 CD molecular genes (*CD69*, *CD163*, *CD46*, etc.). Conversely, six chemokines (CCL1, CCL7, CCL8, CXCL5, CCL22, and CCR1L), six-CD molecular genes (*CD24a*, *CD55B*, *CD300LD2-3*, and *CD209A-F*), and 17 cytokines/receptors (such as IL-4, IL21, IL-24, IL-9R, etc.) were significantly downregulated in the probiotics groups compared to the control groups, each with a p<0.05 and a |log2 fold change| ≥2 (Figure 3; Supplementary Table S3B). Similarly, we observed a notable increase in the expression of six chemokines (CCL8, CXCL3, CXCL9, etc.), 10 CD molecular genes (*CD163*, *CD207*, *CD4*, etc.), and 36 cytokines/receptors (IL-10, IL-11, IL-12B, etc.) with p<0.05 (Figure 3; Supplementary Table S3C). Conversely, the expression of four chemokines (CCR1, CCL22, CCR6, and CCL24), three CD molecular genes (*CD209A-E* and *CD300LD4*), and nine cytokines/receptors (IL-4, IL-21, IL-23R, etc.) was significantly decreased with p<0.05 in the probiotics group at day 28 compared to the probiotics group at day 14 (Figure 3; Supplementary Table S3C).

### Gene ontology enrichment analysis

**Figure 4:**
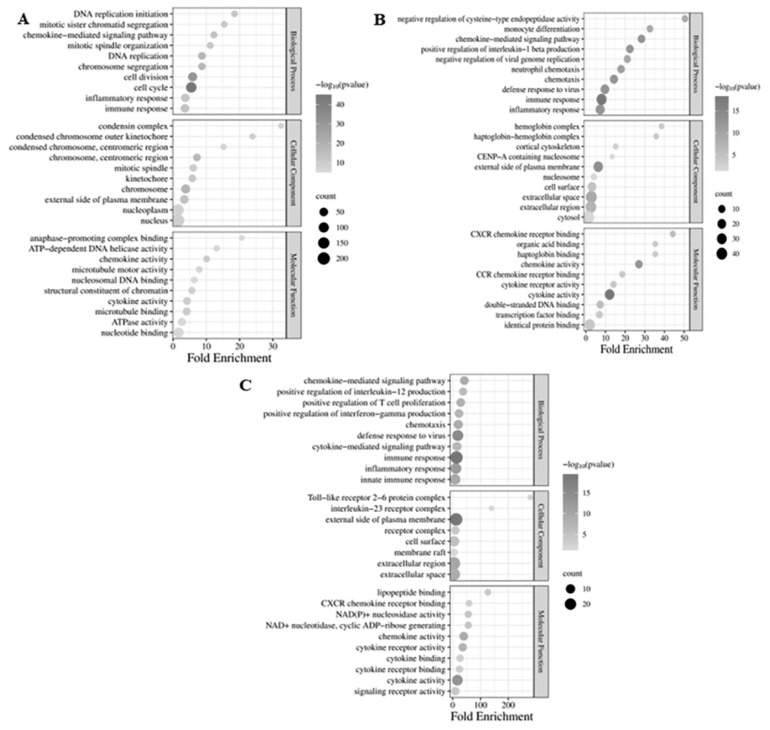
Functional characterization of gene expression alterations in spleen tissue from mice subjected to probiotic treatment via gene ontology (GO) analysis. (A) Predominant 30 enriched GO annotations for differentially expressed genes (DEGs) comparing day 14 probiotic group with controls. (B) Foremost 30 enriched GO annotations for DEGs comparing day 28 probiotic group with controls. (C) Primary 30 enriched GO annotations for DEGs in the day 28 probiotic group relative to day 14.

To delve into the mechanism of probiotics exposed at the genetic level, DEGs between each group underwent analysis of GO enrichment. The GO terms of each group were categorized into processes (BP), molecular functions (MF), and cellular components (CC), respectively (Supplementary Table S4; p<0.05; and number of genes in each GO term >2). The top 10 GO terms of each BP, MF, and CC are illustrated in Figure 4. A total of 665 DEGs were subjected to GO enrichment analysis between the probiotics and control groups on day 14. The BP, MF, and CC terms were further divided into 203, 61, and 62 subterms, respectively (Supplementary Table S4A; p<0.05; and number of genes in each GO term >2). Most of the DEGs were found to be associated with subcategories such as immune response, cell cycle, chromosome organization, external side of the plasma membrane, microtubule binding, and chemokine/cytokine activity (Figure 4A). Conversely, a total of 186 DEGs were analyzed for GO enrichment between the probiotics and control groups on day 28. The BP, MF, and CC terms were divided into 123, 16, and 31 sub-terms, respectively (Supplementary Table S4B; p<0.05; and number of genes in each GO term >2). The majority of DEGs were enriched in subcategories including cellular response, immune response, inflammatory response, external side of the plasma membrane, extracellular space, cytokine, and chemokine activity (Figure 4B). Furthermore, 81 DEGs underwent GO enrichment analysis between the probiotic’s groups on day 28 and day 14. The BP, MF, and CC terms were categorized into 97, 08, and 19 subcategories, respectively (Supplementary Table S4C; p<0.05; and a number of genes in each GO term >2). The predominant subcategories encompassed immune response, defense response to the virus, external side of the plasma membrane, extracellular region, cytokine, and chemokine activity (Figure 4C).

### Kyoto encyclopedia of genes and genomes pathway analysis

**Figure 5:**
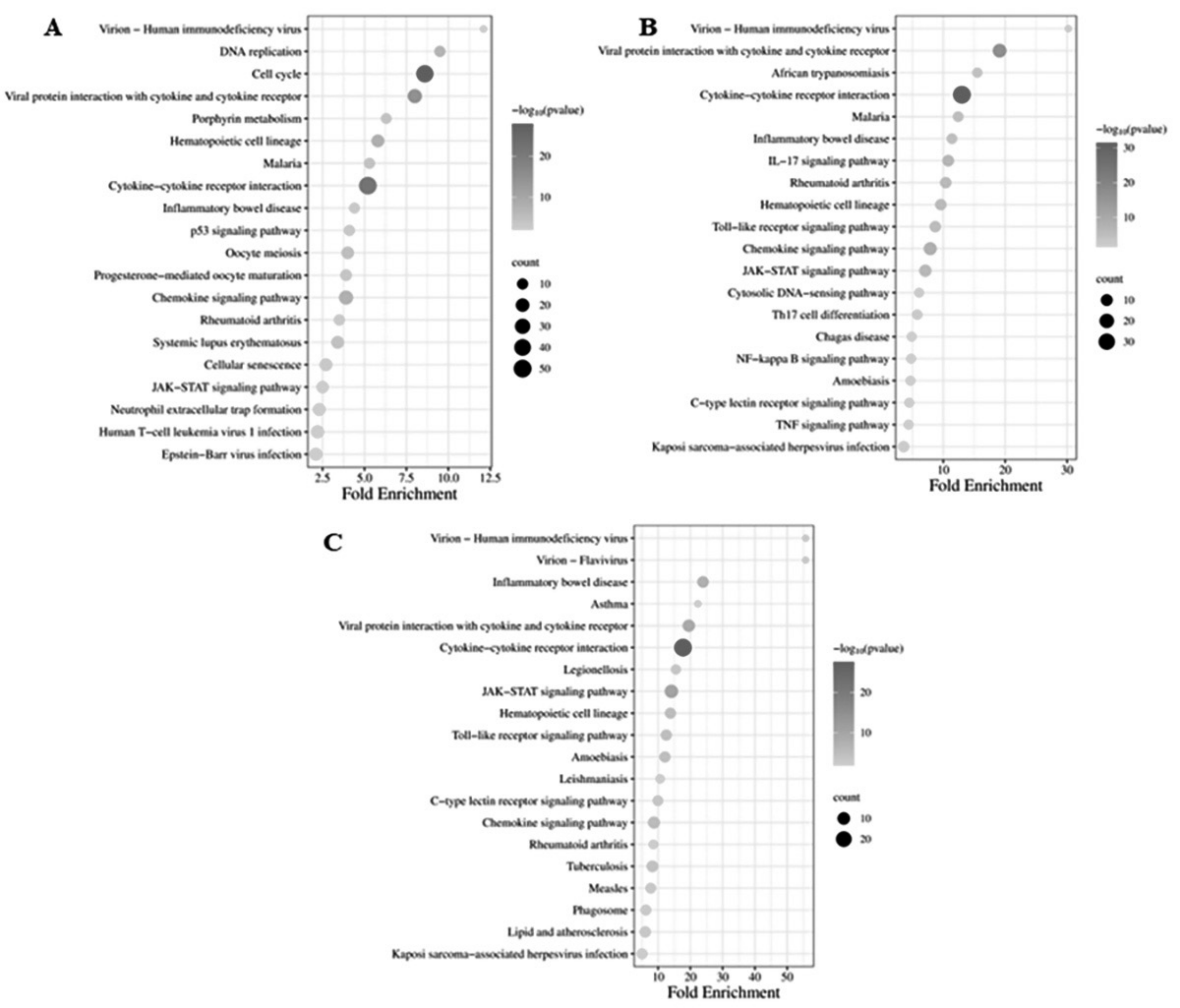
Kyoto encyclopedia of genes and genomes pathway analysis reveals significantly enriched routes in differentially expressed genes (DEGs) within the spleen tissues of chickens receiving probiotics. (A) Top 20 pathways enriched by DEGs comparing day 14 probiotic to control groups. (B) Leading 20 pathways enriched by DEGs comparing day 28 probiotic to control groups. (C) Principal 20 pathways enriched by DEGs between the day 28 and day 14 probiotic groups.

To gain a detailed insight into the functions of the DEGs, we performed a pathway analysis by mapping the DEGs using the KEGG database. This analysis was conducted through DAVID Bioinformatics Resources version 6.7, setting a significance threshold of p<0.01. Throughout the KEGG pathway mapping process, most DEGs were assigned gene identification numbers corresponding to the National Center for Biotechnology Information (NCBI) Gene ID, ensuring accurate gene function association. All DEGs were subjected to KEGG enrichment analysis between the probiotics and control groups on day 14 and day 28, as well as between the probiotics group on day 28 and day 14, with a significance threshold of p<0.05 and a minimum of three genes in each pathway term (Supplementary Table S5). The top 20 most significantly differentially expressed signaling pathways from each group are summarized in Figure 5. In the comparison between probiotics and control groups on day 14, 665 significant DEGs (p<0.05) were enriched in pathways related to immune response (such as cytokine-cytokine receptor interaction, chemokine signaling pathway, p53 signaling pathway, Janus kinase-signal transducers and activators of transcription [JAK-STAT] signaling pathway, or toll-like receptor [TLR] signaling pathway), response to diseases (such as viral protein interaction with cytokine and cytokine receptor, inflammatory bowel disease, rheumatoid arthritis, or human T-cell leukemia virus one infection), and other pathways including cell cycle, hematopoietic cell lineage, DNA replication, or oocyte meiosis (Figure 5A; Supplementary Table S5A). Similarly, in the comparison between the probiotics group and control group on day 28, a total of 186 DEGs (p<0.05) were predominantly associated with immune response pathways (such as cytokine-cytokine receptor interaction, nuclear factor kappa B [NF-kB] signaling pathway, chemokine signaling pathway, IL-17 signaling pathway, JAK-STAT signaling pathway, or TLR signaling pathway), as well as pathways related to diseases such as viral protein interaction with cytokine and cytokine receptor, rheumatoid arthritis, hematopoietic cell lineage, inflammatory bowel disease, or virion - human immunodeficiency virus (Figure 5B; Supplementary Table S5B). Moreover, the comparison between probiotics groups on day 28 and day 14 revealed 81 significant DEGs (p<0.05) primarily associated with immune response pathways (such as cytokine-cytokine receptor interaction, JAK-STAT signaling pathway, chemokine signaling pathway, or TLR signaling pathway), along with pathways related to diseases such as viral protein interaction with cytokine and cytokine receptor, inflammatory bowel disease, amoebiasis, tuberculosis, legionellosis, measles, virion - human immunodeficiency virus, or virion - flavivirus (Figure 5C; Supplementary Table S5C).

### Validation of gene expression by using quantitative real-time polymerase chain reaction

**Figure 6:**
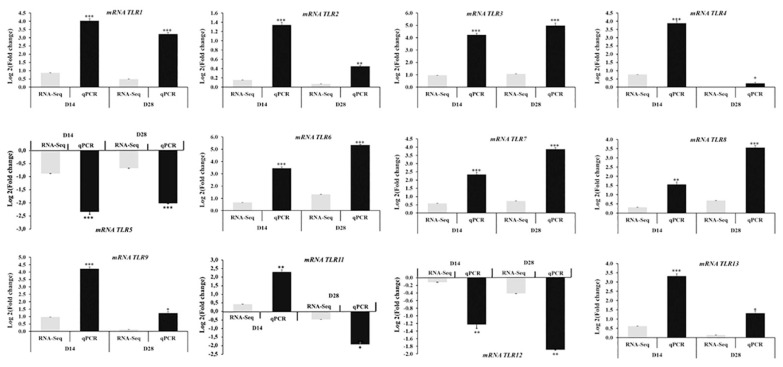
Validation of differentially expressed genes (DEGs) using quantitative real-time polymerase chain reaction (qRT-PCR). The results are presented as mRNA levels normalized against glyceraldehyde 3-phosphate dehydrogenase (GAPDH) mRNA levels. Each measurement was performed in triplicate using pooled samples from three mice. Error bars represent the technical replicates’ standard error, also performed in triplicate. Significant variations in mRNA expression levels between the treatment and control groups are denoted as follows: * p<0.05; ** p<0.01; *** p<0.001.

To validate the expression levels of DEGs identified through RNA-Seq, we conducted qRT-PCR analysis on 12 *TLR* genes (*TLR1*, *TLR2*, *TLR3*, *TLR4*, *TLR5*, *TLR6*, *TLR7*, *TLR8*, *TLR9*, *TLR11*, *TLR12*, and *TLR13*). These genes are pivotal in regulating immune responses and triggering the expression of immune-related genes. As depicted in Figure 6, the qRT-PCR results demonstrated a consistent trend of upregulation or downregulation in the expression levels of these DEGs compared to the RNA-seq data. This consistency between the two methods, qRT-PCR and RNA-Seq, underscores the reliability of the RNA-seq data. Therefore, the RNA-seq data accurately represent the relative expression levels of DEGs in the spleen of mice treated with probiotics *Lactobacillus* isolated in Vietnam.

## DISCUSSION

Probiotics, a group of living microorganisms, offer notable health benefits when appropriately administered [22]. Among the representatives of probiotics are LAB such as *L. acidophilus*, *L. plantarum*, *L. johnsonii*, *L. gasseri*, *L. casei*, *L. rhamnosus*, and *Bifidobacterium longum*, *B. breve*, *B. infantis*, *B. thermophilous*, *B. pseudopodium*, among others [23,24]. These probiotic *Lactobacillus* strains have demonstrated a wide range of benefits in both humans and animals. For instance, certain probiotic *Lactobacillus* strains interact with various immune cells and intestinal mucosal epithelium, thereby modifying host immunity and metabolism [25]. Additionally, certain *Lactobacillus* spp. adhere to intestinal epithelial cells, impeding pathogen attachment and subsequently reducing pathogen survival in the gastrointestinal tract [26]. Moreover, *Lactobacilli* exhibit immunoregulatory properties by initiating immunoregulatory responses and fostering the generation of regulatory dendritic cells and T cells, particularly in conditions marked by dysregulated immunity like allergy, autoimmune polyglandular syndromes, and inflammatory bowel disease [27]. Furthermore, *Lactobacilli* can promote the maturation of DCs, thereby normalizing mucosal immune function during Helicobacter pylori infection, highlighting lactobacilli’s immunostimulatory effects on immune cells [27]. While numerous studies have demonstrated the regulation of gene expression and immune response control by probiotics *Lactobacillus* spp. in mammals, poultry, and fish [26,28], research in Vietnam remains limited. This study investigates the transcriptomic profile in the spleen of mice following exposure to probiotics *Lactobacillus* spp., including four strains: *L. fermentum*, *L. casei*, *L. plantarum*, and *L. brevis*, further affirming the probiotic efficacy of these strains at a molecular level.

A total of 30,336 genes in the control and probiotics group were identified in the spleen of mice, in which 665 (517 downregulated and 148 upregulated) and 186 (124 downregulated and 62 upregulated) DEGs identified from the spleen of mice after 14- and 28-day exposure with probiotics *Lactobacillus* spp., respectively. The GO analysis of DEGs revealed enrichment in categories related to the immune system and defense response to pathogens within the biological process category. Subsequent analysis using the KEGG database identified enrichment of DEGs involved in the spleen’s defense response to viruses and innate immune response processes. Recently, research indicated that the *Lactobacillus* spp., including *L. fermentum*, *L. casei*, *L. plantarum*, and *L. brevis*, induced the immune system and defense response to pathogens such as influenza A virus [29], stress response [30], *Escherichia coli* [31], *salmonella* [32], or acute diarrhea [33]. Prior research has demonstrated that both live and heat-killed *L. rhamnosus* GG can induce the antibacterial functions of macrophages by activating NF-kB, STAT1, and STAT3 DNA-binding activity [23]. Moreover, recent research has shown that four heat-killed lactobacilli strains (*L. fermentum*, *L. casei*, *L. plantarum*, and *L. brevis*) induce early immunostimulatory effects, enhancing the phagocytic and bactericidal activities of human macrophages against various pathogens [8,24,34]. Therefore, based on the results of GO and KEGG analyses, exposure to probiotics *Lactobacillus* spp. significantly impacted defense responses to pathogens (primarily associated with inflammatory bowel diseases, malaria, leukemia virus 1, or herpes virus) and immune processes (mainly implicated in immune response and signal transduction) in the spleen of mice. Consequently, we identified several key DEGs associated with the spleen’s immune response and discussed their potential functions in combating pathogen infections.

The spleen is an important site for the development of immune cells and plays an essential role in the immune system [35]. Our result identified a total of 127 DEGs (68 upregulated and 59 downregulated DEGs) and 89 DEGs in the spleen (55 upregulated and 32 downregulated DEGs) participated in the immune system and immune disease in the spleen of mice at days 14 and 28 after exposure to probiotics *Lactobacillus* spp., respectively. Recently, the function of chemokine and its receptors in the immune response in mammals, chickens, and fish were well investigated. For example, Chemokines, including CCL1–5, CXCL1, CXCL2, CXCL9–11, CXCR3–6, or CCR4–5, are associated with and transmit signals through G–protein coupled receptors. Upon GTP binding to the Ga subunit, they activate various memory T cells such as Th1, Th2, Th17, or Treg cells, thus contributing to innate and adaptive immunity and subsequently participating in defense pathways to establish acquired resistance against Tuberculosis infection [36]. Additionally, they activate and associate with multiple immune or transduced signaling pathways, including PI3K, cytokine-cytokine interaction, JAK-STAT, mitogen-activated protein kinase (MAPK), TLRs, and NF-kB signaling pathways. These pathways and their related cascades play critical roles in cell proliferation, inflammation, migration, motility, and immune responses [37]. Recent studies have revealed that chemokine ligands of CCR2, such as CCL2, CCL7, CCL8, CCL12, CCL13, and CCL16, can be induced by various mediators, including IL-1β, IL-4, TNF-α, TGF-β, IFN-γ, platelet-derived growth factors, and vascular endothelial growth factor [37]. This induction relies on the activation of constitutive NF-κB, PI3K/Akt, p38 MAPK, extracellular signal-regulated kinases (ERK), and JAK-2 signaling pathways, thereby regulating liver pathology and influencing all stages of liver disease progression, from initial injury through inflammation and chronic hepatitis B virus, hepatitis C virus infection to fibrosis/cirrhosis and hepatocarcinogenesis [37]. Moreover, several clinical studies have indicated that chemokines such as IL-8, CCL2, CCL3, CCL7, CCL8, CXCL2, CXCL16, and CX3CL1 act as infiltration signals that facilitate the recruitment of mononuclear phagocytes to the lungs [38]. They are directly involved in the pathogenesis of severe clinical outcomes observed in COVID-19, SARS-CoV, MERS-CoV, Influenza infection, and intestinal disease infections [39]. Our data indicated that a total of 24 DEG of chemokines, of which 3 DEG were downregulated (CCL1, CCL28, and CXCL2) and 21 DEG were upregulated (CCL4, CXCL5, CCR5, CXCR1, CXCL1, etc.) on day 14 in the spleen of mice. After 28 days of exposure to probiotic *Lactobacillus* spp. showed that six DEG chemokines (CCR8, CCL1, CCL7, CXCL5, CCRLl1, and CCL22) were downregulated, and nine DEG chemokines (CCR5, CXCL10, CXCL11, CXCL3, CCL28, CCL3, CXCL17, CXCL9, and CCL8) were upregulated in spleen of mice. These results suggest that probiotics *Lactobacillus* spp. induced the expression of chemokine genes in the spleen of mice and may play an important role in the immune response of mice to pathogen infection.

*Lactobacilli* directly and indirectly affect epithelial cells and various immune cells, including macrophages, dendritic cells, and regulatory T cells. The cell wall components of lactobacilli, such as lipoteichoic acid (LTA), lipopolysaccharides, peptidoglycans, and lipoproteins, are recognized by host cells through pattern recognition receptors, including TLRs and intracellular nucleotide-binding oligomerization domain-like receptors. This recognition initiates the activation of host immune responses [38]. TLRs play a crucial role as pattern recognition receptors in identifying pathogen-associated molecular patterns (PAMPs) from invading pathogens. They are integral to innate and adaptive immune defenses by inducing the synthesis and release of inflammatory cytokines [38]. Studies have shown that *L. casei* NCU011054 upregulates the TLRs/NF-κB pathway, including TLR-2, TLR-4, TLR-6, p65, and NF-κB, as well as two transcription factors, T-bet and GATA-3, mRNA levels, and enhances the number of CD4+ T cells [40,41]. Following treatment with *L. casei* NCU011054, the levels of Th1-related cytokines (IL-12p70, IFN-γ, and TNF-α) and Th2-related cytokines (IL-2, IL-4, IL-6, and IL-10) significantly increase [41]. *L. fermentum* CECT5716 has demonstrated the ability to protect the intestine and lungs and differentially modulate the immune response of intestinal epithelial cells (IECs) triggered by TLR4 activation through regulating negative TLR regulators’ expression [42]. Studies have demonstrated that *L. plantarum* CRL1506 mitigates TLR3-induced small intestinal injury in mice by modulating the production of pro-inflammatory cytokines and the interaction of IECs with intraepithelial lymphocytes [10]. *L. plantarum* ZLP001 has been shown to induce the expression of porcine host defense peptides (HDPs) both *in vivo* and *in vitro*, with this induction seemingly regulated through TLR2 and the ERK1/2/JNK and c-jun/c-fos signaling pathways. The modulation of endogenous HDPs by *L. plantarum* ZLP001 presents a promising strategy for enhancing intestinal health and bolstering diarrhea resistance in weaning piglets [34]. Additionally, *L. brevis* 23017 has been observed to mitigate oxidative stress and inflammation via the MAPK and NF-κB pathways mediated by TLR signaling. The protective effect of *L. brevis* 23017 in mice was linked to the signaling pathway protein p38 MAPK and the phosphorylation levels of NF-κB [43]. Our findings suggest that the upregulation of TLRs such as TLR2, TLR4, TLR6, or TLR8 in the spleen of mice exposed to probiotics *Lactobacillus* spp. at day 14 and day 28 may be associated with resistance mechanisms and innate immune responses across different species. Consequently, further elucidation is needed on the role of TLRs in pathogen response following exposure to probiotics *Lactobacillus* spp. (including *L. fermentum*, *L. casei*, *L. plantarum*, and *L. brevis*).

TNF-α is crucial in immune regulation, and the modulation of its expression by probiotics can lead to immune-suppressive or immune-stimulating effects [44]. Probiotics from the *Lactobacillus* genus, such as *L. rhamnosus* GG, *L. rhamnosus* KLDS, *L. helveticus* IMAU70129, and *L. casei* IMAU60214, have been shown to increase TNF-α and IL-8 levels, maintaining their elevation for up to 24 hours [40]. Additionally, *L. plantarum* K55-5 has exhibited potential for immune induction in immunosuppressed mouse models, with its LTA leading to high TNF-α levels, suggesting its potential use in treating immune disorders [7,45]. The production of TNF-α can involve various proteins in the MAPK signaling pathways, such as p38, JNK, ERK1, and ERK2, and the NF-kB signaling pathway. Probiotics often target these cell-signaling pathways to exert anti-inflammatory activity, particularly inhibiting MAPKs, including JNK, ERK, p38, TLR receptors, and NF-kB. Furthermore, studies have highlighted TNF-α’s role in stimulating epithelial cell proliferation, with probiotics contributing to intestinal epithelial barrier regeneration through positive regulation of TNF-α. By modulating TNF-α, probiotics can enhance innate immune responses and serve as potential immunomodulators in immune-compromised patients [7,45]. GO and KEGG analyses have revealed that probiotics *Lactobacillus* spp. exposed to mice for 14 and 28 days participate in immune processes, focusing mainly on immune response and signal transduction pathways such as MAPK, NF-kB, cytokine-cytokine receptor interaction, and TLR signaling. Moreover, the upregulation of TNF-α expression in the spleen of mice exposed to *Lactobacillus* spp. at day 14 and day 28 suggest the pivotal role of these probiotics in regulating MAPK, NF-kB, cytokine-cytokine receptor interaction, and TLR signaling pathways, thereby playing an important role in the immune response to pathogens.

## CONCLUSION

In summary, we conducted transcriptome analysis of the spleen in mice exposed to probiotics from the *Lactobacillus* spp. (*L. fermentum*, *L. casei*, *L. plantarum*, and *L. brevis*) using RNA-seq. The identified DEGs are essential for understanding the molecular mechanisms underlying probiotic-induced spleen development in mice. KEGG and GO analyses revealed that the DEGs were predominantly enriched in defense responses to pathogens, including inflammatory bowel diseases, malaria, leukaemia virus 1, and herpes virus, as well as immune processes related to immune response and signal transduction. This study represents the first investigation into mice’s gene expression in the spleen exposed to probiotics using *Lactobacillus* spp. isolated from a field strain in Vietnam. The findings from this study highlight the potential of probiotics in modulating gene expression in the spleen, which may have implications for immune function and overall health in mice.

## Data Availability

All raw Illumina sequence data can be obtained freely by contacting Dr. Hoang Vu Dang, email: dangvuhoang@nivr.gov.vn or Dr. La Anh Nguyen, email: laanhnguyen@firi.vn. The BI Gallus database has been uploaded to the Ministry of Science and Technology, Vietnam.

## SUPPLEMENTARY MATERIAL

**Supplementary Figure S1.** Effect of probiotic Lactobacillus spp on body weight during different weeks in mice fed. Data are presented as mean±SD (n = 50); letters with different superscripts are significant at p<0.05.

**Supplementary Table S1.** Summary statistics for sequence quality and alignment information of 12 spleen samples in three groups.

**Supplementary Table S2.** List of differentially expressed genes (DEGs) in spleen tissues of mice exposed to probiotics as (A) day 14 of probiotic vs. control group, (B) day 28 of probiotic vs. control group, and (C) day 28 vs. day 14 probiotic groups

**Supplementary Table S3.** Description of cytokine genes, chemokine genes, and CD molecular genes in spleen tissues of mice exposed to probiotics as (A) day 14 of probiotic vs. control group, (B) day 28 of probiotic vs. control group, and (C) day 28 vs. day 14 probiotic groups

**Supplementary Table S4.** Gene ontology enrichment test in the spleen of mice exposed to probiotics with p<0.05, number of genes in each GO term >2, and Fold change of Log2-treatment/control ≥1; (A) day 14 of probiotic vs. control group, (B) day 28 of probiotic vs. control group, and (C) day 28 vs. day 14 probiotic groups

**Supplementary Table S5.** KEGG enrichment test in the spleen mice exposed to probiotics with p<0.05, number of genes in each pathway term >3 and Fold change of Log2-treatment/control ≥1. (A) day 14 of probiotic vs. control group, (B) day 28 of probiotic vs control group, and (C) day 28 vs day 14 probiotic groups
