# Supplementary material for: Identification of immune-associated genes with altered expression in the spleen of mice enriched with probiotic Lactobacillus species using RNA-seq profiling
Source: Anim Biosci. 2024 Aug 26;38(2):336–49. doi: 10.5713/ab.24.0280 (PMC11725755; doi:10.5713/ab.24.0280)
Supplement: Supplementary file 1 [file ab-24-0280-Supplementary-Fig-1.pdf]

Supplementary Figure S1

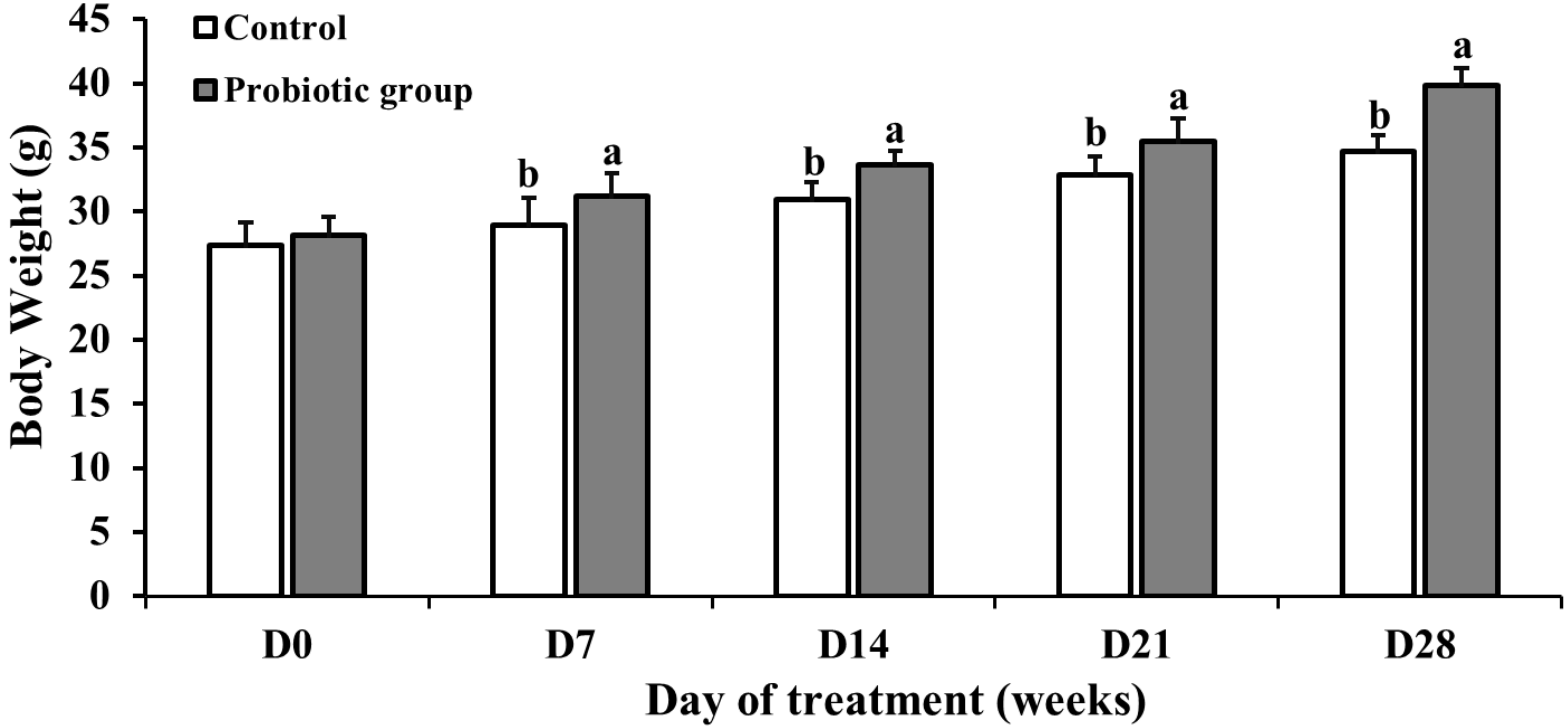

Supplementary Table S1

	Control D14			Control D28			Treatment D14			Treatment D28		
	**M01**	**M02**	**M03**	**M07**	**M08**	**M09**	**M14**	**M15**	**M16**	**M22**	**M24**	**M25**
**Raw data**												
Total Reads	43.199.982	55.730.236	53.744.910	56.381.812	53.241.122	52.618.524	49.116.450	50.564.288	42.920.088	54.506.964	40.844.894	52.567.406
Total Bases	6.479.997.300	8.359.535.400	8.061.736.500	8.457.271.800	7.986.168.300	7.892.778.600	7.367.467.500	7.584.643.200	6.438.013.200	8.176.044.600	6.126.734.100	7.885.110.900
Q20 (%)	96,79	96,71	95,87	96,43	96,44	96,42	96,46	96,45	96,44	96,45	94,2	96,31
Q30 (%)	92,62	92,22	90,45	91,97	92,3	92,27	92,14	92,2	92,14	92,18	86,7	92,02
GC (%)	56,63	56,6	57,02	57,54	57,94	57,03	57,33	56,59	56,65	57,76	54,79	56,45
N (ppm)	5,16	4,7	4,12	4,87	4,69	4,81	4,96	4,83	4,95	4,65	2,91	4,81
**Cleaned data**												
Reads	42.911.552	48.823.098	50.244.036	42.643.596	55.395.602	54.159.132	40.657.866	52.214.908	53.423.404	56.031.206	52.918.544	52.237.852
