# Supplementary material for: Identification of immune-associated genes with altered expression in the spleen of mice enriched with probiotic Lactobacillus species using RNA-seq profiling
Source: Anim Biosci. 2024 Aug 26;38(2):336–49. doi: 10.5713/ab.24.0280 (PMC11725755; doi:10.5713/ab.24.0280)
Supplement: Supplementary file 2 [file ab-24-0280-Supplementary-Table-1.pdf]

Supplementary Figure S1

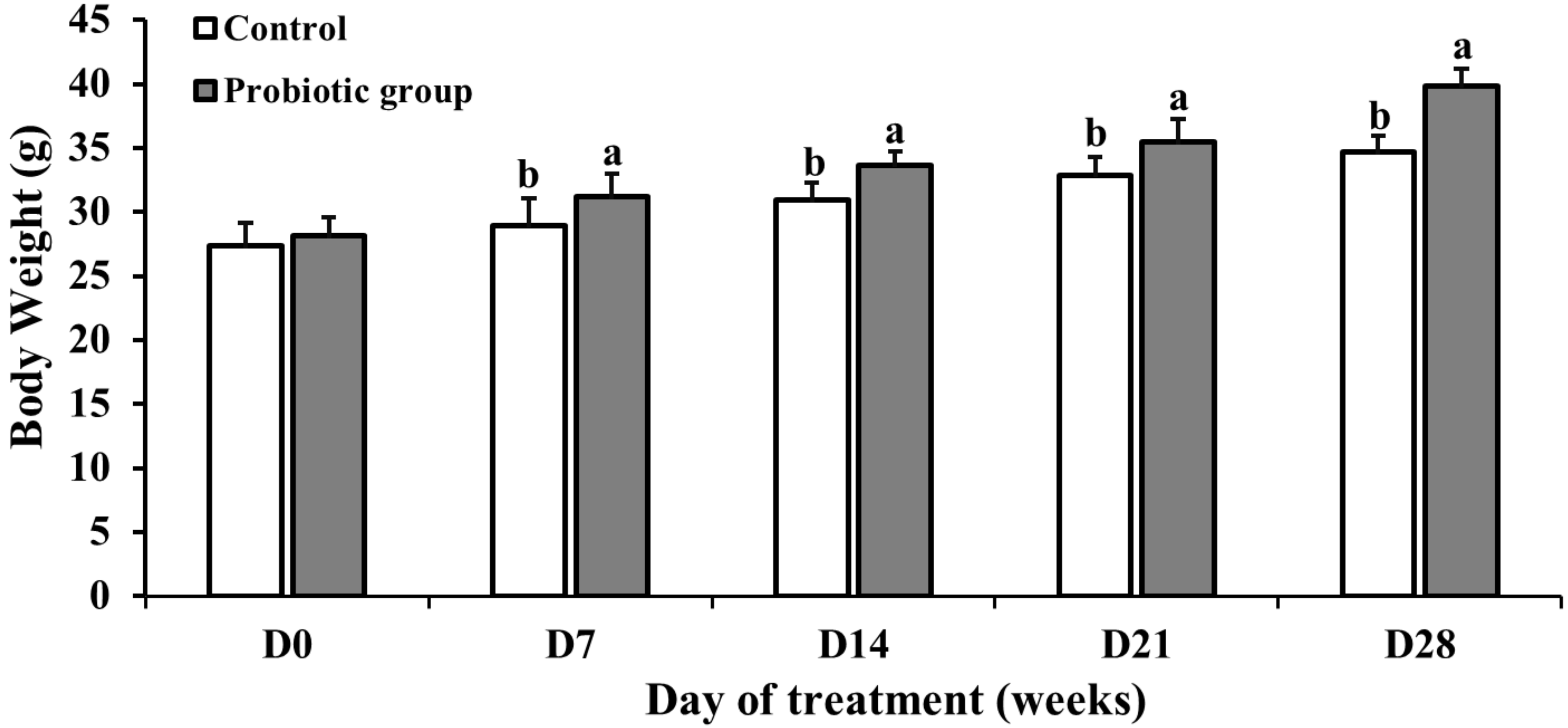

Supplementary Table S1

	Control D14			Control D28			Treatment D14			Treatment D28		
	**M01**	**M02**	**M03**	**M07**	**M08**	**M09**	**M14**	**M15**	**M16**	**M22**	**M24**	**M25**
**Raw data**												
Total Reads	43.199.982	55.730.236	53.744.910	56.381.812	53.241.122	52.618.524	49.116.450	50.564.288	42.920.088	54.506.964	40.844.894	52.567.406
Total Bases	6.479.997.300	8.359.535.400	8.061.736.500	8.457.271.800	7.986.168.300	7.892.778.600	7.367.467.500	7.584.643.200	6.438.013.200	8.176.044.600	6.126.734.100	7.885.110.900
Q20 (%)	96,79	96,71	95,87	96,43	96,44	96,42	96,46	96,45	96,44	96,45	94,2	96,31
Q30 (%)	92,62	92,22	90,45	91,97	92,3	92,27	92,14	92,2	92,14	92,18	86,7	92,02
GC (%)	56,63	56,6	57,02	57,54	57,94	57,03	57,33	56,59	56,65	57,76	54,79	56,45
N (ppm)	5,16	4,7	4,12	4,87	4,69	4,81	4,96	4,83	4,95	4,65	2,91	4,81
**Cleaned data**												
Reads	42.911.552	48.823.098	50.244.036	42.643.596	55.395.602	54.159.132	40.657.866	52.214.908	53.423.404	56.031.206	52.918.544	52.237.852

Bases	6.410.435.567	7.288.621.586	7.498.738.773	6.365.193.942	8.274.715.290	8.083.387.968	6.062.230.310	7.785.794.505	7.970.655.664	8.362.032.636	7.896.179.239	7.796.134.469
Q20 (%)	97,19	96,89	96,9	96,89	97,09	96,91	94,66	96,86	96,34	96,89	96,91	96.92
Q30 (%)	93,09	92,63	92,73	92,65	92,66	92,71	87,18	92,64	90,99	92,49	92,84	92.85
GC (%)	93,09	92,63	92,73	92,65	92,66	92,71	87,18	92,64	90,99	92,49	92,84	92.85
N (ppm)	2,92	2,97	2,94	2,84	2,91	2,99	1,59	2,98	2,58	2,94	2,98	2.93
**Mapped**												
Exon (%)	51,15	53,13	55,62	49,95	48,99	48,7	52,47	52,62	53,55	47,68	58,3	47,96
Intergenic (%)	12,72	11,03	11,04	14,11	13,25	11	14,59	12,22	13,82	13,82	12,78	11,56
Intron (%)	36,23	35,84	33,34	36,44	37,76	40,3	32,94	35,16	32,63	38,5	28,92	40,68
Total mapped	26.707.292 (62.23%)	34.847.313 (62.90%)	32.588.280 (61%)	33.280.729 (59.39%)	30.269.745 (57.20%)	32.634.769 (62.47%)	29.377.573 (60.17%)	31.169.424 (62.03%)	26.059.418 (61.10%)	32.284.297 (59.61%)	27.778.206 (68.32%)	32.419.427 (62.08%)
Multiple mapped	4.456.633 (10.38%)	6.453.548 (11.64%)	6.521.408 (12.20%)	5.427.444 (9.68%)	5.202.060 (9.83%)	6.048.032 (11.57%)	4.044.075 (8.28%)	4.649.951 (9.25%)	3.483.276 (8.16%)	5.094.525 (9.40%)	3.007.180 (7.39%)	5.996.718 (11.48%)
Uniquely mapped	22.250.659 (51.85%)	28.393.765 (51.25%)	26.066.872 (48.79%)	27.853.285 (49.71%)	25.067.685 (47.37%)	26.586.737 (50.89%)	25.333.498 (51.88%)	26.519.473 (52.78%)	22.576.142 (52.94%)	27.189.772 (50.20%)	24.771.026 (60.92%)	26.422.709 (50.60%)
Number of Genes	25177	32609	25476	30792	27904	25754	29880	32003	29509	30919	28839	24828

Supplementary Table S2A

No	log2FoldChange	Regulation	pvalue	GeneSymbol	Description
1	-10,816,970,187,416	Down	0.0005376	Adrb2	adrenergic receptor%2C beta 2 [MGI:87938]
2	-483,417,119,767,682	Down	1.45E-02	Ank1	ankyrin 1%2C erythroid [MGI:88024]
3	-144,527,681,797,704	Down	1.22E-05	Arrb1	arrestin%2C beta 1 [MGI:99473]
4	-325,069,705,693,279	Down	0.00010329	Asic5	acid-sensing (proton-gated) ion channel family member 5 [MGI:1929259]
5	-160,266,512,503,945	Down	2.81E-02	Atp2b4	ATPase%2C Ca++ transporting%2C plasma membrane 4 [MGI:88111]
6	-343,380,632,049,089	Down	0.000127	Cacna1g	calcium channel%2C voltage-dependent%2C T type%2C alpha 1G subunit [MGI:1201678]
7	-241,922,854,405,425	Down	0.003353	Ccl28	chemokine (C-C motif) ligand 28 [MGI:1861731]
8	-214,632,537,480,107	Down	8.49E-29	Ccl1	chemokine (C-C motif) ligand 1 [MGI:98258]
9	-112,781,139,803,128	Down	2.37E+07	Cxcl2	chemokine (C-X-C motif) ligand 2 [MGI:1340094]
10	13,450,487,601,445	Ups	0.026989	Cxcl5	chemokine (C-X-C motif) ligand 5 [MGI:1096868]
11	20,382,068,070,276	Ups	5.99E+09	Ccl24	chemokine (C-C motif) ligand 24 [MGI:1928953]
12	113,220,977,396,178	Ups	0.000435	Ccl19	chemokine (C-C motif) ligand 19 [MGI:1346316]
13	115,043,957,321,004	Ups	2.58E-03	Cxcr6	chemokine (C-X-C motif) receptor 6 [MGI:1934582]
14	116,400,312,581,627	Ups	1.08E+00	Xcl1	chemokine (C motif) ligand 1 [MGI:104593]
15	116,847,167,025,075	Ups	0.01569	Ccl17	chemokine (C-C motif) ligand 17 [MGI:1329039]
16	117,613,726,547,613	Ups	0.0001170	Ccl5	chemokine (C-C motif) ligand 5 [MGI:98262]
17	117,613,726,547,613	Ups	1.05E+08	Ccl5	chemokine (C-C motif) ligand 5 [MGI:98262]
18	122,876,839,245,862	Ups	3.43E+09	Cxcl11	chemokine (C-X-C motif) ligand 11 [MGI:1860203]
