# Supplementary material for: Identification of immune-associated genes with altered expression in the spleen of mice enriched with probiotic Lactobacillus species using RNA-seq profiling
Source: Anim Biosci. 2024 Aug 26;38(2):336–49. doi: 10.5713/ab.24.0280 (PMC11725755; doi:10.5713/ab.24.0280)
Supplement: Supplementary file 3 [file ab-24-0280-Supplementary-Table-2.pdf]

Bases	6.410.435.567	7.288.621.586	7.498.738.773	6.365.193.942	8.274.715.290	8.083.387.968	6.062.230.310	7.785.794.505	7.970.655.664	8.362.032.636	7.896.179.239	7.796.134.469
Q20 (%)	97,19	96,89	96,9	96,89	97,09	96,91	94,66	96,86	96,34	96,89	96,91	96.92
Q30 (%)	93,09	92,63	92,73	92,65	92,66	92,71	87,18	92,64	90,99	92,49	92,84	92.85
GC (%)	93,09	92,63	92,73	92,65	92,66	92,71	87,18	92,64	90,99	92,49	92,84	92.85
N (ppm)	2,92	2,97	2,94	2,84	2,91	2,99	1,59	2,98	2,58	2,94	2,98	2.93
**Mapped**												
Exon (%)	51,15	53,13	55,62	49,95	48,99	48,7	52,47	52,62	53,55	47,68	58,3	47,96
Intergenic (%)	12,72	11,03	11,04	14,11	13,25	11	14,59	12,22	13,82	13,82	12,78	11,56
Intron (%)	36,23	35,84	33,34	36,44	37,76	40,3	32,94	35,16	32,63	38,5	28,92	40,68
Total mapped	26.707.292 (62.23%)	34.847.313 (62.90%)	32.588.280 (61%)	33.280.729 (59.39%)	30.269.745 (57.20%)	32.634.769 (62.47%)	29.377.573 (60.17%)	31.169.424 (62.03%)	26.059.418 (61.10%)	32.284.297 (59.61%)	27.778.206 (68.32%)	32.419.427 (62.08%)
Multiple mapped	4.456.633 (10.38%)	6.453.548 (11.64%)	6.521.408 (12.20%)	5.427.444 (9.68%)	5.202.060 (9.83%)	6.048.032 (11.57%)	4.044.075 (8.28%)	4.649.951 (9.25%)	3.483.276 (8.16%)	5.094.525 (9.40%)	3.007.180 (7.39%)	5.996.718 (11.48%)
Uniquely mapped	22.250.659 (51.85%)	28.393.765 (51.25%)	26.066.872 (48.79%)	27.853.285 (49.71%)	25.067.685 (47.37%)	26.586.737 (50.89%)	25.333.498 (51.88%)	26.519.473 (52.78%)	22.576.142 (52.94%)	27.189.772 (50.20%)	24.771.026 (60.92%)	26.422.709 (50.60%)
Number of Genes	25177	32609	25476	30792	27904	25754	29880	32003	29509	30919	28839	24828

Supplementary Table S2A

No	log2FoldChange	Regulation	pvalue	GeneSymbol	Description
1	-10,816,970,187,416	Down	0.0005376	Adrb2	adrenergic receptor%2C beta 2 [MGI:87938]
2	-483,417,119,767,682	Down	1.45E-02	Ank1	ankyrin 1%2C erythroid [MGI:88024]
3	-144,527,681,797,704	Down	1.22E-05	Arrb1	arrestin%2C beta 1 [MGI:99473]
4	-325,069,705,693,279	Down	0.00010329	Asic5	acid-sensing (proton-gated) ion channel family member 5 [MGI:1929259]
5	-160,266,512,503,945	Down	2.81E-02	Atp2b4	ATPase%2C Ca++ transporting%2C plasma membrane 4 [MGI:88111]
6	-343,380,632,049,089	Down	0.000127	Cacna1g	calcium channel%2C voltage-dependent%2C T type%2C alpha 1G subunit [MGI:1201678]
7	-241,922,854,405,425	Down	0.003353	Ccl28	chemokine (C-C motif) ligand 28 [MGI:1861731]
8	-214,632,537,480,107	Down	8.49E-29	Ccl1	chemokine (C-C motif) ligand 1 [MGI:98258]
9	-112,781,139,803,128	Down	2.37E+07	Cxcl2	chemokine (C-X-C motif) ligand 2 [MGI:1340094]
10	13,450,487,601,445	Ups	0.026989	Cxcl5	chemokine (C-X-C motif) ligand 5 [MGI:1096868]
11	20,382,068,070,276	Ups	5.99E+09	Ccl24	chemokine (C-C motif) ligand 24 [MGI:1928953]
12	113,220,977,396,178	Ups	0.000435	Ccl19	chemokine (C-C motif) ligand 19 [MGI:1346316]
13	115,043,957,321,004	Ups	2.58E-03	Cxcr6	chemokine (C-X-C motif) receptor 6 [MGI:1934582]
14	116,400,312,581,627	Ups	1.08E+00	Xcl1	chemokine (C motif) ligand 1 [MGI:104593]
15	116,847,167,025,075	Ups	0.01569	Ccl17	chemokine (C-C motif) ligand 17 [MGI:1329039]
16	117,613,726,547,613	Ups	0.0001170	Ccl5	chemokine (C-C motif) ligand 5 [MGI:98262]
17	117,613,726,547,613	Ups	1.05E+08	Ccl5	chemokine (C-C motif) ligand 5 [MGI:98262]
18	122,876,839,245,862	Ups	3.43E+09	Cxcl11	chemokine (C-X-C motif) ligand 11 [MGI:1860203]

No	log2FoldChange	Regulation	pvalue	GeneSymbol	Description
19	124,406,146,179,967	Ups	7.81E+07	Cxcl10	chemokine (C-X-C motif) ligand 10 [MGI:1352450]
20	125,177,214,306,982	Ups	2.90E+04	Ccl7	chemokine (C-C motif) ligand 7 [MGI:99512]
21	148,822,594,240,585	Ups	1.37E+09	Ccl4	chemokine (C-C motif) ligand 4 [MGI:98261]
22	150,841,866,476,315	Ups	0.0002512	Ccr5	chemokine (C-C motif) receptor 5 [MGI:107182]
23	150,841,866,476,315	Ups	0.0189967	Ccr5	chemokine (C-C motif) receptor 5 [MGI:107182]
24	156,939,248,347,876	Ups	0.0032476	Cxcl9	chemokine (C-X-C motif) ligand 9 [MGI:1352449]
25	162,786,819,626,243	Ups	7.73E-16	Cxcr1	chemokine (C-X-C motif) receptor 1 [MGI:2448715]
26	162,786,819,626,243	Ups	0.002035	Ccr1l1	chemokine (C-C motif) receptor 1-like 1 [MGI:104617]
27	170,789,845,406,505	Ups	2.89E+07	Ccr4	chemokine (C-C motif) receptor 4 [MGI:107824]
28	208,591,742,387,313	Ups	0.001313	Cxcl1	chemokine (C-X-C motif) ligand 1 [MGI:108068]
29	208,591,742,387,313	Ups	6.77E+09	Cxcl1	chemokine (C-X-C motif) ligand 1 [MGI:108068]
30	273,564,247,573,611	Ups	0.005798	Ccr10	chemokine (C-C motif) receptor 10 [MGI:1096320]
31	-15,371,652,903,832	Down	9.99E+03	Ccnb1	cyclin B1 [MGI:88302]
32	-20,474,871,582,078	Down	1.02E-05	Ccnb2	cyclin B2 [MGI:88311]
33	-256,347,836,955,174	Down	1.63E+08	Ccne1	cyclin E1 [MGI:88316]
34	-210,805,588,839,721	Down	6.09E+00	Ccne2	cyclin E2 [MGI:1329034]
35	-116,963,989,627,878	Down	1.01E+09	Cd1d2	CD1d2 antigen [MGI:107675]
36	269,341,761,322,336	Ups	5.60E-03	Cd209e	CD209e antigen [MGI:2157948]
37	-187,465,718,291,774	Down	4.75E+07	Cd209f	CD209f antigen [MGI:1916392]
38	-112,781,139,803,128	Down	0.010773	Cd209g	CD209g antigen [MGI:1917442]
39	119,758,325,880,075	Ups	5.28E+08	Cd22	CD22 antigen [MGI:88322]
40	119,758,325,880,075	Ups	0.002727	Cd22	CD22 antigen [MGI:88322]
41	143,210,544,215,653	Ups	5.41E+08	Cd274	CD274 antigen [MGI:1926446]
42	112,232,171,827,061	Ups	0.000315	Cd3e	CD3 antigen%2C epsilon polypeptide [MGI:88332]
43	112,232,171,827,061	Ups	0.000492	Cd3e	CD3 antigen%2C epsilon polypeptide [MGI:88332]
44	111,441,055,724,983	Ups	1.63E+02	Cd8a	CD8 antigen%2C alpha chain [MGI:88346]
45	125,198,326,895,244	Ups	0.0001031	Cd8b1	CD8 antigen%2C beta chain 1 [MGI:88347]
46	-280,245,363,060,942	Down	4.72E-29	Cdc25b	cell division cycle 25B [MGI:99701]
47	-18,355,247,747,655	Down	2.27E+00	Cdk1	cyclin-dependent kinase 1 [MGI:88351]
48	-132,676,514,890,766	Down	5.80E+06	Cdkn2c	cyclin dependent kinase inhibitor 2C [MGI:105388]
49	-153,647,820,458,351	Down	1.81E+04	Cdkn2d	cyclin dependent kinase inhibitor 2D [MGI:105387]
50	-19,730,739,152,301	Down	0.0001028	Chek1	checkpoint kinase 1 [MGI:1202065]
51	-481,014,411,117,811	Down	0.001707	Cldn13	claudin 13 [MGI:1913102]
52	110,689,049,484,825	Ups	1.12E+07	Csf2rb	colony stimulating factor 2 receptor%2C beta%2C low-affinity (granulocyte-macrophage) [MGI:1339759]
53	130,101,879,232,537	Ups	0.000733	Csf2rb2	colony stimulating factor 2 receptor%2C beta 2%2C low-affinity (granulocyte-macrophage) [MGI:1339760]
54	111,664,005,416,347	Ups	0.0020476	Csf3r	colony stimulating factor 3 receptor (granulocyte) [MGI:1339755]
55	-182,558,204,776,813	Down	9.46E+07	Dusp8	dual specificity phosphatase 8 [MGI:106626]
56	-160,469,510,558,477	Down	1.22E+08	E2f1	E2F transcription factor 1 [MGI:101941]
57	-290,339,428,310,646	Down	8.88E-40	E2f2	E2F transcription factor 2 [MGI:1096341]
58	106,085,346,799,704	Ups	0.000155	Elmo1	engulfment and cell motility 1 [MGI:2153044]

No	log2FoldChange	Regulation	pvalue	GeneSymbol	Description
59	-249,505,631,846,327	Down	9.47E-06	Epor	erythropoietin receptor [MGI:95408]
60	-178,022,437,573,859	Down	1.42E+04	Fabp1	fatty acid binding protein 1%2C liver [MGI:95479]
61	-10,311,790,637,486	Down	0.000232	Fabp2	fatty acid binding protein 2%2C intestinal [MGI:95478]
62	-165,762,068,924,723	Down	0.001192	Fabp5	fatty acid binding protein 5%2C epidermal [MGI:101790]
63	200,898,437,183,393	Ups	0.001468	Fgf21	fibroblast growth factor 21 [MGI:1861377]
64	-482,205,864,873,027	Down	5.20E+07	Gfap	glial fibrillary acidic protein [MGI:95697]
65	-853,289,592,018,369	Down	0.002381	Gm5771	predicted gene 5771 [MGI:3646222]
66	-128,059,986,363,921	Down	0.0035723	Gtse1	G two S phase expressed protein 1 [MGI:1352755]
67	104,066,515,617,428	Ups	2.98E+08	H2-DMa	histocompatibility 2%2C class II%2C locus DMa [MGI:95921]
68	189,601,373,568,703	Ups	0.000506	H2-Eb2	histocompatibility 2%2C class II antigen E beta2 [MGI:95902]
69	112,039,646,665,143	Ups	0.0001015	H2-Q4	histocompatibility 2%2C Q region locus 4 [MGI:95933]
70	140,380,121,256,121	Ups	4.78E+09	H2-Q6	histocompatibility 2%2C Q region locus 6 [MGI:95935]
71	-11,093,310,508,799	Down	7.52E+09	Hbegf	heparin-binding EGF-like growth factor [MGI:96070]
72	-183,571,877,542,961	Down	6.75E-01	Hmmr	hyaluronan mediated motility receptor (RHAMM) [MGI:104667]
73	122,982,285,788,749	Ups	0.032189	Ifi205	interferon activated gene 205 [MGI:101847]
74	113,686,099,434,364	Ups	0.0009283	Ifi211	interferon activated gene 211 [MGI:3041120]
75	168,109,598,074,735	Ups	0.00391	Ifnk	interferon kappa [MGI:2683287]
76	134,251,419,581,982	Ups	2.84E+06	Il10	interleukin 10 [MGI:96537]
77	-112,781,139,803,128	Down	2.20E-58	Il11ra2	interleukin 11 receptor%2C alpha chain 2 [MGI:109123]
78	-107,338,639,321,485	Down	0.03074	Il17d	interleukin 17D [MGI:2446510]
79	126,642,230,201,673	Ups	2.36E+08	Il18r1	interleukin 18 receptor 1 [MGI:105383]
80	120,258,689,091,241	Ups	0.003767	Il18rap	interleukin 18 receptor accessory protein [MGI:1338888]
81	120,258,689,091,241	Ups	3.52E+04	Il18rap	interleukin 18 receptor accessory protein [MGI:1338888]
82	122,899,081,969,256	Ups	4.45E+00	Il1a	interleukin 1 alpha [MGI:96542]
83	-188,637,578,938,601	Down	0.000149	Il1rl1	interleukin 1 receptor-like 1 [MGI:98427]
84	-188,637,578,938,601	Down	0.005873	Il1rl1	interleukin 1 receptor-like 1 [MGI:98427]
85	176,485,807,999,696	Ups	0.003010	Il21	interleukin 21 [MGI:1890474]
86	230,066,482,602,652	Ups	7.27E+02	Il31ra	interleukin 31 receptor A [MGI:2180511]
87	130,453,096,106,341	Ups	1.15E+03	Il36g	interleukin 36G [MGI:2449929]
88	-20,476,862,561,024	Down	0.000175	Il36rn	interleukin 36 receptor antagonist [MGI:1859325]
89	130,036,094,444,114	Ups	5.13E+04	Il7	interleukin 7 [MGI:96561]
90	-111,699,687,487,171	Down	1.86E+07	Il9r	interleukin 9 receptor [MGI:96564]
91	140,084,799,393,161	Ups	0.000230	Inhba	inhibin beta-A [MGI:96570]
92	-21,723,990,655,809	Down	7.06E+07	Isg15	ISG15 ubiquitin-like modifier [MGI:1855694]
93	10,985,340,770,796	Ups	7.35E+05	Itgal	integrin alpha L [MGI:96606]
94	170,268,517,632,289	Ups	0.000198	Itk	IL2 inducible T cell kinase [MGI:96621]
95	125,459,074,052,249	Ups	0.002506	Lbp	lipopolysaccharide binding protein [MGI:1098776]
96	190,531,892,507,083	Ups	9.49E-01	Lepr	leptin receptor [MGI:104993]
97	195,606,823,170,368	Ups	6.82E+09	Mcpt8	mast cell protease 8 [MGI:1261780]
98	318,990,894,151,124	Ups	0.000936	Mef2b	myocyte enhancer factor 2B [MGI:104526]

No	log2FoldChange	Regulation	pvalue	GeneSymbol	Description
99	-244,778,480,201,225	Down	3.49E+04	Mylk3	myosin light chain kinase 3 [MGI:2443063]
100	206,307,427,830,722	Ups	6.11E+08	Nr4a1	nuclear receptor subfamily 4%2C group A%2C member 1 [MGI:1352454]
101	140,807,587,691,228	Ups	0.000233	Nrg4	neuregulin 4 [MGI:1933833]
102	-123,404,089,932,791	Down	5.24E+06	Oas3	2'-5' oligoadenylate synthetase 3 [MGI:2180850]
103	117,445,939,106,954	Ups	6.14E+09	Pik3r5	phosphoinositide-3-kinase regulatory subunit 5 [MGI:2443588]
104	-133,287,965,835,189	Down	5.07E+06	Plk1	polo like kinase 1 [MGI:97621]
105	-104,251,762,395,897	Down	0.001579	Plk4	polo like kinase 4 [MGI:101783]
106	-140,973,404,555,974	Down	6.19E+03	Ppp2r5b	protein phosphatase 2%2C regulatory subunit B'%2C beta [MGI:2388480]
107	-119,274,080,025,242	Down	8.67E+08	Pttg1	pituitary tumor-transforming gene 1 [MGI:1353578]
108	-127,930,415,599,265	Down	6.07E+02	Rb1	RB transcriptional corepressor 1 [MGI:97874]
109	-147,966,832,814,432	Down	1.05E+03	Slc7a5	solute carrier family 7 (cationic amino acid transporter%2C y+ system)%2C member 5 [MGI:1298205]
110	-17,729,189,061,948	Down	4.89E-01	Steap3	STEAP family member 3 [MGI:1915678]
111	-100,576,004,285,979	Down	9.21E+05	Stradb	STE20-related kinase adaptor beta [MGI:2144047]
112	-569,990,542,082,376	Down	2.18E+09	Tac2	tachykinin 2 [MGI:98476]
113	-256,054,212,040,914	Down	4.10E-24	Tfrc	transferrin receptor [MGI:98822]
114	127,345,586,880,791	Ups	0.016243	Tgif2-ps2	TGFB-induced factor homeobox 2%2C pseudogene 2 [MGI:3805950]
115	-110,926,096,539,279	Down	0.02328	Tlr5	toll-like receptor 5 [MGI:1858171]
116	177,345,566,054,297	Ups	1.32E-06	TnfA	tumor necrosis factor alpha [MGI:104798]
117	-12,835,308,350,574	Down	1.30E+08	Tnfrsf14	tumor necrosis factor receptor superfamily%2C member 14 (herpesvirus entry mediator) [MGI:2675303]
118	-12,835,308,350,574	Down	7.10E+09	Tnfrsf14	tumor necrosis factor receptor superfamily%2C member 14 (herpesvirus entry mediator) [MGI:2675303]
119	111,907,388,148,338	Ups	2.09E+05	Tnfrsf19	tumor necrosis factor receptor superfamily%2C member 19 [MGI:1352474]
120	138,654,913,882,774	Ups	9.37E+02	Tnfrsf26	tumor necrosis factor receptor superfamily%2C member 26 [MGI:2651928]
121	126,360,264,547,992	Ups	0.002553	Tnfrsf4	tumor necrosis factor receptor superfamily%2C member 4 [MGI:104512]
122	140,756,226,052,026	Ups	0.0083200	Tnfrsf8	tumor necrosis factor receptor superfamily%2C member 8 [MGI:99908]
123	201,992,426,611,829	Ups	1.83E-01	Tnfrsf9	tumor necrosis factor receptor superfamily%2C member 9 [MGI:1101059]
124	-124,549,286,001,411	Down	3.37E+04	Tnfsf11	tumor necrosis factor (ligand) superfamily%2C member 11 [MGI:1100089]
125	-112,781,139,803,128	Down	0.000382	Tnfsf4	tumor necrosis factor (ligand) superfamily%2C member 4 [MGI:104511]
126	167,968,548,512,929	Ups	1.48E+02	Tnfsf9	tumor necrosis factor (ligand) superfamily%2C member 9 [MGI:1101058]
127	-111,930,677,425,682	Down	1.03E+04	Tubb4b	tubulin%2C beta 4B class IVB [MGI:1915472]
128	-434,243,342,271,257	Down	3.56E+03	-	novel transcript
129	-273,889,109,176,667	Down	0.000376	-	novel transcript%2C sense intronic to KO:Slc15a2and Slc15a2
130	-104,048,628,563,434	Down	0.00049	-	-
131	-341,077,137,026,181	Down	0.00049	-	novel transcript%2C antisense to KO:Ttll12and Ttll12
132	-351,528,111,201,895	Down	0.001086	-	novel transcript%2C antisense to KO:Pabpc4and Pabpc4
133	-411,236,887,617,685	Down	0.001548	-	novel transcript%2C antisense to Gabbr2
134	179,100,280,513,905	Ups	0.002734	-	novel transcript%2C antisense to Gnat1
135	-137,309,207,578,791	Down	1.43E+04	1700037H04Rik	RIKEN cDNA 1700037H04 gene [MGI:1914576]
136	-555,429,592,465,739	Down	1.15E-02	5430401H09Rik	RIKEN cDNA 5430401H09 gene [MGI:1918646]
137	-639,179,289,803,535	Down	4.50E+07	6030468B19Rik	RIKEN cDNA 6030468B19 gene [MGI:1924977]
138	-454,409,961,145,704	Down	0.0001321	A730036I17Rik	RIKEN cDNA A730036I17 gene [MGI:3041182]

No	log2FoldChange	Regulation	pvalue	GeneSymbol	Description
139	-342,837,596,295,548	Down	8.57E+08	A930006K02Rik	RIKEN cDNA A930006K02 gene [MGI:1925683]
140	-259,310,233,630,567	Down	7.90E-31	Abcb10	ATP-binding cassette%2C sub-family B (MDR/TAP)%2C member 10 [MGI:1860508]
141	-336,704,415,070,037	Down	0.0002556	Abcg4	ATP binding cassette subfamily G member 4 [MGI:1890594]
142	108,963,495,754,287	Ups	7.61E+04	Acaca	acetyl-Coenzyme A carboxylase alpha [MGI:108451]
143	-149,004,845,468,933	Down	1.20E+09	Ache	acetylcholinesterase [MGI:87876]
144	-314,251,066,600,075	Down	3.38E+02	Ackr1	atypical chemokine receptor 1 (Duffy blood group) [MGI:1097689]
145	-391,252,668,822,265	Down	2.04E+07	Add2	adducin 2 (beta) [MGI:87919]
146	-101,310,507,924,452	Down	0.000292	Adh4	alcohol dehydrogenase 4 (class II)%2C pi polypeptide [MGI:1349472]
147	-163,885,346,479,355	Down	5.59E+07	Agpat4	1-acylglycerol-3-phosphate O-acyltransferase 4 (lysophosphatidic acid acyltransferase%2C delta) [MGI:1915512]
148	-116,806,433,593,855	Down	3.57E+04	Alad	aminolevulinate%2C delta-%2C dehydratase [MGI:96853]
149	-346,721,610,051,237	Down	5.85E-62	Alas2	aminolevulinic acid synthase 2%2C erythroid [MGI:87990]
150	112,608,712,687,158	Ups	1.12E+08	Alox5ap	arachidonate 5-lipoxygenase activating protein [MGI:107505]
151	-128,558,398,542,388	Down	2.72E+05	Ammecr1	Alport syndrome%2C mental retardation%2C midface hypoplasia and elliptocytosis chromosomal region gene 1 [MGI:1860206]
152	-155,611,997,421,526	Down	0.001416	Angptl7	angiopoietin-like 7 [MGI:3605801]
153	136,624,408,062,102	Ups	0.0020184	Angptl8	angiopoietin-like 8 [MGI:3643534]
154	-251,522,226,119,751	Down	4.72E+08	Ankle1	ankyrin repeat and LEM domain containing 1 [MGI:1918775]
155	-171,060,770,691,226	Down	1.75E+08	Ankrd9	ankyrin repeat domain 9 [MGI:1921501]
156	-20,226,253,258,078	Down	4.83E-02	Anln	anillin%2C actin binding protein [MGI:1920174]
157	-126,638,298,373,307	Down	0.0017058	Aplf	aprataxin and PNKP like factor [MGI:1919353]
158	-400,360,210,191,041	Down	1.43E+08	Apol11a	apolipoprotein L 11a [MGI:3649094]
159	-407,742,055,531,814	Down	5.38E-42	Apol11b	apolipoprotein L 11b [MGI:3036248]
160	-562,325,824,226,481	Down	1.30E+00	Apol8	apolipoprotein L 8 [MGI:2444921]
161	-109,944,638,489,442	Down	2.31E+03	Arf5	ADP-ribosylation factor 5 [MGI:99434]
162	-128,580,297,579,723	Down	1.49E+09	Arhgap11a	Rho GTPase activating protein 11A [MGI:2444300]
163	-116,273,341,131,115	Down	1.30E+09	Arhgap19	Rho GTPase activating protein 19 [MGI:1918335]
164	-104,754,486,648,274	Down	2.95E+09	Arhgap23	Rho GTPase activating protein 23 [MGI:3697726]
165	109,967,844,944,274	Ups	0.000413	Arl4c	ADP-ribosylation factor-like 4C [MGI:2445172]
166	-158,603,418,042,842	Down	2.42E+08	Art4	ADP-ribosyltransferase 4 [MGI:1202710]
167	-196,035,683,796,039	Down	6.54E-03	Asb1	ankyrin repeat and SOCS box-containing 1 [MGI:1929735]
168	-359,607,725,001,683	Down	0.0008560	Asb17os	ankyrin repeat and SOCS box-containing 17%2C opposite strand [MGI:1919567]
169	-156,219,954,642,057	Down	1.81E+06	Asf1b	anti-silencing function 1B histone chaperone [MGI:1914179]
170	-174,805,716,150,263	Down	4.00E+03	Aspm	abnormal spindle microtubule assembly [MGI:1334448]
171	-102,599,676,294,887	Down	6.46E+05	Atad2	ATPase family%2C AAA domain containing 2 [MGI:1917722]
172	-154,722,729,959,519	Down	1.22E+05	Atad5	ATPase family%2C AAA domain containing 5 [MGI:2442925]
173	-106,736,588,879,489	Down	7.65E+07	Atg4a	autophagy related 4A%2C cysteine peptidase [MGI:2147903]
174	124,691,900,817,654	Ups	0.002131	Atp8b4	ATPase%2C class I%2C type 8B%2C member 4 [MGI:1859664]
175	-10,583,338,222,126	Down	1.29E+06	Atpif1	ATPase inhibitory factor 1 [MGI:1196457]
176	-197,574,654,436,853	Down	2.87E+02	Aurka	aurora kinase A [MGI:894678]
177	-132,922,019,102,689	Down	2.65E+08	Aurkb	aurora kinase B [MGI:107168]
178	-136,740,881,832,979	Down	9.57E+09	AW011738	expressed sequence AW011738 [MGI:2140540]

No	log2FoldChange	Regulation	pvalue	GeneSymbol	Description
179	-123,097,770,522,251	Down	4.39E+06	B4galt5	UDP-Gal:betaGlcNAc beta 1%2C4-galactosyltransferase%2C polypeptide 5 [MGI:1927169]
180	-118,365,467,230,862	Down	0.0008948	Bard1	BRCA1 associated RING domain 1 [MGI:1328361]
181	-252,019,070,948,169	Down	3.74E-09	Birc5	baculoviral IAP repeat-containing 5 [MGI:1203517]
182	-208,518,187,462,503	Down	5.29E+02	Blm	Bloom syndrome%2C RecQ like helicase [MGI:1328362]
183	-153,117,045,434,278	Down	9.99E+00	Blvrb	biliverdin reductase B (flavin reductase (NADPH)) [MGI:2385271]
184	-120,953,009,417,023	Down	7.13E+01	Bnip3l	BCL2/adenovirus E1B interacting protein 3-like [MGI:1332659]
185	-201,512,150,742,514	Down	5.05E-11	Bpgm	2%2C3-bisphosphoglycerate mutase [MGI:1098242]
186	-201,061,613,713,235	Down	3.83E+04	Brca1	breast cancer 1%2C early onset [MGI:104537]
187	-123,646,839,990,213	Down	6.63E+08	Brca2	breast cancer 2%2C early onset [MGI:109337]
188	-150,363,451,755,707	Down	7.97E+00	Brpf3	bromodomain and PHD finger containing%2C 3 [MGI:2146836]
189	-221,046,151,407,855	Down	0.000387	Bsn	bassoon [MGI:1277955]
190	-100,040,956,627,082	Down	1.28E+08	Btg2	BTG anti-proliferation factor 2 [MGI:108384]
191	-540,879,121,867,602	Down	3.00E+05	Btnl10	butyrophilin-like 10 [MGI:2182073]
192	-109,544,249,552,137	Down	2.83E+07	Btrc	beta-transducin repeat containing protein [MGI:1338871]
193	-115,834,779,968,322	Down	0.0011034	Bub1	BUB1%2C mitotic checkpoint serine/threonine kinase [MGI:1100510]
194	106,998,070,068,558	Ups	7.40E+09	Capg	capping protein (actin filament)%2C gelsolin-like [MGI:1098259]
195	-238,775,223,846,982	Down	1.75E-18	Car2	carbonic anhydrase 2 [MGI:88269]
196	-124,595,515,673,024	Down	1.17E+00	Carhsp1	calcium regulated heat stable protein 1 [MGI:1196368]
197	-169,606,525,993,443	Down	0.0001588	Ccdc18	coiled-coil domain containing 18 [MGI:1922974]
198	-104,168,225,090,023	Down	0.0009183	Ccdc34	coiled-coil domain containing 34 [MGI:1915451]
199	-695,172,851,874,073	Down	4.39E+05	Ccdc92b	coiled-coil domain containing 92B [MGI:3588240]
200	-206,386,149,595,676	Down	4.93E-09	Ccna2	cyclin A2 [MGI:108069]
201	-211,696,707,923,297	Down	2.68E-07	Ccnf	cyclin F [MGI:102551]
202	-263,796,676,794,267	Down	7.28E+07	Cd163	CD163 antigen [MGI:2135946]
203	-263,796,676,794,267	Down	2.10E+09	Cd163	CD163 antigen [MGI:2135946]
204	113,999,749,539,974	Ups	0.0087468	Cd177	CD177 antigen [MGI:1916141]
205	134,757,345,757,161	Ups	0.0002374	Cd200r3	CD200 receptor 3 [MGI:1921853]
206	-112,781,139,803,128	Down	0.0066052	Cd200r4	CD200 receptor 4 [MGI:3036289]
207	188,047,703,676,007	Ups	8.08E+05	Cd207	CD207 antigen [MGI:2180021]
208	-287,549,502,780,372	Down	9.68E-35	Cd24a	CD24a antigen [MGI:88323]
209	-287,549,502,780,372	Down	1.85E+00	Cd24a	CD24a antigen [MGI:88323]
210	303,442,553,022,332	Ups	0.0001933	Cd300c	CD300C molecule [MGI:3032626]
211	-112,781,139,803,128	Down	2.56E+09	Cd300ld5	CD300 molecule like family member D5 [MGI:3702661]
212	128,952,839,438,254	Ups	1.04E-06	Cd300lf	CD300 molecule like family member F [MGI:2442359]
213	-177,786,408,180,693	Down	0.00040531	Cd59b	CD59b antigen [MGI:1888996]
214	121,317,090,676,902	Ups	2.95E+08	Cd69	CD69 antigen [MGI:88343]
215	-169,903,082,404,793	Down	0.00290506	Cdc25c	cell division cycle 25C [MGI:88350]
216	-109,473,850,812,251	Down	0.00070248	Cdc45	cell division cycle 45 [MGI:1338073]
217	-230,615,463,403,614	Down	0.00026246	Cdc6	cell division cycle 6 [MGI:1345150]
218	-2,056,870,095,251	Down	9.43E-03	Cdca3	cell division cycle associated 3 [MGI:1315198]

No	log2FoldChange	Regulation	pvalue	GeneSymbol	Description
219	-152,222,094,309,374	Down	3.25E+09	Cdca5	cell division cycle associated 5 [MGI:1915099]
220	-16,446,860,727,343	Down	3.65E+03	Cdca8	cell division cycle associated 8 [MGI:1196274]
221	-181,246,532,634,319	Down	9.53E+09	Cdkn3	cyclin-dependent kinase inhibitor 3 [MGI:1919641]
222	-195,547,131,319,862	Down	5.62E-11	Cdr2	cerebellar degeneration-related 2 [MGI:1100885]
223	-139,524,880,374,581	Down	5.92E+05	Cdt1	chromatin licensing and DNA replication factor 1 [MGI:1914427]
224	-159,729,548,860,146	Down	3.14E+03	Cenpa	centromere protein A [MGI:88375]
225	-173,080,353,861,835	Down	4.80E+01	Cenpe	centromere protein E [MGI:1098230]
226	-231,855,305,506,242	Down	7.28E-22	Cenpf	centromere protein F [MGI:1313302]
227	-191,510,934,479,184	Down	4.00E+09	Cenph	centromere protein H [MGI:1349448]
228	-163,209,918,128,691	Down	0.000168	Cenpk	centromere protein K [MGI:1926210]
229	-171,740,798,257,934	Down	4.08E+09	Cenpm	centromere protein M [MGI:1913820]
230	-118,501,185,400,556	Down	0.0021422	Cenpn	centromere protein N [MGI:1919405]
231	-159,289,415,307,847	Down	0.0004609	Cenpp	centromere protein P [MGI:1913586]
232	-1,041,621,295,179	Down	0.0013864	Cep128	centrosomal protein 128 [MGI:1922466]
233	-177,029,888,394,609	Down	4.51E+01	Cep76	centrosomal protein 76 [MGI:1923401]
234	-189,926,834,540,841	Down	3.67E+06	Cercam	cerebral endothelial cell adhesion molecule [MGI:2139134]
235	151,246,114,179,633	Ups	6.48E+02	Cfh	complement component factor h [MGI:88385]
236	-10,873,272,653,572	Down	5.45E+04	Chac2	ChaC%2C cation transport regulator 2 [MGI:1915294]
237	-113,097,149,851,405	Down	0.0002031	Chaf1a	chromatin assembly factor 1%2C subunit A (p150) [MGI:1351331]
238	-102,688,383,695,243	Down	0.002391	Chaf1b	chromatin assembly factor 1%2C subunit B (p60) [MGI:1314881]
239	118,299,954,404,024	Ups	2.22E+08	Ciita	class II transactivator [MGI:108445]
240	-202,506,372,782,241	Down	4.14E-02	Cit	citron [MGI:105313]
241	-14,782,151,326,746	Down	0.0003016	Cited4	Cbp/p300-interacting transactivator%2C with Glu/Asp-rich carboxy-terminal domain%2C 4 [MGI:1861694]
242	-142,117,373,904,675	Down	5.71E+09	Ckap2	cytoskeleton associated protein 2 [MGI:1931797]
243	-143,945,398,707,593	Down	1.38E+05	Ckap2l	cytoskeleton associated protein 2-like [MGI:1917716]
244	-118,900,012,978,472	Down	1.70E+06	Cks2	CDC28 protein kinase regulatory subunit 2 [MGI:1913447]
245	-154,556,674,348,507	Down	9.72E+07	Clspn	claspin [MGI:2445153]
246	-117,331,596,038,157	Down	5.35E+03	Cmas	cytidine monophospho-N-acetylneuraminic acid synthetase [MGI:1337124]
247	-101,239,782,249,831	Down	1.27E+07	Col3a1	collagen%2C type III%2C alpha 1 [MGI:88453]
248	-129,111,128,188,097	Down	0.003375	Cox6b2	cytochrome c oxidase subunit 6B2 [MGI:3044182]
249	-139,822,465,692,489	Down	9.41E+00	Cpox	coproporphyrinogen oxidase [MGI:104841]
250	-217,279,442,941,864	Down	3.00E+07	Cracd	capping protein inhibiting regulator of actin [MGI:2444817]
251	183,376,987,255,782	Ups	0.0035880	Crybb3	crystallin%2C beta B3 [MGI:102717]
252	-173,018,819,121,458	Down	0.0007940	Csrp3	cysteine and glycine-rich protein 3 [MGI:1330824]
253	-249,341,158,048,745	Down	9.86E-29	Ctse	cathepsin E [MGI:107361]
254	204,876,953,848,562	Ups	2.06E+08	Cyp27b1	cytochrome P450%2C family 27%2C subfamily b%2C polypeptide 1 [MGI:1098274]
255	140,853,929,403,578	Ups	7.37E+04	Cyp2c70	cytochrome P450%2C family 2%2C subfamily c%2C polypeptide 70 [MGI:2385878]
256	226,825,396,638,894	Ups	0.0004966	Cyp2d12	cytochrome P450%2C family 2%2C subfamily d%2C polypeptide 12 [MGI:88604]
257	-143,555,680,037,344	Down	0.0028141	Cyp2f2	cytochrome P450%2C family 2%2C subfamily f%2C polypeptide 2 [MGI:88608]
258	107,839,997,157,827	Ups	6.77E+07	Cyp3a13	cytochrome P450%2C family 3%2C subfamily a%2C polypeptide 13 [MGI:88610]

No	log2FoldChange	Regulation	pvalue	GeneSymbol	Description
259	-17,405,040,784,291	Down	0.00029606	Cyp3a41b	cytochrome P450%2C family 3%2C subfamily a%2C polypeptide 41B [MGI:3714859]
260	-467,440,737,208,487	Down	0.0018468	Cyp4a12b	cytochrome P450%2C family 4%2C subfamily a%2C polypeptide 12B [MGI:3611747]
261	-429,483,228,403,398	Down	0.0006834	Cyp4b1-ps2	cytochrome P450%2C family 4%2C subfamily b%2C polypeptide 1%2C pseudogene 2 [MGI:3649236]
262	-147,365,233,981,481	Down	7.88E+07	D630023F18Rik	RIKEN cDNA D630023F18 gene [MGI:2138198]
263	-122,703,107,488,638	Down	1.34E+09	Dbf4	DBF4 zinc finger [MGI:1351328]
264	-150,721,170,482,911	Down	5.34E+04	Dck	deoxycytidine kinase [MGI:102726]
265	-282,776,342,744,556	Down	2.40E+07	Dclk2	doublecortin-like kinase 2 [MGI:1918012]
266	-120,739,652,676,595	Down	1.31E+03	Dek	DEK proto-oncogene (DNA binding) [MGI:1926209]
267	-108,858,128,209,542	Down	9.13E+06	Dennd4a	DENN/MADD domain containing 4A [MGI:2142979]
268	-246,461,177,643,243	Down	3.48E+05	Depdc1a	DEP domain containing 1a [MGI:1923381]
269	-151,906,460,490,213	Down	2.37E+07	Depdc1b	DEP domain containing 1B [MGI:2145425]
270	-200,213,713,164,137	Down	2.17E-01	Depp1	DEPP1 autophagy regulator [MGI:1918730]
271	-244,238,722,089,009	Down	7.59E-24	Dhrs11	dehydrogenase/reductase (SDR family) member 11 [MGI:2652816]
272	-205,584,341,779,647	Down	7.27E+01	Diaph3	diaphanous related formin 3 [MGI:1927222]
273	-120,802,227,801,871	Down	1.84E+07	Dleu2	deleted in lymphocytic leukemia%2C 2 [MGI:1934030]
274	-200,349,026,353,839	Down	3.75E-01	Dlgap5	DLG associated protein 5 [MGI:2183453]
275	-155,769,270,092,496	Down	1.12E-07	Dmtn	dematin actin binding protein [MGI:99670]
276	-191,935,638,897,566	Down	2.05E+08	Dnaja4	DnaJ heat shock protein family (Hsp40) member A4 [MGI:1927638]
277	-237,971,340,363,704	Down	9.04E+07	Dnajb13	DnaJ heat shock protein family (Hsp40) member B13 [MGI:1916637]
278	-103,104,117,259,883	Down	0.000953	Donson	downstream neighbor of SON [MGI:1890621]
279	195,855,630,079,441	Ups	6.06E+09	Dsg1c	desmoglein 1 gamma [MGI:2664358]
280	-162,336,210,663,483	Down	3.84E+08	Dtl	denticleless E3 ubiquitin protein ligase [MGI:1924093]
281	-226,807,455,453,516	Down	5.66E+05	Dyrk3	dual-specificity tyrosine-(Y)-phosphorylation regulated kinase 3 [MGI:1330300]
282	-108,390,365,303,045	Down	3.75E+07	E2f4	E2F transcription factor 4 [MGI:103012]
283	-133,000,415,383,189	Down	0.0017345	E2f7	E2F transcription factor 7 [MGI:1289147]
284	-199,730,108,621,999	Down	7.69E-01	E2f8	E2F transcription factor 8 [MGI:1922038]
285	-100,402,632,678,239	Down	2.99E+05	Eci3	enoyl-Coenzyme A delta isomerase 3 [MGI:1916373]
286	-134,616,256,289,274	Down	2.52E+05	Ect2	ect2 oncogene [MGI:95281]
287	-138,418,545,723,529	Down	4.34E+03	Ehbp1l1	EH domain binding protein 1-like 1 [MGI:3612340]
288	-117,315,984,231,176	Down	8.17E-02	Eif2ak1	eukaryotic translation initiation factor 2 alpha kinase 1 [MGI:1353448]
289	-116,932,079,570,013	Down	0.000175	Eln	elastin [MGI:95317]
290	-130,524,450,211,244	Down	2.14E+07	Emilin2	elastin microfibril interfacer 2 [MGI:2389136]
291	-171,387,492,163,693	Down	3.58E-02	Endod1	endonuclease domain containing 1 [MGI:1919196]
292	-515,157,579,240,407	Down	1.41E-03	Epb42	erythrocyte membrane protein band 4.2 [MGI:95402]
293	100,115,631,015,582	Ups	5.77E+09	Epsti1	epithelial stromal interaction 1 (breast) [MGI:1915168]
294	183,377,634,828,116	Ups	0.0009091	Epx	eosinophil peroxidase [MGI:107569]
295	-113,428,205,155,491	Down	0.000761	Ercc6l	excision repair cross-complementing rodent repair deficiency complementation group 6 like [MGI:2654144]
296	-119,857,578,956,375	Down	1.51E+09	Erich4	glutamate rich 4 [MGI:3646269]
297	-393,386,943,537,366	Down	4.90E+05	Ermap	erythroblast membrane-associated protein [MGI:1349816]
298	-235,273,893,398,821	Down	7.97E+02	Esco2	establishment of sister chromatid cohesion N-acetyltransferase 2 [MGI:1919238]

No	log2FoldChange	Regulation	pvalue	GeneSymbol	Description
299	-152,600,993,326,165	Down	1.38E+08	Espl1	extra spindle pole bodies 1%2C separase [MGI:2146156]
300	10,821,109,620,107	Ups	0.002960	Extl1	exostosin-like glycosyltransferase 1 [MGI:1888742]
301	-422,996,145,149,788	Down	2.98E-10	F930017D23Rik	RIKEN cDNA F930017D23 gene [MGI:3822538]
302	-271,855,003,716,013	Down	6.27E-25	Fam117a	family with sequence similarity 117%2C member A [MGI:2144564]
303	-115,675,973,265,641	Down	4.53E+03	Fam126a	family with sequence similarity 126%2C member A [MGI:2149839]
304	-15,082,068,710,577	Down	5.82E+03	Fam214b	family with sequence similarity 214%2C member B [MGI:2441854]
305	-137,895,777,203,314	Down	4.48E+08	Fam241a	family with sequence similarity 241%2C member A [MGI:1917867]
306	-160,848,863,059,924	Down	5.11E-04	Fam53b	family with sequence similarity 53%2C member B [MGI:1925188]
307	-144,981,871,493,755	Down	0.0006788	Fancd2	Fanconi anemia%2C complementation group D2 [MGI:2448480]
308	-102,854,160,818,016	Down	1.50E+07	Fbxo30	F-box protein 30 [MGI:1919115]
309	-112,890,995,561,633	Down	3.87E+08	Fbxo34	F-box protein 34 [MGI:1926188]
310	-227,981,692,886,032	Down	7.40E-07	Fbxo5	F-box protein 5 [MGI:1914391]
311	-120,195,923,207,238	Down	0.00012	Fcho1	FCH domain only 1 [MGI:1921265]
312	-176,354,874,820,901	Down	1.56E-03	Fech	ferrochelatase [MGI:95513]
313	-242,427,834,746,361	Down	4.99E+09	Fen1	flap structure specific endonuclease 1 [MGI:102779]
314	104,435,207,503,205	Ups	0.00151	Fes	feline sarcoma oncogene [MGI:95514]
315	120,836,204,993,121	Ups	5.13E+09	Fga	fibrinogen alpha chain [MGI:1316726]
316	113,128,588,800,842	Ups	0.000111	Fgb	fibrinogen beta chain [MGI:99501]
317	125,376,899,774,325	Ups	6.92E+06	Fgl1	fibrinogen-like protein 1 [MGI:102795]
318	-444,855,038,445,132	Down	7.07E-25	Fhdc1	FH2 domain containing 1 [MGI:2684972]
319	-18,263,346,742,573	Down	6.52E+06	Fignl1	fidgetin-like 1 [MGI:1890648]
320	128,347,500,619,305	Ups	9.70E+01	Fmo5	flavin containing monooxygenase 5 [MGI:1310004]
321	-164,913,200,171,024	Down	7.11E+02	Fn3k	fructosamine 3 kinase [MGI:1926834]
322	-132,361,390,961,698	Down	0.0002300	Fn3krp	fructosamine 3 kinase related protein [MGI:2679256]
323	-172,640,964,041,203	Down	7.62E-10	Fzr1	fizzy and cell division cycle 20 related 1 [MGI:1926790]
324	-264,054,375,581,517	Down	4.68E+08	Gata1	GATA binding protein 1 [MGI:95661]
325	14,441,656,148,726	Ups	5.75E+09	Gbp5	guanylate binding protein 5 [MGI:2429943]
326	-169,615,207,974,893	Down	6.18E-09	Gch1	GTP cyclohydrolase 1 [MGI:95675]
327	256,443,720,260,061	Ups	0.0024946	Gcnt4	glucosaminyl (N-acetyl) transferase 4%2C core 2 (beta-1%2C6-N-acetylglucosaminyltransferase) [MGI:2684919]
328	-169,597,857,238,756	Down	0.0008862	Gdpd1	glycerophosphodiester phosphodiesterase domain containing 1 [MGI:1913819]
329	-150,687,423,347,316	Down	0.0002028	Gen1	GEN1%2C Holliday junction 5' flap endonuclease [MGI:2443149]
330	-238,342,196,884,754	Down	5.56E+09	Gfi1b	growth factor independent 1B [MGI:1276578]
331	-130,071,560,946,838	Down	8.75E-04	Glrx5	glutaredoxin 5 [MGI:1920296]
332	-218,970,425,899,037	Down	0.0009246	Gm10110	predicted gene 10110 [MGI:3641718]
333	612,991,770,728,039	Ups	1.76E+05	Gm10320	predicted pseudogene 10320 [MGI:3642323]
334	-34,309,053,359,124	Down	1.07E+07	Gm10371	predicted gene 10371 [MGI:3642716]
335	-267,868,358,129,519	Down	2.92E+08	Gm10603	predicted gene 10603 [MGI:3642592]
336	-256,920,023,688,061	Down	5.14E+05	Gm11427	predicted gene 11427 [MGI:3651774]
337	-312,570,981,853,823	Down	1.57E-15	Gm12346	predicted gene 12346 [MGI:3649810]
338	-11,690,620,784,272	Down	0.002523	Gm12678	predicted gene 12678 [MGI:3650923]

No	log2FoldChange	Regulation	pvalue	GeneSymbol	Description
339	186,698,049,374,709	Ups	3.83E+08	Gm12781	predicted gene 12781 [MGI:3649569]
340	-62,874,355,416,078	Down	1.21E+08	Gm14198	predicted gene 14198 [MGI:3652051]
341	-62,141,404,390,413	Down	5.20E+08	Gm15774	predicted gene 15774 [MGI:3783215]
342	-58,495,071,043,751	Down	3.05E+09	Gm15816	predicted gene 15816 [MGI:3802114]
343	-555,503,956,958,135	Down	1.08E+08	Gm16538	predicted gene 16538 [MGI:4414958]
344	-307,080,731,517,632	Down	0.003779	Gm19765	predicted gene%2C 19765 [Source:NCBI gene (formerly Entrezgene)%3BAcc:102638993]
345	-501,551,769,012,372	Down	1.49E+09	Gm20161	predicted gene%2C 20161 [MGI:5012346]
346	-188,414,836,371,948	Down	6.83E+08	Gm2788	predicted gene 2788 [MGI:3780956]
347	-520,448,835,522,959	Down	0.000114	Gm31452	predicted gene%2C 31452 [MGI:5590611]
348	-486,009,884,707,315	Down	0.00053	Gm43413	predicted gene 43413 [MGI:5663550]
349	-185,486,997,193,523	Down	0.00043	Gm43611	predicted gene 43611 [MGI:5663748]
350	-516,208,149,173,838	Down	0.000138	Gm43864	predicted gene%2C 43864 [MGI:5690256]
351	-273,508,448,785,701	Down	0.0006492	Gm44005	predicted gene%2C 44005 [MGI:5690397]
352	-116,624,469,284,803	Down	0.002815	Gm4739	predicted gene 4739 [MGI:3642984]
353	-194,665,537,845,948	Down	4.94E+09	Gm4952	predicted gene 4952 [MGI:3643569]
354	138,437,989,078,473	Ups	0.00052	Gm5182	predicted gene 5182 [MGI:3649174]
355	15,285,914,442,045	Ups	0.000239	Gm5834	predicted pseudogene 5834 [MGI:3643955]
356	-246,880,296,377,377	Down	0.002272	Gm5844	predicted gene 5844 [MGI:3645252]
357	-168,378,461,678,673	Down	7.13E+09	Gm7993	predicted gene 7993 [MGI:3647399]
358	-428,458,389,435,289	Down	1.07E-01	Gm867	predicted gene 867 [MGI:2685713]
359	-220,547,714,585,842	Down	0.000324	Gm9260	predicted gene 9260 [MGI:3645725]
360	-314,745,100,591,477	Down	6.60E+08	Gm9895	predicted gene 9895 [MGI:3642802]
361	-11,001,041,746,312	Down	0.0001920	Gmnn	geminin [MGI:1927344]
362	-120,519,016,392,804	Down	8.91E+03	Gpcpd1	glycerophosphocholine phosphodiesterase 1 [MGI:104898]
363	125,831,175,898,387	Ups	0.0002509	Gpr141	G protein-coupled receptor 141 [MGI:2672983]
364	-18,057,246,690,279	Down	9.40E+02	Gpsm2	G-protein signalling modulator 2 (AGS3-like%2C C. elegans) [MGI:1923373]
365	-197,553,063,384,887	Down	0.00381745	Gpx7	glutathione peroxidase 7 [MGI:1914555]
366	115,503,526,999,931	Ups	4.90E+08	Gvin3	GTPase%2C very large interferon inducible%2C family member 3 [MGI:3584360]
367	-548,303,720,230,752	Down	6.99E+01	Gypa	glycophorin A [MGI:95880]
368	-214,660,550,948,327	Down	7.62E-06	Gypc	glycophorin C [MGI:1098566]
369	-197,278,062,166,552	Down	4.60E+09	H1f1	H1.1 linker histone%2C cluster member [MGI:1931523]
370	-244,862,750,509,457	Down	1.13E+02	H1f3	H1.3 linker histone%2C cluster member [MGI:107502]
371	-126,187,862,308,656	Down	5.83E+05	H1f4	H1.4 linker histone%2C cluster member [MGI:1931527]
372	-182,655,285,883,368	Down	7.39E+00	H1f5	H1.5 linker histone%2C cluster member [MGI:1861461]
373	-115,268,492,197,218	Down	0.0017093	H2ac15	H2A clustered histone 15 [MGI:2448297]
374	-309,904,118,629,158	Down	1.62E+08	H2ac20	H2A clustered histone 20 [MGI:2448316]
375	-241,421,542,997,284	Down	4.70E+06	H2ac23	H2A clustered histone 23 [MGI:2448302]
376	-308,169,201,419,579	Down	1.88E+08	H2ac24	H2A clustered histone 24 [MGI:3710573]
377	-239,304,775,617,304	Down	0.00063000	H2ac4	H2A clustered histone 4 [MGI:2448306]
378	-269,819,804,184,782	Down	0.00147001	H2ac8	H2A clustered histone 8 [MGI:2448290]

No	log2FoldChange	Regulation	pvalue	GeneSymbol	Description
379	-136,746,640,718,411	Down	1.49E+01	H2ax	H2A.X variant histone [MGI:102688]
380	-195,824,652,217,105	Down	4.54E+09	H2bc13	H2B clustered histone 13 [MGI:2448403]
381	-105,080,045,759,295	Down	6.04E+03	H2bc4	H2B clustered histone 4 [MGI:1915274]
382	-429,346,412,739,036	Down	0.0004277	H2bu2	H2B.U histone 2 [MGI:1925553]
383	-152,941,744,575,309	Down	0.0011289	H3c11	H3 clustered histone 11 [MGI:2448350]
384	-234,292,817,755,685	Down	2.76E+09	H4c14	H4 clustered histone 14 [MGI:2140113]
385	-216,752,722,491,795	Down	0.002328	H4c3	H4 clustered histone 3 [MGI:2448421]
386	-148,558,794,140,707	Down	8.23E+08	H4c4	H4 clustered histone 4 [MGI:2448423]
387	-176,636,608,348,262	Down	1.52E+05	H4f16	H4 histone 16 [MGI:2448443]
388	-167,097,806,228,267	Down	3.59E+06	Hace1	HECT domain and ankyrin repeat containing%2C E3 ubiquitin protein ligase 1 [MGI:2446110]
389	144,753,415,356,626	Ups	0.0023451	Hapln4	hyaluronan and proteoglycan link protein 4 [MGI:2679531]
390	-127,170,010,632,756	Down	5.93E+09	Haus8	4HAUS augmin-like complex%2C subunit 8 [MGI:1923728]
391	-355,056,797,953,788	Down	8.68E-38	Hba-a1	hemoglobin alpha%2C adult chain 1 [MGI:96015]
392	-490,429,576,090,338	Down	2.79E+06	Hba-a2	hemoglobin alpha%2C adult chain 2 [MGI:96016]
393	-350,667,409,480,971	Down	7.38E-22	Hbb-bs	hemoglobin%2C beta adult s chain [MGI:5474852]
394	-440,627,659,169,401	Down	9.28E-51	Hbb-bt	hemoglobin%2C beta adult t chain [MGI:5474850]
395	-427,319,393,735,958	Down	2.87E-02	Hbq1b	hemoglobin%2C theta 1B [MGI:3613460]
396	-125,525,497,271,606	Down	4.00E+00	Hectd4	HECT domain E3 ubiquitin protein ligase 4 [MGI:3647820]
397	-509,724,271,334,329	Down	1.19E+09	Hemgn	hemogen [MGI:2136910]
398	-283,780,840,800,154	Down	8.38E-40	Hmbs	hydroxymethylbilane synthase [MGI:96112]
399	102,497,149,922,809	Ups	0.001251	Hmga1	high mobility group AT-hook 1 [MGI:96160]
400	-124,095,091,764,513	Down	2.28E+02	Hmgb2	high mobility group box 2 [MGI:96157]
401	-194,245,402,979,536	Down	0.0004910	Hsd3b5	hydroxy-delta-5-steroid dehydrogenase%2C 3 beta- and steroid delta-isomerase 5 [MGI:104645]
402	106,550,898,066,082	Ups	1.16E-38	Ifi204	interferon activated gene 204 [MGI:96429]
403	133,873,252,853,677	Ups	1.65E-08	Ifi206	interferon activated gene 206 [MGI:3646410]
404	116,233,763,451,878	Ups	9.46E+09	Ifi208	interferon activated gene 208 [MGI:2442822]
405	131,499,813,677,011	Ups	7.61E+01	Ifi213	interferon activated gene 213 [MGI:3695276]
406	-467,208,678,222,942	Down	0.02503	Ifi44l	interferon-induced protein 44 like [MGI:95975]
407	-352,441,339,801,377	Down	0.038499	Ifitm7	interferon induced transmembrane protein 7 [MGI:1921732]
408	-105,344,449,381,285	Down	1.55E+06	Ifrd2	interferon-related developmental regulator 2 [MGI:1316708]
409	-105,344,449,381,285	Down	2.33E+00	Ifrd2	interferon-related developmental regulator 2 [MGI:1316708]
410	106,261,099,511,468	Ups	1.70E+07	Igha	immunoglobulin heavy constant alpha [MGI:96444]
411	663,798,608,277,037	Ups	5.39E+09	Ighv1-19	immunoglobulin heavy variable V1-19 [MGI:4439779]
412	433,933,453,890,947	Ups	6.79E+09	Ighv1-22	immunoglobulin heavy variable 1-22 [MGI:4439784]
413	364,387,858,800,462	Ups	1.94E+05	Ighv1-26	immunoglobulin heavy variable 1-26 [MGI:4439641]
414	49,431,456,791,877	Ups	3.28E+09	Ighv1-39	immunoglobulin heavy variable 1-39 [MGI:4439888]
415	481,849,414,361,389	Ups	0.0004328	Ighv1-72	immunoglobulin heavy variable 1-72 [MGI:4439633]
416	277,105,638,943,079	Ups	0.0010861	Ighv9-3	immunoglobulin heavy variable V9-3 [MGI:3642720]
417	130,110,828,495,165	Ups	0.001579	Igkv1-117	immunoglobulin kappa variable 1-117 [MGI:4439721]
418	-255,507,073,473,619	Down	0.0023505	Igkv1-135	immunoglobulin kappa variable 1-135 [MGI:3819952]

No	log2FoldChange	Regulation	pvalue	GeneSymbol	Description
419	319,972,500,735,145	Ups	0.0034982	Igkv4-57	immunoglobulin kappa variable 4-57 [MGI:2685035]
420	369,553,549,681,422	Ups	5.18E+07	Igkv6-23	immunoglobulin kappa variable 6-23 [MGI:3711980]
421	11,642,690,128,155	Ups	7.26E+00	Iigp1	interferon inducible GTPase 1 [MGI:1926259]
422	290,226,373,798,985	Ups	3.27E+08	Il27	interleukin 27 [MGI:2384409]
423	-120,547,341,474,102	Down	3.80E+04	Incenp	inner centromere protein [MGI:1313288]
424	-299,399,426,802,496	Down	7.59E-30	Isg20	interferon-stimulated protein [MGI:1928895]
425	-299,399,426,802,496	Down	1.13E+06	Isg20	interferon-stimulated protein [MGI:1928895]
426	173,539,116,976,819	Ups	0.0015285	Izumo4	IZUMO family member 4 [MGI:1918814]
427	-218,074,286,186,601	Down	2.24E-03	Josd2	Josephin domain containing 2 [MGI:1913374]
428	-256,847,691,404,235	Down	2.27E-07	Kcnn4	potassium intermediate/small conductance calcium-activated channel%2C subfamily N%2C member 4 [MGI:1277957]
429	280,708,941,076,625	Ups	3.69E+06	Kcnt2	potassium channel%2C subfamily T%2C member 2 [MGI:3036273]
430	-274,681,006,876,443	Down	3.05E+09	Kctd14	potassium channel tetramerisation domain containing 14 [MGI:1289222]
431	-506,293,551,976,762	Down	1.75E-25	Kel	Kell blood group [MGI:1346053]
432	-154,627,340,092,099	Down	3.10E+00	Kif11	kinesin family member 11 [MGI:1098231]
433	-208,963,375,082,933	Down	6.17E+04	Kif14	kinesin family member 14 [MGI:1098226]
434	-151,227,891,741,848	Down	4.08E+06	Kif15	kinesin family member 15 [MGI:1098258]
435	-232,090,842,353,604	Down	2.50E+01	Kif18a	kinesin family member 18A [MGI:2446977]
436	-216,691,204,536,037	Down	4.86E+03	Kif18b	kinesin family member 18B [MGI:2446979]
437	-127,646,655,802,484	Down	0.0001016	Kif20a	kinesin family member 20A [MGI:1201682]
438	-110,570,250,665,145	Down	5.71E+08	Kif20b	kinesin family member 20B [MGI:2444576]
439	-19,988,493,378,916	Down	5.61E+00	Kif22	kinesin family member 22 [MGI:109233]
440	-131,664,045,645,185	Down	1.89E+04	Kif23	kinesin family member 23 [MGI:1919069]
441	-20,813,460,874,639	Down	1.06E+00	Kif4	kinesin family member 4 [MGI:108389]
442	-179,340,906,136,944	Down	1.96E+08	Kifc1	kinesin family member C1 [MGI:109596]
443	-373,084,278,884,926	Down	1.47E+04	Klf1	Kruppel-like factor 1 (erythroid) [MGI:1342771]
444	-149,684,739,020,354	Down	2.73E+03	Klhl12	kelch-like 12 [MGI:2385619]
445	-105,213,519,586,319	Down	6.57E+08	Klhl25	kelch-like 25 [MGI:2668031]
446	-173,958,110,653,479	Down	3.81E-05	Knl1	kinetochore scaffold 1 [MGI:1923714]
447	-20,551,740,355,432	Down	2.45E+06	Kntc1	kinetochore associated 1 [MGI:2673709]
448	-136,879,085,877,215	Down	2.78E+01	Lbr	lamin B receptor [MGI:2138281]
449	-160,689,747,517,881	Down	3.08E+09	Ldc1	leucine decarboxylase 1 [MGI:2685699]
450	-128,510,782,337,841	Down	8.15E+06	Lig1	ligase I%2C DNA%2C ATP-dependent [MGI:101789]
451	-164,299,151,159,036	Down	6.34E-02	Lmo2	LIM domain only 2 [MGI:102811]
452	-322,834,823,567,456	Down	1.91E+06	Lockd	lncRNA downstream of Cdkn1b [MGI:1915081]
453	-185,455,346,362,849	Down	1.30E-01	Lpcat1	lysophosphatidylcholine acyltransferase 1 [MGI:2384812]
454	112,292,003,085,685	Ups	1.91E+06	Manba	mannosidase%2C beta A%2C lysosomal [MGI:88175]
455	-101,393,548,617,564	Down	2.16E+07	Map4k5	mitogen-activated protein kinase kinase kinase kinase 5 [MGI:1925503]
456	-175,823,293,389,974	Down	2.98E-16	Marchf2	membrane associated ring-CH-type finger 2 [MGI:1925915]
457	-15,617,283,912,961	Down	1.11E+09	Marchf3	membrane associated ring-CH-type finger 3 [MGI:2443667]
458	-112,834,504,187,481	Down	0.0001677	Mboat2	membrane bound O-acyltransferase domain containing 2 [MGI:1914466]

No	log2FoldChange	Regulation	pvalue	GeneSymbol	Description
459	-11,968,874,738,445	Down	4.24E+09	Mcm10	minichromosome maintenance 10 replication initiation factor [MGI:1917274]
460	-147,601,275,595,337	Down	9.64E+03	Mcm2	minichromosome maintenance complex component 2 [MGI:105380]
461	-109,133,658,881,908	Down	3.61E+08	Mcm4	minichromosome maintenance complex component 4 [MGI:103199]
462	-125,144,963,044,702	Down	2.96E+06	Mcm5	minichromosome maintenance complex component 5 [MGI:103197]
463	-114,454,823,487,603	Down	9.81E+05	Mcm6	minichromosome maintenance complex component 6 [MGI:1298227]
464	-129,008,818,308,956	Down	6.67E+03	Mcm7	minichromosome maintenance complex component 7 [MGI:1298398]
465	-119,314,605,650,307	Down	5.32E+03	Memo1	mediator of cell motility 1 [MGI:1924140]
466	-103,732,668,481,372	Down	3.15E+06	Mep1b	meprin 1 beta [MGI:96964]
467	-100,083,810,263,603	Down	1.33E+06	Metap2	methionine aminopeptidase 2 [MGI:1929701]
468	-240,560,816,818,328	Down	2.11E+04	Micall2	MICAL-like 2 [MGI:2444818]
469	-18,187,559,896,186	Down	1.50E+05	Mis18bp1	MIS18 binding protein 1 [MGI:2145099]
470	-210,927,899,210,811	Down	6.50E-22	Mki67	antigen identified by monoclonal antibody Ki 67 [MGI:106035]
471	-191,912,572,083,549	Down	4.70E-06	Mkrn1	makorin%2C ring finger protein%2C 1 [MGI:1859353]
472	-198,785,608,701,946	Down	1.86E+08	Mns1	meiosis-specific nuclear structural protein 1 [MGI:107933]
473	-204,989,281,406,497	Down	3.25E+05	Mpp2	membrane protein%2C palmitoylated 2 (MAGUK p55 subfamily member 2) [MGI:1858257]
474	-117,029,237,207,137	Down	6.68E+05	Mpv17l	Mpv17 transgene%2C kidney disease mutant-like [MGI:2135951]
475	130,166,166,463,676	Ups	0.0033213	Ms4a3	membrane-spanning 4-domains%2C subfamily A%2C member 3 [MGI:2158468]
476	141,058,055,378,595	Ups	0.002245	Ms4a6d	membrane-spanning 4-domains%2C subfamily A%2C member 6D [MGI:1916024]
477	-214,895,237,321,952	Down	7.43E+08	mt-Nd3	mitochondrially encoded NADH dehydrogenase 3 [MGI:102499]
478	-329,977,657,750,103	Down	0.0001980	Mup2	major urinary protein 2 [MGI:97234]
479	-278,291,070,252,598	Down	0.0028357	Mup20	major urinary protein 20 [MGI:3651981]
480	-139,313,148,190,137	Down	2.56E+02	Mxd1	MAX dimerization protein 1 [MGI:96908]
481	-269,455,584,477,097	Down	5.62E+02	Mxd3	Max dimerization protein 3 [MGI:104987]
482	-221,545,048,475,386	Down	1.73E+02	Mybl2	myeloblastosis oncogene-like 2 [MGI:101785]
483	113,959,363,647,704	Ups	9.31E+08	Naaa	N-acylethanolamine acid amidase [MGI:1914361]
484	-120,841,621,530,531	Down	8.76E-01	Narf	nuclear prelamin A recognition factor [MGI:1914858]
485	-136,440,288,789,404	Down	5.02E+02	Ncapd2	non-SMC condensin I complex%2C subunit D2 [MGI:1915548]
486	-105,130,142,016,084	Down	2.94E+08	Ncapd3	non-SMC condensin II complex%2C subunit D3 [MGI:2142989]
487	-146,747,371,987,443	Down	3.31E+05	Ncapg	non-SMC condensin I complex%2C subunit G [MGI:1930197]
488	-213,354,993,857,359	Down	4.43E-04	Ncapg2	non-SMC condensin II complex%2C subunit G2 [MGI:1923294]
489	-143,293,754,397,778	Down	4.32E+06	Ncaph	non-SMC condensin I complex%2C subunit H [MGI:2444777]
490	-128,738,427,556,847	Down	4.04E+08	Ndc80	NDC80 kinetochore complex component [MGI:1914302]
491	-231,582,337,402,602	Down	1.73E+07	Neil3	nei like 3 (E. coli) [MGI:2384588]
492	-152,842,355,594,578	Down	0.0025070	Neu3	neuraminidase 3 [MGI:1355305]
493	-249,536,877,134,254	Down	3.44E-13	Nfe2	nuclear factor%2C erythroid derived 2 [MGI:97308]
494	-164,150,559,802,737	Down	2.68E+09	Nipa1	non imprinted in Prader-Willi/Angelman syndrome 1 homolog (human) [MGI:2442058]
495	-1,466,498,824,714	Down	1.20E+07	Nr0b2	nuclear receptor subfamily 0%2C group B%2C member 2 [MGI:1346344]
496	-109,176,036,595,858	Down	3.81E+08	Nrep	neuronal regeneration related protein [MGI:99444]
497	-244,311,901,970,211	Down	0.000375	Nrip3	nuclear receptor interacting protein 3 [MGI:1925843]
498	-15,677,379,442,245	Down	3.03E-08	Nt5c3	5'-nucleotidase%2C cytosolic III [MGI:1927186]

No	log2FoldChange	Regulation	pvalue	GeneSymbol	Description
499	-177,373,806,663,249	Down	0.000462	Nudt7	nudix (nucleoside diphosphate linked moiety X)-type motif 7 [MGI:1914778]
500	-155,431,906,414,549	Down	3.60E+08	Nuf2	NUF2%2C NDC80 kinetochore complex component [MGI:1914227]
501	-198,319,508,927,985	Down	1.09E-06	Nusap1	nucleolar and spindle associated protein 1 [MGI:2675669]
502	-28,398,293,565,817	Down	1.68E+03	Nxpe2	neurexophilin and PC-esterase domain family%2C member 2 [MGI:1925502]
503	-127,215,976,099,217	Down	0.000797	Nxpe4	neurexophilin and PC-esterase domain family%2C member 4 [MGI:1924792]
504	-238,320,275,085,706	Down	5.60E+09	Odc1	ornithine decarboxylase%2C structural 1 [MGI:97402]
505	-165,553,659,977,282	Down	0.0004733	Oip5	Opa interacting protein 5 [MGI:1917895]
506	108,096,684,549,679	Ups	0.0024892	Olfm3	olfactomedin 3 [MGI:2387329]
507	-283,257,720,925,897	Down	2.21E+06	Orc1	origin recognition complex%2C subunit 1 [MGI:1328337]
508	-144,347,372,912,144	Down	2.70E+09	Orc6	origin recognition complex%2C subunit 6 [MGI:1929285]
509	35,599,788,502,309	Ups	0.0001412	Orm3	orosomucoid 3 [MGI:97445]
510	-160,476,670,113,121	Down	8.02E+06	Otub2	OTU domain%2C ubiquitin aldehyde binding 2 [MGI:1915399]
511	-126,804,699,645,487	Down	3.82E+02	Pabpc4	poly(A) binding protein%2C cytoplasmic 4 [MGI:2385206]
512	-163,782,650,183,335	Down	1.38E+07	Pbk	PDZ binding kinase [MGI:1289156]
513	-112,480,043,749,025	Down	1.14E+06	Pck2	phosphoenolpyruvate carboxykinase 2 (mitochondrial) [MGI:1860456]
514	-154,006,288,151,557	Down	6.26E-02	Pcna	proliferating cell nuclear antigen [MGI:97503]
515	-236,313,007,641,399	Down	3.01E+09	Pcyt1b	phosphate cytidylyltransferase 1%2C choline%2C beta isoform [MGI:2147987]
516	-268,000,622,927,342	Down	1.27E+06	Pheta2	PH domain containing endocytic trafficking adaptor 2 [MGI:2443609]
517	-23,417,394,832,979	Down	6.83E+09	Phyhip	phytanoyl-CoA hydroxylase interacting protein [MGI:1860417]
518	-199,852,186,382,617	Down	0.0006105	Pif1	PIF1 5'-to-3' DNA helicase [MGI:2143057]
519	-114,391,678,915,603	Down	6.06E+09	Piga	phosphatidylinositol glycan anchor biosynthesis%2C class A [MGI:99461]
520	-165,862,956,493,817	Down	9.08E+08	Pip5k1b	phosphatidylinositol-4-phosphate 5-kinase%2C type 1 beta [MGI:107930]
521	-422,190,191,000,692	Down	1.17E-04	Pkhd1l1	polycystic kidney and hepatic disease 1-like 1 [MGI:2183153]
522	-133,554,728,906,919	Down	0.0016432	Pkmyt1	protein kinase%2C membrane associated tyrosine/threonine 1 [MGI:2137630]
523	-103,021,451,304,107	Down	5.56E+08	Plaat3	phospholipase A and acyltransferase 3 [MGI:2179715]
524	-121,841,781,188,322	Down	8.07E+06	Plcl2	phospholipase C-like 2 [MGI:1352756]
525	-137,785,440,108,057	Down	0.0003704	Plek2	pleckstrin 2 [MGI:1351466]
526	-51,058,690,483,613	Down	3.97E+09	Pnp2	purine-nucleoside phosphorylase 2 [MGI:3712328]
527	-103,722,822,186,076	Down	0.002401	Pole	polymerase (DNA directed)%2C epsilon [MGI:1196391]
528	-180,747,496,266,943	Down	1.33E+09	Polq	polymerase (DNA directed)%2C theta [MGI:2155399]
529	-518,918,573,405,362	Down	0.000159	Popdc2	popeye domain containing 2 [MGI:1930150]
530	-169,357,880,429,875	Down	1.78E+02	Ppox	protoporphyrinogen oxidase [MGI:104968]
531	-274,613,209,562,544	Down	4.82E-27	Ppp1r15a	protein phosphatase 1%2C regulatory subunit 15A [MGI:1927072]
532	164,062,400,649,655	Ups	1.81E-01	Ppp1r3b	protein phosphatase 1%2C regulatory subunit 3B [MGI:2177268]
533	13,069,898,470,993	Ups	0.0030755	Ppp1r3c	protein phosphatase 1%2C regulatory subunit 3C [MGI:1858229]
534	-199,819,512,800,083	Down	1.14E+00	Prc1	protein regulator of cytokinesis 1 [MGI:1858961]
535	-190,791,695,692,574	Down	3.80E-09	Prdx2	peroxiredoxin 2 [MGI:109486]
536	-142,152,055,452,488	Down	1.71E+09	Prim2	DNA primase%2C p58 subunit [MGI:97758]
537	-113,546,280,622,297	Down	1.21E+07	Prkar2b	protein kinase%2C cAMP dependent regulatory%2C type II beta [MGI:97760]
538	136,953,453,811,802	Ups	0.0007313	Prss34	protease%2C serine 34 [MGI:2681414]

No	log2FoldChange	Regulation	pvalue	GeneSymbol	Description
539	-113,258,492,297,175	Down	1.09E+04	Ptdss2	phosphatidylserine synthase 2 [MGI:1351664]
540	-143,612,542,340,299	Down	2.87E-05	Ptp4a3	protein tyrosine phosphatase 4a3 [MGI:1277098]
541	-19,380,923,233,513	Down	2.93E-13	Rab3il1	RAB3A interacting protein (rabin3)-like 1 [MGI:1922010]
542	-190,842,912,053,619	Down	4.75E-20	Rad23a	RAD23 homolog A%2C nucleotide excision repair protein [MGI:105126]
543	-157,880,208,250,406	Down	1.06E+07	Rad51	RAD51 recombinase [MGI:97890]
544	-142,348,721,712,068	Down	0.0008486	Rad51ap1	RAD51 associated protein 1 [MGI:1098224]
545	-15,294,397,934,353	Down	0.0002489	Rad54l	RAD54 like (S. cerevisiae) [MGI:894697]
546	-187,055,461,686,305	Down	1.09E-22	Ranbp10	RAN binding protein 10 [MGI:1921584]
547	-112,198,673,482,816	Down	8.21E+08	Rarres1	retinoic acid receptor responder (tazarotene induced) 1 [MGI:1924461]
548	-229,015,300,712,318	Down	4.13E-19	Rbm38	RNA binding motif protein 38 [MGI:1889294]
549	-241,266,364,742,726	Down	0.0030583	Recql4	RecQ protein-like 4 [MGI:1931028]
550	-113,331,029,232,726	Down	0.0001828	Reep4	receptor accessory protein 4 [MGI:1919799]
551	-943,823,832,888,101	Down	0.000741	Reg3a	regenerating islet-derived 3 alpha [MGI:109408]
552	-118,953,511,401,736	Down	0.003701	Retnlg	resistin like gamma [MGI:2667763]
553	-130,872,795,112,319	Down	1.19E+08	Rfc4	replication factor C (activator 1) 4 [MGI:2146571]
554	-217,789,024,843,323	Down	8.74E+04	Rfx2	regulatory factor X%2C 2 (influences HLA class II expression) [MGI:106583]
555	-115,211,431,863,784	Down	7.14E+09	Rgs12	regulator of G-protein signaling 12 [MGI:1918979]
556	-410,506,600,678,075	Down	1.47E+05	Rhag	Rhesus blood group-associated A glycoprotein [MGI:1202713]
557	-54,182,571,062,468	Down	1.29E+05	Rhd	Rh blood group%2C D antigen [MGI:1202882]
558	-110,444,912,259,562	Down	2.23E+04	Rnf123	ring finger protein 123 [MGI:2148796]
559	-380,686,254,609,759	Down	5.13E+09	Rnf212	ring finger protein 212 [MGI:3645767]
560	-144,000,192,023,664	Down	4.71E+06	Rnf24	ring finger protein 24 [MGI:1261771]
561	-173,222,591,805,489	Down	0.0025895	Rpl10a-ps4	ribosomal protein L10A%2C pseudogene 4 [MGI:3652221]
562	348,393,704,895,491	Ups	3.07E+02	Rps24-ps3	ribosomal protein S24%2C pseudogene 3 [MGI:3644407]
563	-611,133,982,388,721	Down	2.25E-10	Rps3a3	ribosomal protein S3A3 [MGI:3643406]
564	-156,241,340,525,208	Down	3.68E+00	Rrm1	ribonucleotide reductase M1 [MGI:98180]
565	-255,767,937,548,605	Down	2.65E-21	Rrm2	ribonucleotide reductase M2 [MGI:98181]
566	-316,912,043,287,825	Down	1.15E-67	Rsad2	radical S-adenosyl methionine domain containing 2 [MGI:1929628]
567	305,974,693,616,435	Ups	7.04E+09	Saa2	serum amyloid A 2 [MGI:98222]
568	222,139,048,360,841	Ups	3.93E+09	Saa3	serum amyloid A 3 [MGI:98223]
569	-117,141,628,631,457	Down	4.45E+06	Samd1	sterile alpha motif domain containing 1 [MGI:2142433]
570	-436,637,884,958,386	Down	3.48E+00	Samd11	sterile alpha motif domain containing 11 [MGI:2446220]
571	-184,040,821,773,928	Down	8.38E+02	Samd14	sterile alpha motif domain containing 14 [MGI:2384945]
572	204,717,866,052,733	Ups	0.003733	Serpina3c	serine (or cysteine) peptidase inhibitor%2C clade A%2C member 3C [MGI:102848]
573	110,097,643,081,289	Ups	7.37E+09	Serpina3n	serine (or cysteine) peptidase inhibitor%2C clade A%2C member 3N [MGI:105045]
574	-243,050,063,964,413	Down	2.64E+04	Sgo1	shugoshin 1 [MGI:1919665]
575	-102,113,878,178,909	Down	0.000503	Siva1	SIVA1%2C apoptosis-inducing factor [MGI:1353606]
576	-241,908,068,597,165	Down	1.17E+06	Ska1	spindle and kinetochore associated complex subunit 1 [MGI:1913718]
577	-114,794,150,524,665	Down	0.0020226	Ska2	spindle and kinetochore associated complex subunit 2 [MGI:1913390]
578	-149,852,445,394,316	Down	0.0015839	Ska3	spindle and kinetochore associated complex subunit 3 [MGI:3041235]

No	log2FoldChange	Regulation	pvalue	GeneSymbol	Description
579	-126,875,865,040,769	Down	1.84E+05	Slbp	stem-loop binding protein [MGI:108402]
580	-168,460,793,554,664	Down	3.84E+01	Slc14a1	solute carrier family 14 (urea transporter)%2C member 1 [MGI:1351654]
581	-14,098,009,920,707	Down	1.07E-04	Slc16a1	solute carrier family 16 (monocarboxylic acid transporters)%2C member 1 [MGI:106013]
582	-13,887,970,115,991	Down	6.95E+07	Slc16a6	solute carrier family 16 (monocarboxylic acid transporters)%2C member 6 [MGI:2144585]
583	-106,332,182,050,856	Down	0.000506	Slc25a21	solute carrier family 25 (mitochondrial oxodicarboxylate carrier)%2C member 21 [MGI:2445059]
584	-372,338,424,295,826	Down	1.07E-82	Slc25a37	solute carrier family 25%2C member 37 [MGI:1914962]
585	-163,953,030,999,418	Down	7.84E+00	Slc25a38	solute carrier family 25%2C member 38 [MGI:2384782]
586	-359,307,856,820,929	Down	1.84E-18	Slc38a5	solute carrier family 38%2C member 5 [MGI:2148066]
587	-325,621,839,510,778	Down	1.06E-36	Slc43a1	solute carrier family 43%2C member 1 [MGI:1931352]
588	-478,286,732,117,893	Down	8.27E-51	Slc4a1	solute carrier family 4 (anion exchanger)%2C member 1 [MGI:109393]
589	-130,715,145,483,562	Down	1.25E+07	Slc6a9	solute carrier family 6 (neurotransmitter transporter%2C glycine)%2C member 9 [MGI:95760]
590	-374,182,960,414,886	Down	3.35E-11	Slfn14	schlafen 14 [MGI:2684866]
591	14,243,290,941,868	Ups	0.002319	Slfn4	schlafen 4 [MGI:1329010]
592	-152,194,653,314,024	Down	5.01E+03	Smc2	structural maintenance of chromosomes 2 [MGI:106067]
593	-120,944,694,952,447	Down	2.57E-01	Smc4	structural maintenance of chromosomes 4 [MGI:1917349]
594	-282,458,031,163,433	Down	4.76E-12	Smox	spermine oxidase [MGI:2445356]
595	-331,359,775,148,167	Down	4.01E-36	Snca	synuclein%2C alpha [MGI:1277151]
596	-135,126,865,118,837	Down	1.57E+06	Snrnp25	small nuclear ribonucleoprotein 25 (U11/U12) [MGI:1925622]
597	-116,319,878,051,147	Down	7.33E+08	Snx15	sorting nexin 15 [MGI:1916274]
598	-343,718,365,533,452	Down	4.51E+01	Snx22	sorting nexin 22 [MGI:2685966]
599	-36,273,862,864,062	Down	9.60E-06	Sowaha	sosondowah ankyrin repeat domain family member A [MGI:2687280]
600	-148,464,388,852,889	Down	3.42E-04	Sox6	SRY (sex determining region Y)-box 6 [MGI:98368]
601	-20,064,500,985,242	Down	4.21E+04	Spag5	sperm associated antigen 5 [MGI:1927470]
602	-196,899,107,604,472	Down	1.04E-12	Specc1	sperm antigen with calponin homology and coiled-coil domains 1 [MGI:2442356]
603	-115,803,984,906,765	Down	0.001207	Sphk1	sphingosine kinase 1 [MGI:1316649]
604	-150,636,081,552,833	Down	4.58E-02	Spire1	spire type actin nucleation factor 1 [MGI:1915416]
605	-120,181,964,832,544	Down	4.07E+06	Sppl2b	signal peptide peptidase like 2B [MGI:1920468]
606	-438,544,262,390,164	Down	5.76E-07	Spta1	spectrin alpha%2C erythrocytic 1 [MGI:98385]
607	-392,062,295,801,732	Down	2.14E-04	Sptb	spectrin beta%2C erythrocytic [MGI:98387]
608	115,580,697,839,885	Ups	1.94E+08	Steap4	STEAP family member 4 [MGI:1923560]
609	-161,969,704,208,448	Down	5.23E+09	Stil	Scl/Tal1 interrupting locus [MGI:107477]
610	-19,106,387,774,254	Down	1.41E-07	Stom	stomatin [MGI:95403]
611	230,571,066,005,568	Ups	4.01E+07	Stra6	stimulated by retinoic acid gene 6 [MGI:107742]
612	-141,004,656,326,803	Down	8.64E+03	Stx2	syntaxin 2 [MGI:108059]
613	-131,851,731,315,034	Down	0.0011666	Sult2a8	sulfotransferase family 2A%2C dehydroepiandrosterone (DHEA)-preferring%2C member 8 [MGI:1924221]
614	-261,931,832,272,963	Down	0.0007953	Sult5a1	sulfotransferase family 5A%2C member 1 [MGI:1931463]
615	-14,190,324,205,154	Down	2.46E+07	Tacc3	transforming%2C acidic coiled-coil containing protein 3 [MGI:1341163]
616	503,623,170,106,161	Ups	0.0002119	Tafa2	TAFA chemokine like family member 2 [MGI:2143691]
617	-325,086,069,145,495	Down	8.62E+05	Tal1	T cell acute lymphocytic leukemia 1 [MGI:98480]
618	-210,995,098,023,278	Down	0.0002529	Tbx10	T-box 10 [MGI:1261436]

No	log2FoldChange	Regulation	pvalue	GeneSymbol	Description
619	-159,471,333,866,159	Down	6.68E+07	Tcf19	transcription factor 19 [MGI:103180]
620	-248,896,660,669,509	Down	1.68E-30	Tent5c	terminal nucleotidyltransferase 5C [MGI:1921895]
621	-170,642,849,940,041	Down	6.83E-11	Tfdp2	transcription factor Dp 2 [MGI:107167]
622	-358,566,486,448,122	Down	1.56E+06	Tff3	trefoil factor 3%2C intestinal [MGI:104638]
623	-230,606,655,703,201	Down	0.000300	Tiaf2	TGF-beta1-induced anti-apoptotic factor 2 [MGI:2651383]
624	121,678,929,611,201	Ups	8.70E+05	Ticam2	toll-like receptor adaptor molecule 2 [MGI:3040056]
625	-17,466,672,340,227	Down	4.26E+08	Ticrr	TOPBP1-interacting checkpoint and replication regulator [MGI:1924261]
626	143,704,416,113,953	Ups	5.38E+09	Tifab	TRAF-interacting protein with forkhead-associated domain%2C family member B [MGI:2385852]
627	-118,774,787,973,333	Down	2.33E+08	Tipin	timeless interacting protein [MGI:1921571]
628	-133,281,424,818,044	Down	4.29E+05	Tk1	thymidine kinase 1 [MGI:98763]
629	-136,345,896,551,049	Down	1.09E+07	Tlcd2	TLC domain containing 2 [MGI:1917141]
630	-465,699,628,759,296	Down	9.31E-90	Tmcc2	transmembrane and coiled-coil domains 2 [MGI:1916125]
631	-192,960,105,500,944	Down	1.15E+08	Tmem120b	transmembrane protein 120B [MGI:3603158]
632	-411,430,459,234,418	Down	5.80E-09	Tmod1	tropomodulin 1 [MGI:98775]
633	148,687,301,380,935	Ups	1.67E+08	Tnfaip8l3	tumor necrosis factor%2C alpha-induced protein 8-like 3 [MGI:2685363]
634	169,436,520,457,026	Ups	0.016550	Tnip3	TNFAIP3 interacting protein 3 [MGI:3041165]
635	-138,388,896,993,875	Down	5.80E-02	Top2a	topoisomerase (DNA) II alpha [MGI:98790]
636	-169,690,657,396,511	Down	2.47E-01	Tpx2	TPX2%2C microtubule-associated [MGI:1919369]
637	-126,434,600,160,647	Down	2.70E-06	Trak2	trafficking protein%2C kinesin binding 2 [MGI:1918077]
638	-576,628,164,768,095	Down	2.12E-04	Trim10	tripartite motif-containing 10 [MGI:1338757]
639	-42,834,272,518,085	Down	1.02E-03	Trim58	tripartite motif-containing 58 [MGI:2684862]
640	-221,169,706,295,654	Down	1.84E-13	Trim59	tripartite motif-containing 59 [MGI:1914199]
641	-126,651,330,473,817	Down	0.001919	Trmo	tRNA methyltransferase O [MGI:1922003]
642	-237,957,976,739,466	Down	0.0007837	Troap	trophinin associated protein [MGI:1925983]
643	-220,821,382,406,845	Down	5.00E-13	Tspan33	tetraspanin 33 [MGI:1919012]
644	-376,237,395,848,837	Down	1.39E+03	Tspo2	translocator protein 2 [MGI:1917276]
645	-187,698,275,323,523	Down	0.00018	Ttk	Ttk protein kinase [MGI:1194921]
646	-112,612,864,708,692	Down	3.69E+06	Txnrd2	thioredoxin reductase 2 [MGI:1347023]
647	-148,908,660,646,242	Down	2.41E+06	Tyms	thymidylate synthase [MGI:98878]
648	-118,163,444,196,841	Down	0.00015	Tyw3	tRNA-yW synthesizing protein 3 homolog (S. cerevisiae) [MGI:2445040]
649	-215,276,792,522,191	Down	2.20E-14	Ubac1	ubiquitin associated domain containing 1 [MGI:1920995]
650	-246,261,782,626,932	Down	8.75E-30	Ube2c	ubiquitin-conjugating enzyme E2C [MGI:1915862]
651	-159,084,424,542,164	Down	0.0005830	Ube2l6	ubiquitin-conjugating enzyme E2L 6 [MGI:1914500]
652	-238,547,078,039,476	Down	2.30E-22	Ube2o	ubiquitin-conjugating enzyme E2O [MGI:2444266]
653	-116,147,202,945,734	Down	1.81E+05	Ube2s	ubiquitin-conjugating enzyme E2S [MGI:1925141]
654	-130,875,317,488,035	Down	1.64E-01	Ucp2	uncoupling protein 2 (mitochondrial%2C proton carrier) [MGI:109354]
655	-174,727,432,181,203	Down	2.26E-03	Uhrf1	ubiquitin-like%2C containing PHD and RING finger domains%2C 1 [MGI:1338889]
656	-139,266,542,376,965	Down	4.17E-06	Urod	uroporphyrinogen decarboxylase [MGI:98916]
657	-265,529,372,240,203	Down	7.31E-15	Uros	uroporphyrinogen III synthase [MGI:98917]
658	-184,979,512,375,868	Down	9.17E-02	Vopp1	vesicular%2C overexpressed in cancer%2C prosurvival protein 1 [MGI:2141658]

No	log2FoldChange	Regulation	pvalue	GeneSymbol	Description
659	-185,043,083,843,207	Down	2.15E+04	Wdhd1	WD repeat and HMG-box DNA binding protein 1 [MGI:2443514]
660	-174,863,785,236,771	Down	2.31E+06	Xk	X-linked Kx blood group [MGI:103569]
661	-17,940,655,608,949	Down	2.62E-16	Xpo7	exportin 7 [MGI:1929705]
662	-796,568,528,538,149	Down	4.51E+03	Ypel4	yippee like 4 [MGI:3605071]
663	122,983,874,261,716	Ups	0.00011	Zbtb16	zinc finger and BTB domain containing 16 [MGI:103222]
664	-121,754,966,844,225	Down	7.84E+07	Zfp367	zinc finger protein 367 [MGI:2442266]
665	-198,505,344,246,177	Down	1.23E-08	Zfpm1	zinc finger protein%2C multitype 1 [MGI:1095400]

Supplementary Table S2B

No	GeneSymbol	log2FoldChange	Regulation	pvalue	Description
1	Arrb1	-126,682,184,625,746	Down	3.85E+09	arrestin%2C beta 1 [MGI:99473]
2	Ccr8	-249,257,209,704,091	Down	0.00541197569589675	chemokine (C-C motif) receptor 8 [MGI:1201402]
3	Ccl1	-224,763,623,519,066	Down	0.0161466172851243	chemokine (C-C motif) ligand 1 [MGI:98258]
4	Ccl7	-166,827,998,525,313	Down	1.03E+08	chemokine (C-C motif) ligand 7 [MGI:99512]
5	Cxcl5	-112,385,380,828,418	Down	0.0361351883780983	chemokine (C-X-C motif) ligand 5 [MGI:1096868]
6	Ccr1l1	-102,916,558,903,242	Down	4.70E+09	chemokine (C-C motif) receptor 1-like 1 [MGI:104617]
7	Ccl22	-100,925,309,458,525	Down	0.00190796071483424	chemokine (C-C motif) ligand 22 [MGI:1306779]
8	Ccr5	101,506,248,042,418	Ups	0.00912425126402945	chemokine (C-C motif) receptor 5 [MGI:107182]
9	Cxcl10	130,353,423,350,035	Ups	1.17E+04	chemokine (C-X-C motif) ligand 10 [MGI:1352450]
10	Cxcl11	134,485,427,847,557	Ups	7.78E+03	chemokine (C-X-C motif) ligand 11 [MGI:1860203]
11	Cxcl3	169,873,919,381,099	Ups	0.0240709820180269	chemokine (C-X-C motif) ligand 3 [MGI:3037818]
12	Ccl28	183,296,823,489,948	Ups	0.00087067527861451	chemokine (C-C motif) ligand 28 [MGI:1861731]
13	Ccl3	208,633,154,195,104	Ups	1.06E+07	chemokine (C-C motif) ligand 3 [MGI:98260]
14	Cxcl17	212,172,944,315,682	Ups	1.97E+08	chemokine (C-X-C motif) ligand 17 [MGI:2387642]
15	Cxcl9	298,151,415,515,268	Ups	2.79E-47	chemokine (C-X-C motif) ligand 9 [MGI:1352449]
16	Ccl8	357,811,639,287,199	Ups	4.50E+00	chemokine (C-C motif) ligand 8 [MGI:101878]
17	Cd163	118,992,373,626,094	Ups	7.29E+05	CD163 antigen [MGI:2135946]
18	Cd1d2	189,534,233,475,896	Ups	0.0341798732138818	CD1d2 antigen [MGI:107675]
19	Cd200r2	173,987,013,393,556	Ups	4.41E+06	Cd200 receptor 2 [MGI:3042847]
20	Cd200r4	164,226,301,098,143	Ups	0.0123288404931076	CD200 receptor 4 [MGI:3036289]
21	Cd207	278,228,146,955,687	Ups	3.58E-18	CD207 antigen [MGI:2180021]
22	Cd209a	-115,736,055,826,259	Down	0.000112962857041857	CD209a antigen [MGI:2157942]
23	Cd209f	-234,546,065,964,854	Down	1.60E-06	CD209f antigen [MGI:1916392]
24	Cd24a	-206,637,804,322,302	Down	2.53E+09	CD24a antigen [MGI:88323]
25	Cd24a	-206,637,804,322,302	Down	0.00481493938423419	CD24a antigen [MGI:88323]
26	Cd274	125,903,451,185,579	Ups	0.0474080845076971	CD274 antigen [MGI:1926446]

No	GeneSymbol	log2FoldChange	Regulation	pvalue	Description
27	Cd300c	141,814,574,091,256	Ups	0.0102459369324697	CD300C molecule [MGI:3032626]
28	Cd300e	129,037,636,815,681	Ups	1.66E-03	CD300E molecule [MGI:2387602]
29	Cd300lb	110,785,446,487,232	Ups	0.0335544764585564	CD300 molecule like family member B [MGI:2685099]
30	Cd300ld	146,838,087,156,572	Ups	0.00269449651645808	CD300 molecule like family member d [MGI:2442358]
31	Cd300ld2	-102,916,558,903,242	Down	0.000190917126226982	CD300 molecule like family member D2 [MGI:3649405]
32	Cd300ld3	-245,102,221,764,792	Down	2.16E-07	CD300 molecule like family member D3 [MGI:2687214]
33	Cd46	261,718,379,132,325	Ups	1.56E+08	CD46 antigen%2C complement regulatory protein [MGI:1203290]
34	Cd55b	-102,916,558,903,242	Down	0.000948545222144646	CD55 molecule%2C decay accelerating factor for complement B [MGI:104849]
35	Cd69	105,306,531,517,425	Ups	0.00144538210611988	CD69 antigen [MGI:88343]
36	Cdc25b	-194,937,025,723,248	Down	1.31E+09	cell division cycle 25B [MGI:99701]
37	Ifi202b	154,567,207,683,445	Ups	0.0269931999868927	interferon activated gene 202B [MGI:1347083]
38	Ifi203-ps	132,684,938,869,629	Ups	3.72E+07	interferon activated gene 203%2C pseudogene [MGI:3840117]
39	Ifi204	174,234,517,155,698	Ups	0.00901579846684296	interferon activated gene 204 [MGI:96429]
40	Ifi205	113,653,847,374,474	Ups	1.53E+01	interferon activated gene 205 [MGI:101847]
41	Ifi206	124,368,945,457,769	Ups	0.049835850714925	interferon activated gene 206 [MGI:3646410]
42	Ifi207	110,084,160,991,092	Ups	0.0347682995950831	interferon activated gene 207 [MGI:2138302]
43	Ifi211	228,570,129,943,568	Ups	0.0343555049888864	interferon activated gene 211 [MGI:3041120]
44	Ifi213	113,762,277,569,128	Ups	0.00720094925434131	interferon activated gene 213 [MGI:3695276]
45	Ifi27l2b	172,226,380,355,558	Ups	0.00134672923325406	interferon%2C alpha-inducible protein 27 like 2B [MGI:1916390]
46	Ifi44	123,003,622,906,065	Ups	0.00579863342357507	interferon-induced protein 44 [MGI:2443016]
47	Ifi44l	-484,050,411,850,084	Down	3.41E+06	interferon-induced protein 44 like [MGI:95975]
48	Ifit2	12,612,286,259,366	Ups	0.0106584206143329	interferon-induced protein with tetratricopeptide repeats 2 [MGI:99449]
49	Ifit3	162,697,947,386,414	Ups	0.000257312127273739	interferon-induced protein with tetratricopeptide repeats 3 [MGI:1101055]
50	Ifit3	162,697,947,386,414	Ups	7.57E+09	interferon-induced protein with tetratricopeptide repeats 3 [MGI:1101055]
51	Ifit3b	114,372,273,423,023	Ups	0.000236956507679813	interferon-induced protein with tetratricopeptide repeats 3B [MGI:3698419]
52	Ifitm5	-242,152,647,283,567	Down	0.000174473229762461	interferon induced transmembrane protein 5 [MGI:1934923]
53	Ifitm7	127,863,067,845,322	Ups	0.000180657104134611	interferon induced transmembrane protein 7 [MGI:1921732]
54	Ifng	346,349,453,681,797	Ups	0.00205732852765172	interferon gamma [MGI:107656]
55	Iglc1	-207,555,589,316,475	Down	5.38E+08	immunoglobulin lambda constant 1 [MGI:99546]
56	Il11	245,294,076,014,196	Ups	0.00168515114195359	interleukin 11 [MGI:107613]
57	Il12rb1	107,459,431,393,926	Ups	0.0106343630983416	interleukin 12 receptor%2C beta 1 [MGI:104579]
58	Il13ra2	-115,230,703,194,707	Down	3.35E+07	interleukin 13 receptor%2C alpha 2 [MGI:1277954]
59	Il17c	246,132,411,893,887	Ups	1.31E-34	interleukin 17C [MGI:2446486]
60	Il17f	103,989,380,545,395	Ups	0.0393350272109223	interleukin 17F [MGI:2676631]
61	Il18bp	182,819,544,535,121	Ups	0.0193064993864519	interleukin 18 binding protein [MGI:1333800]
62	Il1b	12,103,177,756,933	Ups	0.00297030828312564	interleukin 1 beta [MGI:96543]
63	Il1bos	-102,916,558,903,242	Down	0.0494950401221928	interleukin 1 beta%2C opposite strand [MGI:3650458]
64	Il1rl1	-175,102,360,589,306	Down	0.0166372331393263	interleukin 1 receptor-like 1 [MGI:98427]
65	Il1rn	155,307,427,802,513	Ups	6.72E+09	interleukin 1 receptor antagonist [MGI:96547]
66	Il20rb	-104,987,544,153,665	Down	5.15E+09	interleukin 20 receptor beta [MGI:2143266]

No	GeneSymbol	log2FoldChange	Regulation	pvalue	Description
67	Il21	-180,585,568,839,724	Down	1.61E-01	interleukin 21 [MGI:1890474]
68	Il22ra1	117,660,927,200,449	Ups	0.00458709777878779	interleukin 22 receptor%2C alpha 1 [MGI:2663588]
69	Il22ra2	136,722,159,334,693	Ups	0.000114223794106449	interleukin 22 receptor%2C alpha 2 [MGI:2665114]
70	Il24	-102,916,558,903,242	Down	0.000492740545165748	interleukin 24 [MGI:2135548]
71	Il31ra	169,873,919,381,099	Ups	1.68E+03	interleukin 31 receptor A [MGI:2180511]
72	Il36rn	-107,151,736,343,667	Down	0.00116538584440261	interleukin 36 receptor antagonist [MGI:1859325]
73	Il4	-122,610,758,200,174	Down	0.000366298038534149	interleukin 4 [MGI:96556]
74	Il9r	-203,511,687,385,823	Down	2.41E+09	interleukin 9 receptor [MGI:96564]
75	Irf7	102,038,181,523,038	Ups	0.00348237516559394	interferon regulatory factor 7 [MGI:1859212]
76	Isg20	-182,251,684,952,779	Down	0.00160010702552367	interferon-stimulated protein [MGI:1928895]
77	Stmn1	-11,484,271,190,037	Down	0.000243917427893787	stathmin 1 [MGI:96739]
78	Tfrc	-175,894,654,770,922	Down	0.000146188260043436	transferrin receptor [MGI:98822]
79	Tgif2-ps2	-275,226,346,620,121	Down	1.01E+04	TGFB-induced factor homeobox 2%2C pseudogene 2 [MGI:3805950]
80	Tlr6	128,859,176,462,586	Ups	7.51E+03	toll-like receptor 6 [MGI:1341296]
81	Tlr8	123,215,009,396,601	Ups	8.73E+04	toll-like receptor 8 [MGI:2176887]
82	Tnfa	103,259,031,945,545	Ups	0.0126044106243899	tumor necrosis factor [MGI:104798]
83	Tnfrsf11a	110,203,941,880,374	Ups	0.0056012613460807	tumor necrosis factor receptor superfamily%2C member 11a%2C NFKB activator [MGI:1314891]
84	Tnfrsf18	-113,450,306,048,663	Down	6.24E-13	tumor necrosis factor receptor superfamily%2C member 18 [MGI:894675]
85	Tnfsf11	-226,140,185,009,885	Down	0.035959516083427	tumor necrosis factor (ligand) superfamily%2C member 11 [MGI:1100089]
86	Tnfsf15	129,789,562,497,796	Ups	0.00135939643248276	tumor necrosis factor (ligand) superfamily%2C member 15 [MGI:2180140]
87	Tnfsf18	-259,266,617,951,788	Down	6.77E+04	tumor necrosis factor (ligand) superfamily%2C member 18 [MGI:2673064]
88	Tnfsfm13	153,967,727,082,311	Ups	0.00140840138193554	tumor necrosis factor (ligand) superfamily%2C membrane-bound member 13 [MGI:3845075]
89	Tnfsf13os	196,705,766,442,873	Ups	0.0002905955302781	tumor necrosis factor (ligand) superfamily%2C member 13%2C opposite strand [MGI:1919587]
90	Abcb10	-151,158,669,656,812	Down	0.000197939373804585	ATP-binding cassette%2C sub-family B (MDR/TAP)%2C member 10 [MGI:1860508]
91	Alas2	-255,908,950,261,728	Down	4.78E+08	aminolevulinic acid synthase 2%2C erythroid [MGI:87990]
92	Asb1	-165,676,744,053,143	Down	5.14E+07	ankyrin repeat and SOCS box-containing 1 [MGI:1929735]
93	Banp	-131,750,890,870,626	Down	0.000163539227756584	BTG3 associated nuclear protein [MGI:1889023]
94	Bpgm	-181,507,237,334,295	Down	1.63E-02	2%2C3-bisphosphoglycerate mutase [MGI:1098242]
95	Brca1	-199,567,149,808,251	Down	0.00032951834485325	breast cancer 1%2C early onset [MGI:104537]
96	Brpf3	-148,923,393,954,232	Down	1.34E+09	bromodomain and PHD finger containing%2C 3 [MGI:2146836]
97	Cdr2	-108,936,006,968,375	Down	0.00019639460475687	cerebellar degeneration-related 2 [MGI:1100885]
98	Ceacam2	184,383,470,257,881	Ups	1.74E+07	carcinoembryonic antigen-related cell adhesion molecule 2 [MGI:1347246]
99	Cfd	146,801,400,458,385	Ups	0.000112130960120849	complement factor D (adipsin) [MGI:87931]
100	Chac2	-100,973,559,023,206	Down	0.000199635055492286	ChaC%2C cation transport regulator 2 [MGI:1915294]
101	Cit	-123,044,873,821,842	Down	0.000280086616871463	citron [MGI:105313]
102	Cmas	-109,839,778,757,217	Down	3.40E+08	cytidine monophospho-N-acetylneuraminic acid synthetase [MGI:1337124]
103	Cmklr1	112,060,460,534,972	Ups	0.00197449987609723	chemokine-like receptor 1 [MGI:109603]
104	Cracd	-188,841,277,213,941	Down	2.95E+09	capping protein inhibiting regulator of actin [MGI:2444817]
105	Cyp39a1	209,690,143,373,153	Ups	3.61E+09	cytochrome P450%2C family 39%2C subfamily a%2C polypeptide 1 [MGI:1927096]
106	Depp1	-153,476,805,938,576	Down	1.97E+09	DEPP1 autophagy regulator [MGI:1918730]

No	GeneSymbol	log2FoldChange	Regulation	pvalue	Description
107	Dhrs11	-165,301,872,733,256	Down	7.81E+04	dehydrogenase/reductase (SDR family) member 11 [MGI:2652816]
108	Diaph3	-192,093,286,713,031	Down	7.54E+09	diaphanous related formin 3 [MGI:1927222]
109	Dmtn	-105,336,401,684,778	Down	1.88E+07	dematin actin binding protein [MGI:99670]
110	Dnaja4	-187,052,554,979,088	Down	2.27E+09	DnaJ heat shock protein family (Hsp40) member A4 [MGI:1927638]
111	E2f2	-226,320,264,678,766	Down	4.78E+09	E2F transcription factor 2 [MGI:1096341]
112	Ect2	-164,729,789,835,403	Down	9.62E+09	ect2 oncogene [MGI:95281]
113	Esco2	-216,428,235,064,309	Down	9.46E+08	establishment of sister chromatid cohesion N-acetyltransferase 2 [MGI:1919238]
114	Ezh2	-119,707,875,709,064	Down	0.000369242291563766	enhancer of zeste 2 polycomb repressive complex 2 subunit [MGI:107940]
115	F930017D23Rik	-287,637,784,160,574	Down	6.25E+08	RIKEN cDNA F930017D23 gene [MGI:3822538]
116	Fam214b	-101,024,863,230,622	Down	0.00020429704229819	family with sequence similarity 214%2C member B [MGI:2441854]
117	Fcna	-112,222,368,805,879	Down	2.09E+07	ficolin A [MGI:1340905]
118	Fech	-140,101,405,927,984	Down	4.92E+06	ferrochelatase [MGI:95513]
119	Fhdc1	-266,729,930,182,991	Down	3.77E+08	FH2 domain containing 1 [MGI:2684972]
120	Fn3k	-103,660,395,594,078	Down	0.000165946323452658	fructosamine 3 kinase [MGI:1926834]
121	Gch1	-117,227,867,360,931	Down	3.75E+09	GTP cyclohydrolase 1 [MGI:95675]
122	Gm11427	-239,120,976,554,843	Down	3.66E+09	predicted gene 11427 [MGI:3651774]
123	Gm20161	-277,637,692,614,016	Down	8.74E+09	predicted gene%2C 20161 [MGI:5012346]
124	Gm867	-389,758,792,708,744	Down	1.06E+07	predicted gene 867 [MGI:2685713]
125	Gypc	-16,250,739,677,354	Down	0.000339433101808242	glycophorin C [MGI:1098566]
126	H1f3	-290,150,019,841,134	Down	1.38E+08	H1.3 linker histone%2C cluster member [MGI:107502]
127	H1f4	-143,311,521,505,936	Down	2.11E+09	H1.4 linker histone%2C cluster member [MGI:1931527]
128	H2ac8	-234,950,681,647,874	Down	7.57E+09	H2A clustered histone 8 [MGI:2448290]
129	H2bc13	-245,804,616,303,077	Down	0.000243396307915977	H2B clustered histone 13 [MGI:2448403]
130	H4c14	-213,050,386,183,532	Down	3.02E+07	H4 clustered histone 14 [MGI:2140113]
131	H4f16	-209,887,531,675,618	Down	4.06E+06	H4 histone 16 [MGI:2448443]
132	Hba-a1	-267,884,178,180,584	Down	1.31E+09	hemoglobin alpha%2C adult chain 1 [MGI:96015]
133	Hbb-bs	-279,135,020,983,027	Down	1.38E+09	hemoglobin%2C beta adult s chain [MGI:5474852]
134	Hbb-bt	-321,276,121,897,613	Down	8.34E+05	hemoglobin%2C beta adult t chain [MGI:5474850]
135	Hbq1b	-30,574,579,257,002	Down	1.18E+06	hemoglobin%2C theta 1B [MGI:3613460]
136	Hectd4	-112,783,421,896,365	Down	2.22E+09	HECT domain E3 ubiquitin protein ligase 4 [MGI:3647820]
137	Hmbs	-186,315,112,480,729	Down	1.78E+08	hydroxymethylbilane synthase [MGI:96112]
138	Iba57	-107,666,213,410,803	Down	2.56E+09	IBA57 homolog%2C iron-sulfur cluster assembly [MGI:3041174]
139	Josd2	-174,310,313,685,955	Down	1.40E+09	Josephin domain containing 2 [MGI:1913374]
140	Kifc1	-230,598,628,942,981	Down	0.000104248225750566	kinesin family member C1 [MGI:109596]
141	Klhl12	-101,963,022,558,765	Down	2.64E+09	kelch-like 12 [MGI:2385619]
142	Marchf2	-105,857,480,018,057	Down	6.71E+05	membrane associated ring-CH-type finger 2 [MGI:1925915]
143	Mcm10	-15,050,540,986,812	Down	3.41E+09	minichromosome maintenance 10 replication initiation factor [MGI:1917274]
144	Mdk	-114,391,102,477,014	Down	1.67E+09	midkine [MGI:96949]
145	Mkrn1	-134,962,160,758,435	Down	1.57E+06	makorin%2C ring finger protein%2C 1 [MGI:1859353]
146	Mpp2	-15,438,218,291,112	Down	0.000184377176911007	membrane protein%2C palmitoylated 2 (MAGUK p55 subfamily member 2) [MGI:1858257]

No	GeneSymbol	log2FoldChange	Regulation	pvalue	Description
147	Ms4a4c	151,679,701,115,015	Ups	0.000271429537532449	membrane-spanning 4-domains%2C subfamily A%2C member 4C [MGI:1927656]
148	Mup18	-584,767,789,803,362	Down	2.20E+09	major urinary protein 18 [MGI:3705220]
149	Mup7	-415,437,795,458,272	Down	4.88E+09	major urinary protein 7 [MGI:3709615]
150	Nipa1	-184,216,440,132,837	Down	9.92E+09	non imprinted in Prader-Willi/Angelman syndrome 1 homolog (human) [MGI:2442058]
151	Pheta2	-392,182,940,314,767	Down	4.07E+07	PH domain containing endocytic trafficking adaptor 2 [MGI:2443609]
152	Ppp1r15a	-189,474,263,455,583	Down	9.19E+09	protein phosphatase 1%2C regulatory subunit 15A [MGI:1927072]
153	Prdx2	-123,612,529,446,679	Down	1.87E+09	peroxiredoxin 2 [MGI:109486]
154	Ptp4a3	-138,618,346,262,555	Down	3.47E+05	protein tyrosine phosphatase 4a3 [MGI:1277098]
155	Rab3il1	-163,612,577,902,575	Down	2.19E+09	RAB3A interacting protein (rabin3)-like 1 [MGI:1922010]
156	Rad23a	-105,061,434,573,689	Down	0.000353609488111458	RAD23 homolog A%2C nucleotide excision repair protein [MGI:105126]
157	Ranbp10	-134,235,117,272,509	Down	0.000116734686085385	RAN binding protein 10 [MGI:1921584]
158	Rbm38	-206,052,229,077,216	Down	2.84E+09	RNA binding motif protein 38 [MGI:1889294]
159	Samd11	-416,947,987,952,591	Down	2.30E+05	sterile alpha motif domain containing 11 [MGI:2446220]
160	Sh3tc2	-111,777,638,433,933	Down	0.00010175420688751	SH3 domain and tetratricopeptide repeats 2 [MGI:2444417]
161	Slbp	-137,591,157,180,683	Down	0.000155801861354244	stem-loop binding protein [MGI:108402]
162	Slc16a1	-135,759,929,239,972	Down	0.000187808708316891	solute carrier family 16 (monocarboxylic acid transporters)%2C member 1 [MGI:106013]
163	Slc25a37	-263,122,263,355,257	Down	1.79E+09	solute carrier family 25%2C member 37 [MGI:1914962]
164	Slc25a38	-137,107,539,582,326	Down	0.000136581102464859	solute carrier family 25%2C member 38 [MGI:2384782]
165	Slc2a5	183,453,373,166,667	Ups	5.57E+09	solute carrier family 2 (facilitated glucose transporter)%2C member 5 [MGI:1928369]
166	Slc38a5	-235,424,341,955,901	Down	4.77E+09	solute carrier family 38%2C member 5 [MGI:2148066]
167	Slc43a1	-237,794,952,539,596	Down	2.59E+07	solute carrier family 43%2C member 1 [MGI:1931352]
168	Slc4a1	-271,251,801,095,975	Down	0.000327712797160983	solute carrier family 4 (anion exchanger)%2C member 1 [MGI:109393]
169	Snca	-272,506,463,328,835	Down	4.13E+05	synuclein%2C alpha [MGI:1277151]
170	Snx15	-118,199,937,243,131	Down	0.000214603130769184	sorting nexin 15 [MGI:1916274]
171	Sowaha	-268,399,304,079,044	Down	1.85E+07	sosondowah ankyrin repeat domain family member A [MGI:2687280]
172	Specc1	-151,184,828,876,871	Down	1.99E+09	sperm antigen with calponin homology and coiled-coil domains 1 [MGI:2442356]
173	Spib	-104,866,311,790,946	Down	0.000374440892897851	Spi-B transcription factor (Spi-1/PU.1 related) [MGI:892986]
174	Tac2	-603,970,397,604,497	Down	1.83E+09	tachykinin 2 [MGI:98476]
175	Tent5c	-230,846,642,651,683	Down	1.92E+08	terminal nucleotidyltransferase 5C [MGI:1921895]
176	Tfdp2	-105,901,890,592,997	Down	9.99E+08	transcription factor Dp 2 [MGI:107167]
177	Tiaf2	-235,305,234,027,899	Down	0.00164157499277649	TGF-beta1-induced anti-apoptotic factor 2 [MGI:2651383]
178	Tk1	-114,131,526,037,939	Down	3.10E+08	thymidine kinase 1 [MGI:98763]
179	Tmod1	-276,435,507,144,781	Down	5.39E+08	tropomodulin 1 [MGI:98775]
180	Tnip3	157,581,358,248,107	Ups	2.51E+08	TNFAIP3 interacting protein 3 [MGI:3041165]
181	Tspan33	-166,427,700,400,266	Down	3.35E+08	tetraspanin 33 [MGI:1919012]
182	Tyms	-105,617,628,077,643	Down	0.000361468660674234	thymidylate synthase [MGI:98878]
183	Ubac1	-177,579,501,330,778	Down	4.08E+07	ubiquitin associated domain containing 1 [MGI:1920995]
184	Ube2o	-198,262,972,823,706	Down	4.38E+08	ubiquitin-conjugating enzyme E2O [MGI:2444266]
185	Vopp1	-183,845,687,267,396	Down	7.85E+08	vesicular%2C overexpressed in cancer%2C prosurvival protein 1 [MGI:2141658]
186	Xpo7	-143,470,132,975,091	Down	8.67E+06	exportin 7 [MGI:1929705]

Supplementary Table S2C

No	GeneSymbol	log2FoldChange	pvalue	Regulation	Description
1	Spib	-132,748,072,708,051	5.11E+05	Down	Spi-B transcription factor (Spi-1/PU.1 related) [MGI:892986]
2	Dio1	133,710,370,397,247	2.68E+06	Ups	deiodinase%2C iodothyronine%2C type I [MGI:94896]
3	Ighv1-22	-420,957,495,813,907	3.82E+06	Down	immunoglobulin heavy variable 1-22 [MGI:4439784]
4	Mdk	-118,806,261,910,511	5.94E+06	Down	midkine [MGI:96949]
5	Hmgb1-ps7	-412,141,912,021,063	1.91E+07	Down	high-mobility group high mobility group box 1%2C pseudogene 7 [MGI:3648695]
6	Mup20	18,868,604,389,756	6.64E+07	Ups	major urinary protein 20 [MGI:3651981]
7	Fdps	12,372,114,797,374	8.21E+07	Ups	farnesyl diphosphate synthetase [MGI:104888]
8	Npy	602,272,959,705,064	3.49E+08	Ups	neuropeptide Y [MGI:97374]
9	Epx	-45,156,554,518,351	3.66E+08	Down	eosinophil peroxidase [MGI:107569]
10	Pou2af1	-103,540,080,509,055	1.25E+09	Down	POU domain%2C class 2%2C associating factor 1 [MGI:105086]
11	Ubb-ps	133,181,522,544,976	1.95E+09	Ups	ubiquitin B%2C pseudogene [MGI:3037679]
12	Fabp5	178,237,781,158,555	3.42E+09	Ups	fatty acid binding protein 5%2C epidermal [MGI:101790]
13	Kcnt2	-255,960,668,721,962	3.75E+09	Down	potassium channel%2C subfamily T%2C member 2 [MGI:3036273]
14	Ceacam2	132,469,872,046,449	3.99E+09	Ups	carcinoembryonic antigen-related cell adhesion molecule 2 [MGI:1347246]
15	Cd300ld4	-409,992,462,243,739	1.85E+00	Down	CD300 molecule like family member D4 [MGI:3702658]
16	Il21	-278,132,231,991,798	2.10E+09	Down	interleukin 21 [MGI:1890474]
17	Cd209e	-275,156,098,462,265	0.00335308854523479	Down	CD209e antigen [MGI:2157948]
18	Tgif2-ps2	-258,559,071,286,171	0.000300691960238157	Down	TGFB-induced factor homeobox 2%2C pseudogene 2 [MGI:3805950]
19	Il13ra2	-185,023,661,987,733	8.49E-29	Down	interleukin 13 receptor%2C alpha 2 [MGI:1277954]
20	Il4	-176,771,043,178,717	0.00587373036912097	Down	interleukin 4 [MGI:96556]
21	Ccr1l1	-167,556,359,352,467	4.75E+07	Down	chemokine (C-C motif) receptor 1-like 1 [MGI:104617]
22	Ccl22	-152,192,513,662,698	0.000405311671056544	Down	chemokine (C-C motif) ligand 22 [MGI:1306779]
23	Ccr6	-149,131,418,741,072	3.37E+04	Down	chemokine (C-C motif) receptor 6 [MGI:1333797]
24	Tnfrsf8	-143,600,061,613,556	1.01E+09	Down	tumor necrosis factor receptor superfamily%2C member 8 [MGI:99908]
25	Il23r	-137,075,627,434,067	0.00660521731683372	Down	interleukin 23 receptor [MGI:2181693]
26	Tnfrsf9	-129,353,417,056,352	0.0107731970294349	Down	tumor necrosis factor receptor superfamily%2C member 9 [MGI:1101059]
27	Tnfsf8	-109,496,690,026,791	2.56E+09	Down	tumor necrosis factor (ligand) superfamily%2C member 8 [MGI:88328]
28	Tnfrsf19	-102,516,971,073,293	2.37E+07	Down	tumor necrosis factor receptor superfamily%2C member 19 [MGI:1352474]
29	Cd209a	-22,384,928,030,418	2.20E-58	Down	CD209a antigen [MGI:2157942]
30	Ccl24	-14,243,966,528,479	0.000382346343649331	Down	chemokine (C-C motif) ligand 24 [MGI:1928953]
31	Tlr2	101,307,952,521,908	1.86E+07	Ups	toll-like receptor 2 [MGI:1346060]
32	Tnfaip2	102,192,162,448,255	0.0232894217011961	Ups	tumor necrosis factor%2C alpha-induced protein 2 [MGI:104960]
33	Ifi204	104,632,502,092,079	0.0307452258855603	Ups	interferon activated gene 204 [MGI:96429]
34	Irf7	104,744,836,673,836	2.33E+00	Ups	interferon regulatory factor 7 [MGI:1859212]

No	GeneSymbol	log2FoldChange	pvalue	Regulation	Description
35	Ifi205	104,860,323,791,077	0.000175037308649503	Ups	interferon activated gene 205 [MGI:101847]
36	Cd59b	105,788,880,034,178	7.10E+09	Ups	CD59b antigen [MGI:1888996]
37	Tlr8	106,872,911,519,219	7.26E+00	Ups	toll-like receptor 8 [MGI:2176887]
38	Cd209g	107,464,930,628,652	0.0269895576128606	Ups	CD209g antigen [MGI:1917442]
39	Cd300ld5	107,464,930,628,652	5.99E+09	Ups	CD300 molecule like family member D5 [MGI:3702661]
40	Cxcl2	107,464,930,628,652	1.16E-38	Ups	chemokine (C-X-C motif) ligand 2 [MGI:1340094]
41	Ifi44l	107,464,930,628,652	1.63E+02	Ups	interferon-induced protein 44 like [MGI:95975]
42	Il1rapl1	107,464,930,628,652	2.09E+05	Ups	interleukin 1 receptor accessory protein-like 1 [MGI:2687319]
43	Il11ra2	107,464,930,628,652	0.000492740545165748	Ups	interleukin 11 receptor%2C alpha chain 2 [MGI:109123]
44	Tnfsf4	107,464,930,628,652	0.000435017875268627	Ups	tumor necrosis factor (ligand) superfamily%2C member 4 [MGI:104511]
45	Tnfaip6	107,464,930,628,652	0.000928373184333609	Ups	tumor necrosis factor alpha induced protein 6 [MGI:1195266]
46	Il12b	107,665,581,283,575	0.00874689609902386	Ups	interleukin 12b [MGI:96540]
47	Ccl8	108,005,364,655,727	2.58E-03	Ups	chemokine (C-C motif) ligand 8 [MGI:101878]
48	Tlr6	110,473,157,865,015	9.46E+09	Ups	toll-like receptor 6 [MGI:1341296]
49	Tnfrsf23	111,507,117,297,136	1.08E+00	Ups	tumor necrosis factor receptor superfamily%2C member 23 [MGI:1930269]
50	Tnfsf13os	113,219,294,110,661	0.0156911201266431	Ups	tumor necrosis factor (ligand) superfamily%2C member 13%2C opposite strand [MGI:1919587]
51	Tlr13	115,547,341,421,694	1.05E+08	Ups	toll-like receptor 13 [MGI:3045213]
52	Cd300e	116,142,686,461,352	0.00272713485805022	Ups	CD300E molecule [MGI:2387602]
53	Ifi44	116,621,788,027,778	3.52E+04	Ups	interferon-induced protein 44 [MGI:2443016]
54	Ifit1bl1	118,674,903,809,899	2.95E+08	Ups	interferon induced protein with tetratricpeptide repeats 1B like 1 [MGI:3650685]
55	Ifit2	118,785,956,463,145	8.70E+05	Ups	interferon-induced protein with tetratricopeptide repeats 2 [MGI:99449]
56	Il9r	119,277,456,645,938	3.43E+09	Ups	interleukin 9 receptor [MGI:96564]
57	Ifit1	120,926,818,238,388	4.45E+00	Ups	interferon-induced protein with tetratricopeptide repeats 1 [MGI:99450]
58	Il18bp	121,778,142,923,443	0.0321899239374584	Ups	interleukin 18 binding protein [MGI:1333800]
59	Ifi211	122,826,655,903,682	7.81E+07	Ups	interferon activated gene 211 [MGI:3041120]
60	Ifi202b	124,045,377,940,071	2.90E+04	Ups	interferon activated gene 202B [MGI:1347083]
61	Ifi27l2b	125,657,187,311,355	0.000103171301877075	Ups	interferon%2C alpha-inducible protein 27 like 2B [MGI:1916390]
62	Ifit3	130,305,503,452,759	0.0025536018126923	Ups	interferon-induced protein with tetratricopeptide repeats 3 [MGI:1101055]
63	Cxcl9	131,528,690,326,603	2.36E+08	Ups	chemokine (C-X-C motif) ligand 9 [MGI:1352449]
64	Il22ra2	154,359,794,387,995	0.0162435650732066	Ups	interleukin 22 receptor%2C alpha 2 [MGI:2665114]
65	Il12rb2	175,400,760,761,824	1.04E-06	Ups	interleukin 12 receptor%2C beta 2 [MGI:1270861]
66	Cd207	178,061,945,229,975	5.13E+04	Ups	CD207 antigen [MGI:2180021]
67	Il10	183,995,911,848,254	1.15E+03	Ups	interleukin 10 [MGI:96537]
68	Cxcl3	187,324,215,869,305	7.61E+01	Ups	chemokine (C-X-C motif) ligand 3 [MGI:3037818]
69	Tnip3	187,434,628,829,558	1.65E-08	Ups	TNFAIP3 interacting protein 3 [MGI:3041165]
70	Cd200r2	191,817,943,613,238	2.84E+06	Ups	Cd200 receptor 2 [MGI:3042847]
