# Supplementary material for: Identification of immune-associated genes with altered expression in the spleen of mice enriched with probiotic Lactobacillus species using RNA-seq profiling
Source: Anim Biosci. 2024 Aug 26;38(2):336–49. doi: 10.5713/ab.24.0280 (PMC11725755; doi:10.5713/ab.24.0280)
Supplement: Supplementary file 4 [file ab-24-0280-Supplementary-Table-3.pdf]

No	GeneSymbol	log2FoldChange	pvalue	Regulation	Description
71	Il4i1	191,817,943,613,238	0.000237475369019883	Ups	interleukin 4 induced 1 [MGI:109552]
72	Cd14	202,641,041,714,651	9.37E+02	Ups	CD14 antigen [MGI:88318]
73	Cd1d2	203,405,396,653,538	0.00832009748099911	Ups	CD1d2 antigen [MGI:107675]
74	Il1rn	209,036,679,328,658	5.41E+08	Ups	interleukin 1 receptor antagonist [MGI:96547]
75	Il12rb1	214,328,629,045,949	1.67E+08	Ups	interleukin 12 receptor%2C beta 1 [MGI:104579]
76	Cxcl17	230,065,376,966,381	1.37E+09	Ups	chemokine (C-X-C motif) ligand 17 [MGI:2387642]
77	Ifnk	234,286,306,906,596	0.142881181304276	Ups	interferon kappa [MGI:2683287]
78	Ifitm7	240,523,715,532,405	0.548094718159512	Ups	interferon induced transmembrane protein 7 [MGI:1921732]
79	Il11	263,539,363,972,229	0.174428187317255	Ups	interleukin 11 [MGI:107613]
80	Cd163	307,964,851,730,233	0.00964409848365247	Ups	CD163 antigen [MGI:2135946]
81	Ccl28	326,910,508,634,092	0.0496813409682616	Ups	chemokine (C-C motif) ligand 28 [MGI:1861731]
82	Cd200r4	461,113,034,685,918	0.240453497829044	Ups	CD200 receptor 4 [MGI:3036289]

Supplementary Table S3A

No	GeneSymbol	log2FoldChange	pvalue	Description
1	Ifi44l	-467,208,678,222,942	0.0250325998880863	interferon-induced protein 44 like [MGI:95975]
2	Ifitm7	-352,441,339,801,377	0.038499424806822	interferon induced transmembrane protein 7 [MGI:1921732]
3	Isg20	-299,399,426,802,496	1.13E+06	interferon-stimulated protein [MGI:1928895]
4	Cd24a	-287,549,502,780,372	1.85E+00	CD24a antigen [MGI:88323]
5	Cd163	-263,796,676,794,267	2.10E+09	CD163 antigen [MGI:2135946]
6	Ccl28	-241,922,854,405,425	0.00335308854523479	chemokine (C-C motif) ligand 28 [MGI:1861731]
7	Tiaf2	-230,606,655,703,201	0.000300691960238157	TGF-beta1-induced anti-apoptotic factor 2 [MGI:2651383]
8	Ccl1	-214,632,537,480,107	8.49E-29	chemokine (C-C motif) ligand 1 [MGI:98258]
9	Il1rl1	-188,637,578,938,601	0.00587373036912097	interleukin 1 receptor-like 1 [MGI:98427]
10	Cd209f	-187,465,718,291,774	4.75E+07	CD209f antigen [MGI:1916392]
11	Cd59b	-177,786,408,180,693	0.000405311671056544	CD59b antigen [MGI:1888996]
12	Tnfsf11	-124,549,286,001,411	3.37E+04	tumor necrosis factor (ligand) superfamily%2C member 11 [MGI:1100089]
13	Cd1d2	-116,963,989,627,878	1.01E+09	CD1d2 antigen [MGI:107675]
14	Cd200r4	-112,781,139,803,128	0.00660521731683372	CD200 receptor 4 [MGI:3036289]
15	Cd209g	-112,781,139,803,128	0.0107731970294349	CD209g antigen [MGI:1917442]
16	Cd300ld5	-112,781,139,803,128	2.56E+09	CD300 molecule like family member D5 [MGI:3702661]
17	Cxcl2	-112,781,139,803,128	2.37E+07	chemokine (C-X-C motif) ligand 2 [MGI:1340094]
18	Il11ra2	-112,781,139,803,128	2.20E-58	interleukin 11 receptor%2C alpha chain 2 [MGI:109123]
19	Tnfsf4	-112,781,139,803,128	0.000382346343649331	tumor necrosis factor (ligand) superfamily%2C member 4 [MGI:104511]
20	Il9r	-111,699,687,487,171	1.86E+07	interleukin 9 receptor [MGI:96564]
21	Tlr5	-110,926,096,539,279	0.0232894217011961	toll-like receptor 5 [MGI:1858171]

No	GeneSymbol	log2FoldChange	pvalue	Description
22	Il17d	-107,338,639,321,485	0.0307452258855603	interleukin 17D [MGI:2446510]
23	Ifrd2	-105,344,449,381,285	2.33E+00	interferon-related developmental regulator 2 [MGI:1316708]
24	Il36rn	-20,476,862,561,024	0.000175037308649503	interleukin 36 receptor antagonist [MGI:1859325]
25	Tnfrsf14	-12,835,308,350,574	7.10E+09	tumor necrosis factor receptor superfamily%2C member 14 (herpesvirus entry mediator) [MGI:2675303]
26	Iigp1	11,642,690,128,155	7.26E+00	interferon inducible GTPase 1 [MGI:1926259]
27	Cxcl5	13,450,487,601,445	0.0269895576128606	chemokine (C-X-C motif) ligand 5 [MGI:1096868]
28	Ccl24	20,382,068,070,276	5.99E+09	chemokine (C-C motif) ligand 24 [MGI:1928953]
29	Ifi204	106,550,898,066,082	1.16E-38	interferon activated gene 204 [MGI:96429]
30	Cd8a	111,441,055,724,983	1.63E+02	CD8 antigen%2C alpha chain [MGI:88346]
31	Tnfrsf19	111,907,388,148,338	2.09E+05	tumor necrosis factor receptor superfamily%2C member 19 [MGI:1352474]
32	Cd3e	112,232,171,827,061	0.000492740545165748	CD3 antigen%2C epsilon polypeptide [MGI:88332]
33	Ccl19	113,220,977,396,178	0.000435017875268627	chemokine (C-C motif) ligand 19 [MGI:1346316]
34	Ifi211	113,686,099,434,364	0.000928373184333609	interferon activated gene 211 [MGI:3041120]
35	Cd177	113,999,749,539,974	0.00874689609902386	CD177 antigen [MGI:1916141]
36	Cxcr6	115,043,957,321,004	2.58E-03	chemokine (C-X-C motif) receptor 6 [MGI:1934582]
37	Ifi208	116,233,763,451,878	9.46E+09	interferon activated gene 208 [MGI:2442822]
38	Xcl1	116,400,312,581,627	1.08E+00	chemokine (C motif) ligand 1 [MGI:104593]
39	Ccl17	116,847,167,025,075	0.0156911201266431	chemokine (C-C motif) ligand 17 [MGI:1329039]
40	Ccl5	117,613,726,547,613	1.05E+08	chemokine (C-C motif) ligand 5 [MGI:98262]
41	Cd22	119,758,325,880,075	0.00272713485805022	CD22 antigen [MGI:88322]
42	Il18rap	120,258,689,091,241	3.52E+04	interleukin 18 receptor accessory protein [MGI:1338888]
43	Cd69	121,317,090,676,902	2.95E+08	CD69 antigen [MGI:88343]
44	Ticam2	121,678,929,611,201	8.70E+05	toll-like receptor adaptor molecule 2 [MGI:3040056]
45	Cxcl11	122,876,839,245,862	3.43E+09	chemokine (C-X-C motif) ligand 11 [MGI:1860203]
46	Il1a	122,899,081,969,256	4.45E+00	interleukin 1 alpha [MGI:96542]
47	Ifi205	122,982,285,788,749	0.0321899239374584	interferon activated gene 205 [MGI:101847]
48	Cxcl10	124,406,146,179,967	7.81E+07	chemokine (C-X-C motif) ligand 10 [MGI:1352450]
49	Ccl7	125,177,214,306,982	2.90E+04	chemokine (C-C motif) ligand 7 [MGI:99512]
50	Cd8b1	125,198,326,895,244	0.000103171301877075	CD8 antigen%2C beta chain 1 [MGI:88347]
51	Tnfrsf4	126,360,264,547,992	0.0025536018126923	tumor necrosis factor receptor superfamily%2C member 4 [MGI:104512]
52	Il18r1	126,642,230,201,673	2.36E+08	interleukin 18 receptor 1 [MGI:105383]
53	Tgif2-ps2	127,345,586,880,791	0.0162435650732066	TGFB-induced factor homeobox 2%2C pseudogene 2 [MGI:3805950]
54	Cd300lf	128,952,839,438,254	1.04E-06	CD300 molecule like family member F [MGI:2442359]
55	Il7	130,036,094,444,114	5.13E+04	interleukin 7 [MGI:96561]
56	Il36g	130,453,096,106,341	1.15E+03	interleukin 36G [MGI:2449929]
57	Ifi213	131,499,813,677,011	7.61E+01	interferon activated gene 213 [MGI:3695276]
58	Ifi206	133,873,252,853,677	1.65E-08	interferon activated gene 206 [MGI:3646410]
59	Il10	134,251,419,581,982	2.84E+06	interleukin 10 [MGI:96537]
60	Cd200r3	134,757,345,757,161	0.000237475369019883	CD200 receptor 3 [MGI:1921853]
61	Tnfrsf26	138,654,913,882,774	9.37E+02	tumor necrosis factor receptor superfamily%2C member 26 [MGI:2651928]

No	GeneSymbol	log2FoldChange	pvalue	Description
62	Tnfrsf8	140,756,226,052,026	0.00832009748099911	tumor necrosis factor receptor superfamily%2C member 8 [MGI:99908]
63	Cd274	143,210,544,215,653	5.41E+08	CD274 antigen [MGI:1926446]
64	Tnfaip8l3	148,687,301,380,935	1.67E+08	tumor necrosis factor%2C alpha-induced protein 8-like 3 [MGI:2685363]
65	Ccl4	148,822,594,240,585	1.37E+09	chemokine (C-C motif) ligand 4 [MGI:98261]
66	Ccr5	150,841,866,476,315	0.0189967506500919	chemokine (C-C motif) receptor 5 [MGI:107182]
67	Cxcl9	156,939,248,347,876	0.00324762495805923	chemokine (C-X-C motif) ligand 9 [MGI:1352449]
68	Ccr1l1	162,786,819,626,243	0.0020351074049379	chemokine (C-C motif) receptor 1-like 1 [MGI:104617]
69	Cxcr1	162,786,819,626,243	7.73E-16	chemokine (C-X-C motif) receptor 1 [MGI:2448715]
70	Tnfsf9	167,968,548,512,929	1.48E+02	tumor necrosis factor (ligand) superfamily%2C member 9 [MGI:1101058]
71	Ifnk	168,109,598,074,735	0.00391146708484	interferon kappa [MGI:2683287]
72	Tnip3	169,436,520,457,026	0.016550983947835	TNFAIP3 interacting protein 3 [MGI:3041165]
73	Ccr4	170,789,845,406,505	2.89E+07	chemokine (C-C motif) receptor 4 [MGI:107824]
74	Il21	176,485,807,999,696	0.00301030709618037	interleukin 21 [MGI:1890474]
75	Tnf	177,345,566,054,297	1.32E-06	tumor necrosis factor [MGI:104798]
76	Cd207	188,047,703,676,007	8.08E+05	CD207 antigen [MGI:2180021]
77	Tnfrsf9	201,992,426,611,829	1.83E-01	tumor necrosis factor receptor superfamily%2C member 9 [MGI:1101059]
78	Cxcl1	208,591,742,387,313	6.77E+09	chemokine (C-X-C motif) ligand 1 [MGI:108068]
79	Il31ra	230,066,482,602,652	7.27E+02	interleukin 31 receptor A [MGI:2180511]
80	Cd209e	269,341,761,322,336	5.60E-03	CD209e antigen [MGI:2157948]
81	Ccr10	273,564,247,573,611	0.00579863342357507	chemokine (C-C motif) receptor 10 [MGI:1096320]
82	Il27	290,226,373,798,985	3.27E+08	interleukin 27 [MGI:2384409]
83	Cd300c	303,442,553,022,332	0.000193374985166703	CD300C molecule [MGI:3032626]
84	Cd3eap	-0.00262089806457713	0.991332037454473	CD3E antigen%2C epsilon polypeptide associated protein [MGI:1917583]
85	Cd1d1	-0.00619633600936429	0.976614420461485	CD1d1 antigen [MGI:107674]
86	Trap1	-0.00846184716646613	0.953671786618487	TNF receptor-associated protein 1 [MGI:1915265]
87	Tnfrsf12a	-0.00925238412591791	0.977018660168316	tumor necrosis factor receptor superfamily%2C member 12a [MGI:1351484]
88	Traf5	-0.0126519064498539	0.965838205200276	TNF receptor-associated factor 5 [MGI:107548]
89	Cxcl13	-0.0140123739583393	0.949941871527495	chemokine (C-X-C motif) ligand 13 [MGI:1888499]
90	Cklf	-0.0185708272101903	0.971623117484487	chemokine-like factor [MGI:1922708]
91	Tab2	-0.0253328414724077	0.86152115551637	TGF-beta activated kinase 1/MAP3K7 binding protein 2 [MGI:1915902]
92	Tlr2	-0.0302363669344413	0.932851690666907	toll-like receptor 2 [MGI:1346060]
93	Il1rap	-0.0361556684447257	0.831354566766087	interleukin 1 receptor accessory protein [MGI:104975]
94	Cd46	-0.0384902425299949	0.957985648443707	CD46 antigen%2C complement regulatory protein [MGI:1203290]
95	Cxcr4	-0.0396150663657473	0.84656279928723	chemokine (C-X-C motif) receptor 4 [MGI:109563]
96	Ifi27l2a	-0.046149681041222	0.831374233118246	interferon%2C alpha-inducible protein 27 like 2A [MGI:1924183]
97	Cd151	-0.0578317523866776	0.729767623705224	CD151 antigen [MGI:1096360]
98	Cd93	-0.0582431492956896	0.782845333661468	CD93 antigen [MGI:106664]
99	Il11ra1	-0.0608153891863879	0.772410743680677	interleukin 11 receptor%2C alpha chain 1 [MGI:107426]
100	Ifnar1	-0.0613802660674209	0.759489773427156	interferon (alpha and beta) receptor 1 [MGI:107658]
101	Ccl11	-0.0621951984525066	0.960515525944714	chemokine (C-C motif) ligand 11 [MGI:103576]

No	GeneSymbol	log2FoldChange	pvalue	Description
102	Traf7	-0.0762018539447005	0.697470525564974	TNF receptor-associated factor 7 [MGI:3042141]
103	Cd300lg	-0.0801275154884809	0.757465061066638	CD300 molecule like family member G [MGI:1289168]
104	Ccl3	-0.0941071943622083	0.948508143810055	chemokine (C-C motif) ligand 3 [MGI:98260]
105	Cd300c2	-0.101059902577016	0.74293270157257	CD300C molecule 2 [MGI:2153249]
106	Ifnar2	-0.102920997919415	0.566208679723252	interferon (alpha and beta) receptor 2 [MGI:1098243]
107	Tnfsf14	-0.105258351757071	0.843708968230058	tumor necrosis factor (ligand) superfamily%2C member 14 [MGI:1355317]
108	Irf7	-0.109260756784701	0.778640020658634	interferon regulatory factor 7 [MGI:1859212]
109	Irf3	-0.11073813354296	0.546016345404074	interferon regulatory factor 3 [MGI:1859179]
110	Ifnlr1	-0.118381414245394	0.870408475771248	interferon lambda receptor 1 [MGI:2429859]
111	Traf2	-0.133627553302836	0.583244219505314	TNF receptor-associated factor 2 [MGI:101835]
112	Cd63	-0.142505918491446	0.475047267908515	CD63 antigen [MGI:99529]
113	Cd2	-0.144492400320381	0.692192432964136	CD2 antigen [MGI:88320]
114	Cd99l2	-0.15201955393517	0.409865738189925	CD99 antigen-like 2 [MGI:2177151]
115	Irak4	-0.156865865556092	0.600204967388373	interleukin-1 receptor-associated kinase 4 [MGI:2182474]
116	Ifitm5	-0.166030718602419	0.967543761014425	interferon induced transmembrane protein 5 [MGI:1934923]
117	Il17b	-0.166030718602419	0.967543761014425	interleukin 17B [MGI:1928397]
118	Il2	-0.166030718602419	0.967543761014425	interleukin 2 [MGI:96548]
119	Il22ra2	-0.166030718602419	0.967543761014425	interleukin 22 receptor%2C alpha 2 [MGI:2665114]
120	Ifitm10	-0.171348496814841	0.798364362740487	interferon induced transmembrane protein 10 [MGI:2444776]
121	Cd59a	-0.17498586057669	0.424239720311483	CD59a antigen [MGI:109177]
122	Cd63-ps	-0.179247864588779	0.376320403031747	CD63 antigen%2C pseudogene [MGI:105972]
123	Ccl12	-0.18993698658792	0.920563026382899	chemokine (C-C motif) ligand 12 [MGI:108224]
124	Cd34	-0.190627831923156	0.479093502184157	CD34 antigen [MGI:88329]
125	Cd7	-0.1921551502642	0.642516172302029	CD7 antigen [MGI:88344]
126	Cd5l	-0.196401576458325	0.380876783756348	CD5 antigen-like [MGI:1334419]
127	Cd81	-0.19812577997359	0.249178183376981	CD81 antigen [MGI:1096398]
128	Tnfrsf11a	-0.217550868134337	0.59945360055784	tumor necrosis factor receptor superfamily%2C member 11a%2C NFKB activator [MGI:1314891]
129	Tnfrsf23	-0.236071494728239	0.760046811426956	tumor necrosis factor receptor superfamily%2C member 23 [MGI:1930269]
130	Traf4	-0.27905371563932	0.14319395567051	TNF receptor associated factor 4 [MGI:1202880]
131	Ccr3	-0.281656739915279	0.347426081474393	chemokine (C-C motif) receptor 3 [MGI:104616]
132	Cd82	-0.288872163234644	0.0439961508075757	CD82 antigen [MGI:104651]
133	Irak1	-0.305869436114942	0.0363014153504541	interleukin-1 receptor-associated kinase 1 [MGI:107420]
134	Ifit1	-0.312286661382303	0.425747770855162	interferon-induced protein with tetratricopeptide repeats 1 [MGI:99450]
135	Tradd	-0.32084418319158	0.278902182416941	TNFRSF1A-associated via death domain [MGI:109200]
136	Cd109	-0.358104629938749	0.745508358824141	CD109 antigen [MGI:2445221]
137	Il12rb2	-0.383878764950175	0.746929799831355	interleukin 12 receptor%2C beta 2 [MGI:1270861]
138	Irf6	-0.428169906557162	0.108744610527716	interferon regulatory factor 6 [MGI:1859211]
139	Il23r	-0.440425466724552	0.831086014133864	interleukin 23 receptor [MGI:2181693]
140	Ifi27	-0.447231251319332	0.041460446116183	interferon%2C alpha-inducible protein 27 [MGI:1277180]
141	Cd320	-0.459428918086463	0.0636900255722184	CD320 antigen [MGI:1860083]

No	GeneSymbol	log2FoldChange	pvalue	Description
142	Cd47	-0.491028547007987	0.00531923417079533	CD47 antigen (Rh-related antigen%2C integrin-associated signal transducer) [MGI:96617]
143	Il12b	-0.492416373035835	0.824904907360906	interleukin 12b [MGI:96540]
144	Tnfrsf22	-0.495120144135035	0.523536279657579	tumor necrosis factor receptor superfamily%2C member 22 [MGI:1930270]
145	Tab1	-0.521103362069085	0.077328950388393	TGF-beta activated kinase 1/MAP3K7 binding protein 1 [MGI:1913763]
146	Tnfsf15	-0.521400568599564	0.339253828524011	tumor necrosis factor (ligand) superfamily%2C member 15 [MGI:2180140]
147	Il5ra	-0.551261011170715	0.393641958983789	interleukin 5 receptor%2C alpha [MGI:96558]
148	Ccr9	-0.567195082782	0.419583623003574	chemokine (C-C motif) receptor 9 [MGI:1341902]
149	Tnfrsf17	-0.619169651463248	0.727280101298829	tumor necrosis factor receptor superfamily%2C member 17 [MGI:1343050]
150	Cd36	-0.633591974469062	0.00491284423549008	CD36 molecule [MGI:107899]
151	Ccrl2	-0.682958015456919	0.0723671883780414	chemokine (C-C motif) receptor-like 2 [MGI:1920904]
152	Cd276	-0.69303052016331	0.164651744179628	CD276 antigen [MGI:2183926]
153	Il34	-0.729289410143117	0.0772264665344376	interleukin 34 [MGI:1923777]
154	Cxcl14	-0.731018774539997	0.0367566196281666	chemokine (C-X-C motif) ligand 14 [MGI:1888514]
155	Cd101	-0.751461888912382	0.580966647610969	CD101 antigen [MGI:2685862]
156	Tab3	-0.751892440678	0.000658642078124466	TGF-beta activated kinase 1/MAP3K7 binding protein 3 [MGI:1913974]
157	Cd164l2	-0.789286342584549	0.290331722899568	CD164 sialomucin-like 2 [MGI:1916905]
158	Tnfaip2	-0.813354828952169	2.56E+09	tumor necrosis factor%2C alpha-induced protein 2 [MGI:104960]
159	Ccr8	-0.836351016506273	0.827928642888937	chemokine (C-C motif) receptor 8 [MGI:1201402]
160	Tnfaip8l1	-0.851482091279983	0.024296157655913	tumor necrosis factor%2C alpha-induced protein 8-like 1 [MGI:1913693]
161	Ccl27a	-0.899256964009961	0.179736175590395	chemokine (C-C motif) ligand 27A [MGI:1343459]
162	Irf9	0.00578129580675873	0.971158860904259	interferon regulatory factor 9 [MGI:107587]
163	Tgif1	0.0078571917397229	0.976290648676561	TGFB-induced factor homeobox 1 [MGI:1194497]
164	Il12a	0.00800953944136572	0.993310395019588	interleukin 12a [MGI:96539]
165	Ifrd1	0.0100593475575256	0.97283682725776	interferon-related developmental regulator 1 [MGI:1316717]
166	Tlr12	0.0199029546257431	0.968797101890282	toll-like receptor 12 [MGI:3045221]
167	Il17ra	0.0217756898963605	0.913141631770644	interleukin 17 receptor A [MGI:107399]
168	Tlr6	0.0328116779623929	0.973225885879667	toll-like receptor 6 [MGI:1341296]
169	Il18	0.0357724627932457	0.897020380319818	interleukin 18 [MGI:107936]
170	Cd2bp2	0.0458398108531906	0.819039788597307	CD2 cytoplasmic tail binding protein 2 [MGI:1917483]
171	Tnfaip8	0.0483327950074373	0.774699246540361	tumor necrosis factor%2C alpha-induced protein 8 [MGI:2147191]
172	Tnfsf13os	0.0516247136939314	0.980314827981839	tumor necrosis factor (ligand) superfamily%2C member 13%2C opposite strand [MGI:1919587]
173	Cd68	0.0539403258353834	0.798912083013518	CD68 antigen [MGI:88342]
174	Tlr8	0.0541136621182674	0.926818415965238	toll-like receptor 8 [MGI:2176887]
175	Irak2	0.0566807490615935	0.790486764154397	interleukin-1 receptor-associated kinase 2 [MGI:2429603]
176	Tnfrsf25	0.0581771357945654	0.948853502808462	tumor necrosis factor receptor superfamily%2C member 25 [MGI:1934667]
177	Irak1bp1	0.0632242855281988	0.886066641861696	interleukin-1 receptor-associated kinase 1 binding protein 1 [MGI:1929475]
178	Il17rc	0.0713916328161358	0.826940559242956	interleukin 17 receptor C [MGI:2159336]
179	Cd55	0.0973200597254049	0.741833131168513	CD55 molecule%2C decay accelerating factor for complement [MGI:104850]
180	Cd300ld4	0.102312397026384	0.939944294541932	CD300 molecule like family member D4 [MGI:3702658]
181	Il1b	0.11666501999121	0.849589182486489	interleukin 1 beta [MGI:96543]

No	GeneSymbol	log2FoldChange	pvalue	Description
182	Tnfaip1	0.120519793083749	0.484878709400778	tumor necrosis factor%2C alpha-induced protein 1 (endothelial) [MGI:104961]
183	Cd55os	0.122420591753774	0.913292075452116	CD55 molecule%2C opposite strand sequence [MGI:3783116]
184	Ilf2	0.131721339231805	0.532707105175246	interleukin enhancer binding factor 2 [MGI:1915031]
185	Il3ra	0.135094984150729	0.802368952392734	interleukin 3 receptor%2C alpha chain [MGI:96553]
186	Cd164	0.135274463324251	0.374850489058394	CD164 antigen [MGI:1859568]
187	Tnfsfm13	0.1362115869742	0.810843380875184	tumor necrosis factor (ligand) superfamily%2C membrane-bound member 13 [MGI:3845075]
188	Il20rb	0.139736995709714	0.952099196144495	interleukin 20 receptor beta [MGI:2143266]
189	Ifngr2	0.140328400504836	0.490671531071914	interferon gamma receptor 2 [MGI:107654]
190	Il15	0.147052804314778	0.707063790410625	interleukin 15 [MGI:103014]
191	Tnfrsf10b	0.148759127381646	0.837409721499781	tumor necrosis factor receptor superfamily%2C member 10b [MGI:1341090]
192	Ticam1	0.149015628252634	0.541499683238755	toll-like receptor adaptor molecule 1 [MGI:2147032]
193	Il33	0.155686805402557	0.656889333014612	interleukin 33 [MGI:1924375]
194	Ifit1bl1	0.157399841767243	0.70495420187187	interferon induced protein with tetratricpeptide repeats 1B like 1 [MGI:3650685]
195	Irf2bp2	0.163207052582951	0.255816463023966	interferon regulatory factor 2 binding protein 2 [MGI:2443921]
196	Ccl9	0.169833557908595	0.418930776655877	chemokine (C-C motif) ligand 9 [MGI:104533]
197	Ifi30	0.171346654587207	0.253951212296436	interferon gamma inducible protein 30 [MGI:2137648]
198	Irf2bp1	0.171969081381717	0.417878973681996	interferon regulatory factor 2 binding protein 1 [MGI:2442159]
199	Ccl25	0.173940025582159	0.704575226931074	chemokine (C-C motif) ligand 25 [MGI:1099448]
200	Il15ra	0.185353859360625	0.504805871708158	interleukin 15 receptor%2C alpha chain [MGI:104644]
201	Ifih1	0.198847034938949	0.325883747171355	interferon induced with helicase C domain 1 [MGI:1918836]
202	Tnfrsf11b	0.204560390222012	0.715772080153654	tumor necrosis factor receptor superfamily%2C member 11b (osteoprotegerin) [MGI:109587]
203	Cxcl16	0.211120547627169	0.322489962102074	chemokine (C-X-C motif) ligand 16 [MGI:1932682]
204	Ccl22	0.219307400097519	0.752750090130745	chemokine (C-C motif) ligand 22 [MGI:1306779]
205	Il17f	0.222187994632948	0.906629352628846	interleukin 17F [MGI:2676631]
206	Il17re	0.225903465632429	0.669837852411572	interleukin 17 receptor E [MGI:1889371]
207	Cx3cr1	0.234927905142528	0.583789260154121	chemokine (C-X3-C motif) receptor 1 [MGI:1333815]
208	Traf3	0.237195416990574	0.407806211016947	TNF receptor-associated factor 3 [MGI:108041]
209	Il17rd	0.240605717898909	0.584651823494223	interleukin 17 receptor D [MGI:2159727]
210	Cx3cl1	0.241846044798438	0.320439256069755	chemokine (C-X3-C motif) ligand 1 [MGI:1097153]
211	Ccl19-ps3	0.246422764009725	0.92129214115864	chemokine (C-C motif) ligand 19%2C pseudogene 3 [MGI:1891391]
212	Ifit1bl2	0.258141488667347	0.679195045825905	interferon induced protein with tetratricopeptide repeats 1B like 2 [MGI:2148249]
213	Tnfrsf21	0.269802829311968	0.188868662745486	tumor necrosis factor receptor superfamily%2C member 21 [MGI:2151075]
214	Cd248	0.276255287972712	0.46788532869847	CD248 antigen%2C endosialin [MGI:1917695]
215	Il10rb	0.277632200316675	0.185765188633765	interleukin 10 receptor%2C beta [MGI:109380]
216	Cd44	0.278436637529509	0.0816991261541565	CD44 antigen [MGI:88338]
217	Il4ra	0.278673910210636	0.226695040687524	interleukin 4 receptor%2C alpha [MGI:105367]
218	Ifitm2	0.28155299351991	0.0518562182742356	interferon induced transmembrane protein 2 [MGI:1933382]
219	Ifitm3	0.284052306820555	0.151495144257277	interferon induced transmembrane protein 3 [MGI:1913391]
220	Ifitm1	0.285869071018815	0.183058310401998	interferon induced transmembrane protein 1 [MGI:1915963]
221	Cd52	0.286126719788438	0.123721781445766	CD52 antigen [MGI:1346088]

No	GeneSymbol	log2FoldChange	pvalue	Description
222	Il13ra1	0.287625727742269	0.113086776264654	interleukin 13 receptor%2C alpha 1 [MGI:105052]
223	Ilf3	0.289842650277837	0.137526102020061	interleukin enhancer binding factor 3 [MGI:1339973]
224	Il7r	0.295885538234105	0.443324935078424	interleukin 7 receptor [MGI:96562]
225	Ccr7	0.298539109973693	0.427001910970691	chemokine (C-C motif) receptor 7 [MGI:103011]
226	Cd200	0.300050777218861	0.183788615413839	CD200 antigen [MGI:1196990]
227	Cd2ap	0.302564648150759	0.0629944511950471	CD2-associated protein [MGI:1330281]
228	Cd84	0.30261684604872	0.235854335728596	CD84 antigen [MGI:1336885]
229	Tnip1	0.303100464479111	0.100260577411036	TNFAIP3 interacting protein 1 [MGI:1926194]
230	Il27ra	0.303254191499219	0.41640259561368	interleukin 27 receptor%2C alpha [MGI:1355318]
231	Il10ra	0.306110691680129	0.262934810380336	interleukin 10 receptor%2C alpha [MGI:96538]
232	Ccl8	0.308417593486029	0.832893591234898	chemokine (C-C motif) ligand 8 [MGI:101878]
233	Cd9	0.30956038962614	0.0732039987392036	CD9 antigen [MGI:88348]
234	Ifit2	0.312363410961508	0.581592890060422	interferon-induced protein with tetratricopeptide repeats 2 [MGI:99449]
235	Tlr11	0.318154398174146	0.598777278201427	toll-like receptor 11 [MGI:3045226]
236	Ifngr1	0.325760726511796	0.0954568966714441	interferon gamma receptor 1 [MGI:107655]
237	Irf2bpl	0.329905132240616	0.0600060633626642	interferon regulatory factor 2 binding protein-like [MGI:2442463]
238	Cd96	0.335206298844372	0.524470126909059	CD96 antigen [MGI:1934368]
239	Cd40lg	0.338460331425115	0.686015409202497	CD40 ligand [MGI:88337]
240	Il6st	0.350926435789585	0.0123179059689625	interleukin 6 signal transducer [MGI:96560]
241	Irf2	0.353297853088101	0.0475117080556533	interferon regulatory factor 2 [MGI:96591]
242	Cd200r1	0.360866267081491	0.604257244948017	CD200 receptor 1 [MGI:1889024]
243	Il4	0.361012280532992	0.707998880866982	interleukin 4 [MGI:96556]
244	Tnip2	0.361786268541343	0.374758596010122	TNFAIP3 interacting protein 2 [MGI:2386643]
245	Isg20l2	0.362339612067662	0.135197469947919	interferon stimulated exonuclease gene 20-like 2 [MGI:2140076]
246	Il1rn	0.362816603090356	0.447144462670158	interleukin 1 receptor antagonist [MGI:96547]
247	Cd300lb	0.370484261708807	0.545677463736596	CD300 molecule like family member B [MGI:2685099]
248	Ifi35	0.382212563689015	0.125112041034184	interferon-induced protein 35 [MGI:1917360]
249	Cd37	0.390350955481076	0.0735484983212959	CD37 antigen [MGI:88330]
250	Il12rb1	0.391975061330469	0.673185903519082	interleukin 12 receptor%2C beta 1 [MGI:104579]
251	Cd302	0.399478961570796	0.00525976115575836	CD302 antigen [MGI:1913455]
252	Traf6	0.400177540021195	0.0465748235446407	TNF receptor-associated factor 6 [MGI:108072]
253	Ifit3	0.400498849422531	0.579809997848773	interferon-induced protein with tetratricopeptide repeats 3 [MGI:1101055]
254	Ifi214	0.404757580933885	0.388448663685583	interferon activated gene 214 [MGI:3584522]
255	Cd79b	0.406345554211332	0.0526700847937988	CD79B antigen [MGI:96431]
256	Cd209b	0.406967284214564	0.516167423629291	CD209b antigen [MGI:1916415]
257	Traf1	0.408575039005231	0.286749210913634	TNF receptor-associated factor 1 [MGI:101836]
258	Irf4	0.412691189353366	0.274935864689247	interferon regulatory factor 4 [MGI:1096873]
259	Tlr7	0.416701084665449	0.14659783562647	toll-like receptor 7 [MGI:2176882]
260	Tnfaip8l2	0.418117826063772	0.238942464908366	tumor necrosis factor%2C alpha-induced protein 8-like 2 [MGI:1917019]
261	Cd160	0.438008339817651	0.589499747759476	CD160 antigen [MGI:1860383]

No	GeneSymbol	log2FoldChange	pvalue	Description
262	Cd83	0.439323778072355	0.115022591202222	CD83 antigen [MGI:1328316]
263	Cd180	0.446213810611807	0.0767026097841315	CD180 antigen [MGI:1194924]
264	Cd27	0.447062877049395	0.390775453333307	CD27 antigen [MGI:88326]
265	Cd80	0.451130141529966	0.552967109660943	CD80 antigen [MGI:101775]
266	Il22ra1	0.452030462980702	0.49215470451408	interleukin 22 receptor%2C alpha 1 [MGI:2663588]
267	Ifi27l2b	0.457902377052753	0.345945470886309	interferon%2C alpha-inducible protein 27 like 2B [MGI:1916390]
268	Tlr13	0.458943881899901	0.140577303803213	toll-like receptor 13 [MGI:3045213]
269	Il2ra	0.470293492891564	0.368411860707167	interleukin 2 receptor%2C alpha chain [MGI:96549]
270	Tnfsf12	0.470519992178027	0.182643593257936	tumor necrosis factor (ligand) superfamily%2C member 12 [MGI:1196259]
271	Cd28	0.481779316549017	0.235195443118841	CD28 antigen [MGI:88327]
272	Il21r	0.481866976603221	0.109919325989967	interleukin 21 receptor [MGI:1890475]
273	Cd300a	0.485017580724152	0.0676574688060003	CD300A molecule [MGI:2443411]
274	Il1rl2	0.487670618077008	0.627775880128114	interleukin 1 receptor-like 2 [MGI:1913107]
275	Ifi202b	0.493439585157298	0.128408240461472	interferon activated gene 202B [MGI:1347083]
276	Ccr6	0.500387193255969	0.593849218922839	chemokine (C-C motif) receptor 6 [MGI:1333797]
277	Cd19	0.503283160719574	0.0534139607651807	CD19 antigen [MGI:88319]
278	Tnfrsf1a	0.505749176745495	0.00299045911604082	tumor necrosis factor receptor superfamily%2C member 1a [MGI:1314884]
279	Cd53	0.515837379640613	0.0117913875661492	CD53 antigen [MGI:88341]
280	Tlr4	0.521961684360146	0.170971295513889	toll-like receptor 4 [MGI:96824]
281	Cd79a	0.522728497899787	0.265114290335539	CD79A antigen (immunoglobulin-associated alpha) [MGI:101774]
282	Cxcl12	0.525992759736716	0.000259552574958741	chemokine (C-X-C motif) ligand 12 [MGI:103556]
283	Il13ra2	0.533817072380013	0.550174457658057	interleukin 13 receptor%2C alpha 2 [MGI:1277954]
284	Ifi44	0.533846290140054	0.0316870595370626	interferon-induced protein 44 [MGI:2443016]
285	Cd72	0.534006558076675	0.1526178443517	CD72 antigen [MGI:88345]
286	Cd48	0.535767015101577	0.0230293099051064	CD48 antigen [MGI:88339]
287	Irf8	0.536853164584016	0.00148361989343686	interferon regulatory factor 8 [MGI:96395]
288	Il2rg	0.538253141746562	0.051792348201692	interleukin 2 receptor%2C gamma chain [MGI:96551]
289	Cd209d	0.546107262084666	0.636995577542673	CD209d antigen [MGI:2157947]
290	Cd226	0.546392951973788	0.121403527765362	CD226 antigen [MGI:3039602]
291	Cd247	0.558469431605847	0.23918555678342	CD247 antigen [MGI:88334]
292	Cd74	0.571936825164035	0.0100249349757588	CD74 antigen (invariant polypeptide of major histocompatibility complex%2C class II antigen-associated) [MGI:96534]
293	Ifi203	0.582764082925234	0.011066263737456	interferon activated gene 203 [MGI:96428]
294	Ccl21a	0.584366981579454	0.0319029605365772	chemokine (C-C motif) ligand 21A (serine) [MGI:1349183]
295	Tlr1	0.593702163030924	0.143056785778838	toll-like receptor 1 [MGI:1341295]
296	Cd38	0.595644041864618	0.00674699389742657	CD38 antigen [MGI:107474]
297	Cd86	0.603683034557796	0.0469762594738896	CD86 antigen [MGI:101773]
298	Tnfrsf1b	0.606611616981598	0.00290956546035809	tumor necrosis factor receptor superfamily%2C member 1b [MGI:1314883]
299	Cd3g	0.609923477508849	0.114846942572303	CD3 antigen%2C gamma polypeptide [MGI:88333]
300	Il17rb	0.624549092427731	0.0822593937165307	interleukin 17 receptor B [MGI:1355292]
301	Cd300ld	0.62651751199306	0.147014437119913	CD300 molecule like family member d [MGI:2442358]

No	GeneSymbol	log2FoldChange	pvalue	Description
302	Il6ra	0.62745662204919	0.000681174675524113	interleukin 6 receptor%2C alpha [MGI:105304]
303	Tnfsf13	0.629420644028745	0.533743569652573	tumor necrosis factor (ligand) superfamily%2C member 13 [MGI:1916833]
304	Cxcr3	0.63099611437337	0.169258570948703	chemokine (C-X-C motif) receptor 3 [MGI:1277207]
305	Cd6	0.639549874329568	0.0991506099179415	CD6 antigen [MGI:103566]
306	Xcr1	0.640570076664002	0.106016960264015	chemokine (C motif) receptor 1 [MGI:1346338]
307	Cd3d	0.646919833301991	0.115736458574076	CD3 antigen%2C delta polypeptide [MGI:88331]
308	Il17c	0.667259996841466	0.861592352302551	interleukin 17C [MGI:2446486]
309	Irf5	0.672033355003061	0.00604460221835361	interferon regulatory factor 5 [MGI:1350924]
310	Ifitm6	0.68188010042084	0.338875107910184	interferon induced transmembrane protein 6 [MGI:2686976]
311	Tnfrsf13c	0.69783769658362	0.0218976757977497	tumor necrosis factor receptor superfamily%2C member 13c [MGI:1919299]
312	Cd209a	0.698893250065596	0.306370897173838	CD209a antigen [MGI:2157942]
313	Tnfsf13b	0.700879325634683	0.223013742006234	tumor necrosis factor (ligand) superfamily%2C member 13b [MGI:1344376]
314	Tnfrsf18	0.704469468118433	0.238390413277454	tumor necrosis factor receptor superfamily%2C member 18 [MGI:894675]
315	Ccl2	0.714287694168747	0.463025475282119	chemokine (C-C motif) ligand 2 [MGI:98259]
316	Cd14	0.714771319953072	0.0353409118752642	CD14 antigen [MGI:88318]
317	Tlr9	0.721228649560435	0.0242121459409123	toll-like receptor 9 [MGI:1932389]
318	Il16	0.722850602827847	0.00309414116077347	interleukin 16 [MGI:1270855]
319	Cxcr2	0.752092240879032	0.448199005495829	chemokine (C-X-C motif) receptor 2 [MGI:105303]
320	Ifi203-ps	0.770292226826671	0.315213973645857	interferon activated gene 203%2C pseudogene [MGI:3840117]
321	Tnfsf10	0.771286278672198	0.181020826326392	tumor necrosis factor (ligand) superfamily%2C member 10 [MGI:107414]
322	Ccr1	0.77470738561189	0.160248083844902	chemokine (C-C motif) receptor 1 [MGI:104618]
323	Tlr3	0.778277151960211	0.0215435095885121	toll-like receptor 3 [MGI:2156367]
324	Igtp	0.789314396976879	0.356495740840447	interferon gamma induced GTPase [MGI:107729]
325	Ifit3b	0.791296652637032	0.0095310273031995	interferon-induced protein with tetratricopeptide repeats 3B [MGI:3698419]
326	Cxcr5	0.792983360322817	0.0140572225560079	chemokine (C-X-C motif) receptor 5 [MGI:103567]
327	Cxcl17	0.795741953611212	0.845383403523719	chemokine (C-X-C motif) ligand 17 [MGI:2387642]
328	Il13	0.795741953611212	0.845383403523719	interleukin 13 [MGI:96541]
329	Il5	0.795741953611212	0.845383403523719	interleukin 5 [MGI:96557]
330	Cd300ld3	0.795741953611213	0.845383403523719	CD300 molecule like family member D3 [MGI:2687214]
331	Il11	0.795741953611213	0.845383403523719	interleukin 11 [MGI:107613]
332	Il6	0.795741953611213	0.845383403523719	interleukin 6 [MGI:96559]
333	Cd5	0.796461147491995	0.0497530530681747	CD5 antigen [MGI:88340]
334	Cd40	0.797237518225806	0.107811063032872	CD40 antigen [MGI:88336]
335	Tnfaip3	0.799652030105652	0.0115563469347853	tumor necrosis factor%2C alpha-induced protein 3 [MGI:1196377]
336	Ifi47	0.821606644253156	0.229445027029568	interferon gamma inducible protein 47 [MGI:99448]
337	Irak3	0.823742973890325	0.0733313535720141	interleukin-1 receptor-associated kinase 3 [MGI:1921164]
338	Il2rb	0.828317095444588	0.000132595912680144	interleukin 2 receptor%2C beta chain [MGI:96550]
339	Ccl6	0.830449781595352	0.0044527744697818	chemokine (C-C motif) ligand 6 [MGI:98263]
340	Tnfsf8	0.833698958856261	0.249549146314776	tumor necrosis factor (ligand) superfamily%2C member 8 [MGI:88328]
341	Ifng	0.838094825889796	0.574783831600906	interferon gamma [MGI:107656]

No	GeneSymbol	log2FoldChange	pvalue	Description
342	Cd33	0.876413322247743	0.0354573023568116	CD33 antigen [MGI:99440]
343	Ifi207	0.878535350614814	0.00544209624302547	interferon activated gene 207 [MGI:2138302]
344	Cd4	0.946038620355342	0.000271557003981623	CD4 antigen [MGI:88335]
345	Il18bp	0.956570843727043	0.0498410686680807	interleukin 18 binding protein [MGI:1333800]
346	Irf1	0.965993590376363	0.0750485214668104	interferon regulatory factor 1 [MGI:96590]
347	Il1r1	0.96669390579034	0.00101148013019242	interleukin 1 receptor%2C type I [MGI:96545]
348	Tnfrsf13b	0.968066000458808	0.0035258051754074	tumor necrosis factor receptor superfamily%2C member 13b [MGI:1889411]
349	Ifi209	0.972959177442438	0.0757701294025109	interferon activated gene 209 [MGI:2138243]
350	Cd300e	0.976789250331802	0.046327934140017	CD300E molecule [MGI:2387602]
351	Cd244a	0.980513867948435	0.137159882877012	CD244 molecule A [MGI:109294]
352	Ccr2	0.99280427409108	0.000109489595297875	chemokine (C-C motif) receptor 2 [MGI:106185]
353	Il1r2	0.993179435749462	0.243372833738953	interleukin 1 receptor%2C type II [MGI:96546]
354	Cd200r2	NA	NA	Cd200 receptor 2 [MGI:3042847]
355	Cd209c	NA	NA	CD209c antigen [MGI:2157945]
356	Cd300ld2	NA	NA	CD300 molecule like family member D2 [MGI:3649405]
357	Cd55b	NA	NA	CD55 molecule%2C decay accelerating factor for complement B [MGI:104849]
358	Cd70	NA	NA	CD70 antigen [MGI:1195273]
359	Cd9-ps	NA	NA	Cd9 antigen%2C pseudogene [MGI:3645134]
360	Ccl19-ps1	NA	NA	chemokine (C-C motif) ligand 19%2C pseudogene 1 [MGI:1891387]
361	Ccl20	NA	NA	chemokine (C-C motif) ligand 20 [MGI:1329031]
362	Ccl21b	NA	NA	chemokine (C-C motif) ligand 21B (leucine) [MGI:1349182]
363	Ccl21d	NA	NA	chemokine (C-C motif) ligand 21D [MGI:5434896]
364	Ccl26	NA	NA	chemokine (C-C motif) ligand 26 [MGI:3589281]
365	Ccl27b	NA	NA	chemokine (C-C motif) ligand 27b [MGI:1891389]
366	Cxcl15	NA	NA	chemokine (C-X-C motif) ligand 15 [MGI:1339941]
367	Cxcl3	NA	NA	chemokine (C-X-C motif) ligand 3 [MGI:3037818]
368	Ifna1	NA	NA	interferon alpha 1 [MGI:107668]
369	Ifna11	NA	NA	interferon alpha 11 [MGI:109210]
370	Ifna12	NA	NA	interferon alpha 12 [MGI:2676324]
371	Ifna13	NA	NA	interferon alpha 13 [MGI:2667155]
372	Ifna14	NA	NA	interferon alpha 14 [MGI:3641425]
373	Ifna15	NA	NA	interferon alpha 15 [MGI:3649418]
374	Ifna16	NA	NA	interferon alpha 16 [MGI:3649260]
375	Ifna2	NA	NA	interferon alpha 2 [MGI:107666]
376	Ifna4	NA	NA	interferon alpha 4 [MGI:107664]
377	Ifna5	NA	NA	interferon alpha 5 [MGI:107663]
378	Ifna6	NA	NA	interferon alpha 6 [MGI:107662]
379	Ifna7	NA	NA	interferon alpha 7 [MGI:107661]
380	Ifna9	NA	NA	interferon alpha 9 [MGI:107659]
381	Ifnab	NA	NA	interferon alpha B [MGI:1097683]

No	GeneSymbol	log2FoldChange	pvalue	Description
382	Ifna-ps1	NA	NA	interferon alpha gene%2C pseudogene 1 [MGI:107669]
383	Ifnb1	NA	NA	interferon beta 1%2C fibroblast [MGI:107657]
384	Ifne	NA	NA	interferon epsilon [MGI:2667156]
385	Ifnl2	NA	NA	interferon lambda 2 [MGI:3647279]
386	Ifnl3	NA	NA	interferon lambda 3 [MGI:2450574]
387	Ifnz	NA	NA	interferon zeta [MGI:2448469]
388	Il1bos	NA	NA	interleukin 1 beta%2C opposite strand [MGI:3650458]
389	Il1f10	NA	NA	interleukin 1 family%2C member 10 [MGI:2652548]
390	Il1rapl1	NA	NA	interleukin 1 receptor accessory protein-like 1 [MGI:2687319]
391	Il1rapl2	NA	NA	interleukin 1 receptor accessory protein-like 2 [MGI:1913106]
392	Il17a	NA	NA	interleukin 17A [MGI:107364]
393	Il19	NA	NA	interleukin 19 [MGI:1890472]
394	Il20	NA	NA	interleukin 20 [MGI:1890473]
395	Il20ra	NA	NA	interleukin 20 receptor%2C alpha [MGI:3605069]
396	Il22	NA	NA	interleukin 22 [MGI:1355307]
397	Il22b	NA	NA	interleukin 22B [MGI:2151139]
398	Il23a	NA	NA	interleukin 23%2C alpha subunit p19 [MGI:1932410]
399	Il24	NA	NA	interleukin 24 [MGI:2135548]
400	Il25	NA	NA	interleukin 25 [MGI:2155888]
401	Il3	NA	NA	interleukin 3 [MGI:96552]
402	Il31	NA	NA	interleukin 31 [MGI:1923649]
403	Il36a	NA	NA	interleukin 36A [MGI:1859324]
404	Il36b	NA	NA	interleukin 36B [MGI:1916927]
405	Il4i1	NA	NA	interleukin 4 induced 1 [MGI:109552]
406	Il9	NA	NA	interleukin 9 [MGI:96563]
407	Tnfsf18	NA	NA	tumor necrosis factor (ligand) superfamily%2C member 18 [MGI:2673064]
408	Tnfaip6	NA	NA	tumor necrosis factor alpha induced protein 6 [MGI:1195266]
409	Tgif1-ps	NA	NA	TGFB-induced factor homeobox 1%2C pseudogene [MGI:3779880]
410	Tgif2	NA	NA	TGFB-induced factor homeobox 2 [MGI:1915299]
411	Tgif2lx1	NA	NA	TGFB-induced factor homeobox 2-like%2C X-linked 1 [MGI:2387796]
412	Tgif2lx2	NA	NA	TGFB-induced factor homeobox 2-like%2C X-linked 2 [MGI:3800824]
413	Tgif2-ps1	NA	NA	TGFB-induced factor homeobox 2%2C pseudogene 1 [MGI:3649223]

Supplementary Table S3B

No	GeneSymbol	log2FoldChange	pvalue	Description
1	Ifi44l	-484,050,411,850,084	3.41E+06	interferon-induced protein 44 like [MGI:95975]

No	GeneSymbol	log2FoldChange	pvalue	Description
2	Tgif2-ps2	-275,226,346,620,121	1.01E+04	TGFB-induced factor homeobox 2%2C pseudogene 2 [MGI:3805950]
3	Tnfsf18	-259,266,617,951,788	6.77E+04	tumor necrosis factor (ligand) superfamily%2C member 18 [MGI:2673064]
4	Ccr8	-249,257,209,704,091	0.00541197569589675	chemokine (C-C motif) receptor 8 [MGI:1201402]
5	Cd300ld3	-245,102,221,764,792	2.16E-07	CD300 molecule like family member D3 [MGI:2687214]
6	Ifitm5	-242,152,647,283,567	0.000174473229762461	interferon induced transmembrane protein 5 [MGI:1934923]
7	Tiaf2	-235,305,234,027,899	0.00164157499277649	TGF-beta1-induced anti-apoptotic factor 2 [MGI:2651383]
8	Cd209f	-234,546,065,964,854	1.60E-06	CD209f antigen [MGI:1916392]
9	Tnfsf11	-226,140,185,009,885	0.035959516083427	tumor necrosis factor (ligand) superfamily%2C member 11 [MGI:1100089]
10	Ccl1	-224,763,623,519,066	0.0161466172851243	chemokine (C-C motif) ligand 1 [MGI:98258]
11	Cd24a	-206,637,804,322,302	0.00481493938423419	CD24a antigen [MGI:88323]
12	Il9r	-203,511,687,385,823	2.41E+09	interleukin 9 receptor [MGI:96564]
13	Isg20	-182,251,684,952,779	0.00160010702552367	interferon-stimulated protein [MGI:1928895]
14	Il21	-180,585,568,839,724	1.61E-01	interleukin 21 [MGI:1890474]
15	Il1rl1	-175,102,360,589,306	0.0166372331393263	interleukin 1 receptor-like 1 [MGI:98427]
16	Ccl7	-166,827,998,525,313	1.03E+08	chemokine (C-C motif) ligand 7 [MGI:99512]
17	Il4	-122,610,758,200,174	0.000366298038534149	interleukin 4 [MGI:96556]
18	Cd209a	-115,736,055,826,259	0.000112962857041857	CD209a antigen [MGI:2157942]
19	Il13ra2	-115,230,703,194,707	3.35E+07	interleukin 13 receptor%2C alpha 2 [MGI:1277954]
20	Tnfrsf18	-113,450,306,048,663	6.24E-13	tumor necrosis factor receptor superfamily%2C member 18 [MGI:894675]
21	Cxcl5	-112,385,380,828,418	0.0361351883780983	chemokine (C-X-C motif) ligand 5 [MGI:1096868]
22	Il36rn	-107,151,736,343,667	0.00116538584440261	interleukin 36 receptor antagonist [MGI:1859325]
23	Il20rb	-104,987,544,153,665	5.15E+09	interleukin 20 receptor beta [MGI:2143266]
24	Cd300ld2	-102,916,558,903,242	0.000190917126226982	CD300 molecule like family member D2 [MGI:3649405]
25	Cd55b	-102,916,558,903,242	0.000948545222144646	CD55 molecule%2C decay accelerating factor for complement B [MGI:104849]
26	Ccr1l1	-102,916,558,903,242	4.70E+09	chemokine (C-C motif) receptor 1-like 1 [MGI:104617]
27	Il1bos	-102,916,558,903,242	0.0494950401221928	interleukin 1 beta%2C opposite strand [MGI:3650458]
28	Il24	-102,916,558,903,242	0.000492740545165748	interleukin 24 [MGI:2135548]
29	Ccl22	-100,925,309,458,525	0.00190796071483424	chemokine (C-C motif) ligand 22 [MGI:1306779]
30	Il1b	12,103,177,756,933	0.00297030828312564	interleukin 1 beta [MGI:96543]
31	Ifit2	12,612,286,259,366	0.0106584206143329	interferon-induced protein with tetratricopeptide repeats 2 [MGI:99449]
32	Ccr5	101,506,248,042,418	0.00912425126402945	chemokine (C-C motif) receptor 5 [MGI:107182]
33	Irf7	102,038,181,523,038	0.00348237516559394	interferon regulatory factor 7 [MGI:1859212]
34	Tnf	103,259,031,945,545	0.0126044106243899	tumor necrosis factor [MGI:104798]
35	Il17f	103,989,380,545,395	0.0393350272109223	interleukin 17F [MGI:2676631]
36	Cd69	105,306,531,517,425	0.00144538210611988	CD69 antigen [MGI:88343]
37	Il12rb1	107,459,431,393,926	0.0106343630983416	interleukin 12 receptor%2C beta 1 [MGI:104579]
38	Ifi207	110,084,160,991,092	0.0347682995950831	interferon activated gene 207 [MGI:2138302]
39	Tnfrsf11a	110,203,941,880,374	0.0056012613460807	tumor necrosis factor receptor superfamily%2C member 11a%2C NFKB activator [MGI:1314891]
40	Cd300lb	110,785,446,487,232	0.0335544764585564	CD300 molecule like family member B [MGI:2685099]
41	Cmklr1	112,060,460,534,972	0.00197449987609723	chemokine-like receptor 1 [MGI:109603]

No	GeneSymbol	log2FoldChange	pvalue	Description
42	Ifi205	113,653,847,374,474	1.53E+01	interferon activated gene 205 [MGI:101847]
43	Ifi213	113,762,277,569,128	0.00720094925434131	interferon activated gene 213 [MGI:3695276]
44	Ifit3b	114,372,273,423,023	0.000236956507679813	interferon-induced protein with tetratricopeptide repeats 3B [MGI:3698419]
45	Il22ra1	117,660,927,200,449	0.00458709777878779	interleukin 22 receptor%2C alpha 1 [MGI:2663588]
46	Cd163	118,992,373,626,094	7.29E+05	CD163 antigen [MGI:2135946]
47	Ifi44	123,003,622,906,065	0.00579863342357507	interferon-induced protein 44 [MGI:2443016]
48	Tlr8	123,215,009,396,601	8.73E+04	toll-like receptor 8 [MGI:2176887]
49	Ifi206	124,368,945,457,769	0.049835850714925	interferon activated gene 206 [MGI:3646410]
50	Cd274	125,903,451,185,579	0.0474080845076971	CD274 antigen [MGI:1926446]
51	Ifitm7	127,863,067,845,322	0.000180657104134611	interferon induced transmembrane protein 7 [MGI:1921732]
52	Tlr6	128,859,176,462,586	7.51E+03	toll-like receptor 6 [MGI:1341296]
53	Cd300e	129,037,636,815,681	1.66E-03	CD300E molecule [MGI:2387602]
54	Tnfsf15	129,789,562,497,796	0.00135939643248276	tumor necrosis factor (ligand) superfamily%2C member 15 [MGI:2180140]
55	Cxcl10	130,353,423,350,035	1.17E+04	chemokine (C-X-C motif) ligand 10 [MGI:1352450]
56	Ifi203-ps	132,684,938,869,629	3.72E+07	interferon activated gene 203%2C pseudogene [MGI:3840117]
57	Cxcl11	134,485,427,847,557	7.78E+03	chemokine (C-X-C motif) ligand 11 [MGI:1860203]
58	Il22ra2	136,722,159,334,693	0.000114223794106449	interleukin 22 receptor%2C alpha 2 [MGI:2665114]
59	Cd300c	141,814,574,091,256	0.0102459369324697	CD300C molecule [MGI:3032626]
60	Cd300ld	146,838,087,156,572	0.00269449651645808	CD300 molecule like family member d [MGI:2442358]
61	Tnfsfm13	153,967,727,082,311	0.00140840138193554	tumor necrosis factor (ligand) superfamily%2C membrane-bound member 13 [MGI:3845075]
62	Ifi202b	154,567,207,683,445	0.0269931999868927	interferon activated gene 202B [MGI:1347083]
63	Il1rn	155,307,427,802,513	6.72E+09	interleukin 1 receptor antagonist [MGI:96547]
64	Tnip3	157,581,358,248,107	2.51E+08	TNFAIP3 interacting protein 3 [MGI:3041165]
65	Ifit3	162,697,947,386,414	7.57E+09	interferon-induced protein with tetratricopeptide repeats 3 [MGI:1101055]
66	Cd200r4	164,226,301,098,143	0.0123288404931076	CD200 receptor 4 [MGI:3036289]
67	Cxcl3	169,873,919,381,099	0.0240709820180269	chemokine (C-X-C motif) ligand 3 [MGI:3037818]
68	Il31ra	169,873,919,381,099	1.68E+03	interleukin 31 receptor A [MGI:2180511]
69	Ifi27l2b	172,226,380,355,558	0.00134672923325406	interferon%2C alpha-inducible protein 27 like 2B [MGI:1916390]
70	Cd200r2	173,987,013,393,556	4.41E+06	Cd200 receptor 2 [MGI:3042847]
71	Ifi204	174,234,517,155,698	0.00901579846684296	interferon activated gene 204 [MGI:96429]
72	Il18bp	182,819,544,535,121	0.0193064993864519	interleukin 18 binding protein [MGI:1333800]
73	Ccl28	183,296,823,489,948	0.00087067527861451	chemokine (C-C motif) ligand 28 [MGI:1861731]
74	Cd1d2	189,534,233,475,896	0.0341798732138818	CD1d2 antigen [MGI:107675]
75	Tnfsf13os	196,705,766,442,873	0.0002905955302781	tumor necrosis factor (ligand) superfamily%2C member 13%2C opposite strand [MGI:1919587]
76	Ccl3	208,633,154,195,104	1.06E+07	chemokine (C-C motif) ligand 3 [MGI:98260]
77	Cxcl17	212,172,944,315,682	1.97E+08	chemokine (C-X-C motif) ligand 17 [MGI:2387642]
78	Ifi211	228,570,129,943,568	0.0343555049888864	interferon activated gene 211 [MGI:3041120]
79	Il11	245,294,076,014,196	0.00168515114195359	interleukin 11 [MGI:107613]
80	Il17c	246,132,411,893,887	1.31E-34	interleukin 17C [MGI:2446486]
81	Cd46	261,718,379,132,325	1.56E+08	CD46 antigen%2C complement regulatory protein [MGI:1203290]

No	GeneSymbol	log2FoldChange	pvalue	Description
82	Cd207	278,228,146,955,687	3.58E-18	CD207 antigen [MGI:2180021]
83	Cxcl9	298,151,415,515,268	2.79E-47	chemokine (C-X-C motif) ligand 9 [MGI:1352449]
84	Ifng	346,349,453,681,797	0.00205732852765172	interferon gamma [MGI:107656]
85	Ccl8	357,811,639,287,199	4.50E+00	chemokine (C-C motif) ligand 8 [MGI:101878]
86	Cd33	-0.00206894340042602	4.85E+02	CD33 antigen [MGI:99440]
87	Ticam1	-0.00776199401549808	0.976860736957682	toll-like receptor adaptor molecule 1 [MGI:2147032]
88	Cd226	-0.00793817960121456	0.986391712184033	CD226 antigen [MGI:3039602]
89	Ccl6	-0.0134644221181461	0.98000118980861	chemokine (C-C motif) ligand 6 [MGI:98263]
90	Cd2bp2	-0.0209667208931064	0.922260756813839	CD2 cytoplasmic tail binding protein 2 [MGI:1917483]
91	Il18rap	-0.0476326272446964	0.928613766304406	interleukin 18 receptor accessory protein [MGI:1338888]
92	Cd28	-0.0501520252648333	0.912648158680412	CD28 antigen [MGI:88327]
93	Cxcl14	-0.0582544223196329	0.902284860536707	chemokine (C-X-C motif) ligand 14 [MGI:1888514]
94	Cd53	-0.0596368816991226	0.896089327456709	CD53 antigen [MGI:88341]
95	Il7r	-0.0596989949411239	0.900733494062685	interleukin 7 receptor [MGI:96562]
96	Cxcl2	-0.0673813451958187	0.98397949829283	chemokine (C-X-C motif) ligand 2 [MGI:1340094]
97	Tnfaip6	-0.0673813451958187	0.98397949829283	tumor necrosis factor alpha induced protein 6 [MGI:1195266]
98	Cd93	-0.0725845789445186	0.81124398309121	CD93 antigen [MGI:106664]
99	Tgif1	-0.0797241220552671	0.83181015106553	TGFB-induced factor homeobox 1 [MGI:1194497]
100	Cd40	-0.0905646844356355	0.842882117596452	CD40 antigen [MGI:88336]
101	Cd3d	-0.0914454353228171	0.826250765857615	CD3 antigen%2C delta polypeptide [MGI:88331]
102	Ifngr2	-0.0957451234525125	0.75022482368458	interferon gamma receptor 2 [MGI:107654]
103	Cxcr2	-0.0959007774680071	0.930643779034199	chemokine (C-X-C motif) receptor 2 [MGI:105303]
104	Cd47	-0.0975187144232861	0.770490727885801	CD47 antigen (Rh-related antigen%2C integrin-associated signal transducer) [MGI:96617]
105	Tab2	-0.105086053718113	0.654586871313516	TGF-beta activated kinase 1/MAP3K7 binding protein 2 [MGI:1915902]
106	Cd81	-0.111403596285922	0.688358787240081	CD81 antigen [MGI:1096398]
107	Il17d	-0.122028447133116	0.925272084903702	interleukin 17D [MGI:2446510]
108	Cd83	-0.131729279690585	0.741152742072415	CD83 antigen [MGI:1328316]
109	Il6st	-0.135878270719172	0.577731314903669	interleukin 6 signal transducer [MGI:96560]
110	Il2ra	-0.1370753559063	0.810607574845762	interleukin 2 receptor%2C alpha chain [MGI:96549]
111	Irf3	-0.138248485304541	0.503894243874256	interferon regulatory factor 3 [MGI:1859179]
112	Ifngr1	-0.144552757218623	0.669204827144374	interferon gamma receptor 1 [MGI:107655]
113	Il17ra	-0.147371475841095	0.608479472928764	interleukin 17 receptor A [MGI:107399]
114	Cd82	-0.151750644386617	0.558385430726052	CD82 antigen [MGI:104651]
115	Tradd	-0.153653573518962	0.648021234531297	TNFRSF1A-associated via death domain [MGI:109200]
116	Cd3eap	-0.166334689903377	0.68311708465339	CD3E antigen%2C epsilon polypeptide associated protein [MGI:1917583]
117	Irf2bp2	-0.167307514840589	0.592628780573562	interferon regulatory factor 2 binding protein 2 [MGI:2443921]
118	Cd3g	-0.177530196021157	0.726062277838288	CD3 antigen%2C gamma polypeptide [MGI:88333]
119	Il33	-0.199012392586926	0.581505622160395	interleukin 33 [MGI:1924375]
120	Ifnar1	-0.213043569833111	0.384604184793477	interferon (alpha and beta) receptor 1 [MGI:107658]
121	Irak1bp1	-0.213534310299973	0.714765821455163	interleukin-1 receptor-associated kinase 1 binding protein 1 [MGI:1929475]

No	GeneSymbol	log2FoldChange	pvalue	Description
122	Tnfrsf13b	-0.216987627030838	0.645870255206168	tumor necrosis factor receptor superfamily%2C member 13b [MGI:1889411]
123	Traf3	-0.216991837960707	0.486040267012135	TNF receptor-associated factor 3 [MGI:108041]
124	Cd55	-0.225588512763036	0.475021408683729	CD55 molecule%2C decay accelerating factor for complement [MGI:104850]
125	Traf1	-0.226173856801173	0.574448905576599	TNF receptor-associated factor 1 [MGI:101836]
126	Cd27	-0.226350120890811	0.638350564159356	CD27 antigen [MGI:88326]
127	Cd37	-0.240365773746762	0.28951599087779	CD37 antigen [MGI:88330]
128	Il1r1	-0.25035114885921	0.591269441850057	interleukin 1 receptor%2C type I [MGI:96545]
129	Ccl5	-0.25326940542412	0.621116008319578	chemokine (C-C motif) ligand 5 [MGI:98262]
130	Cxcr3	-0.267634869885066	0.626589026674859	chemokine (C-X-C motif) receptor 3 [MGI:1277207]
131	Irf2bp1	-0.268662500678373	0.331080110285017	interferon regulatory factor 2 binding protein 1 [MGI:2442159]
132	Tnfrsf17	-0.273239831043352	0.842982877278942	tumor necrosis factor receptor superfamily%2C member 17 [MGI:1343050]
133	Il16	-0.273379007342896	0.38485346253948	interleukin 16 [MGI:1270855]
134	Cd34	-0.274904490663729	0.354378480948817	CD34 antigen [MGI:88329]
135	Irf2bpl	-0.280811398278624	0.16862494138705	interferon regulatory factor 2 binding protein-like [MGI:2442463]
136	Tnfsf8	-0.284109358348299	0.744625779291833	tumor necrosis factor (ligand) superfamily%2C member 8 [MGI:88328]
137	Tab1	-0.286082297640091	0.359004701665704	TGF-beta activated kinase 1/MAP3K7 binding protein 1 [MGI:1913763]
138	Ifrd1	-0.288729875581826	0.31761880139314	interferon-related developmental regulator 1 [MGI:1316717]
139	Il2rb	-0.294170710276941	0.441821170195249	interleukin 2 receptor%2C beta chain [MGI:96550]
140	Ccr7	-0.300189031845553	0.491621334891789	chemokine (C-C motif) receptor 7 [MGI:103011]
141	Ifit1bl2	-0.306250914074943	0.644073063709782	interferon induced protein with tetratricopeptide repeats 1B like 2 [MGI:2148249]
142	Cd248	-0.30931603785065	0.488131035216912	CD248 antigen%2C endosialin [MGI:1917695]
143	Cxcr5	-0.317744346291985	0.339781623758111	chemokine (C-X-C motif) receptor 5 [MGI:103567]
144	Tab3	-0.340828430103192	0.167918387640697	TGF-beta activated kinase 1/MAP3K7 binding protein 3 [MGI:1913974]
145	Ifitm1	-0.351504600462899	0.463703711758953	interferon induced transmembrane protein 1 [MGI:1915963]
146	Tnfsf13	-0.3531913219406	0.767969914136406	tumor necrosis factor (ligand) superfamily%2C member 13 [MGI:1916833]
147	Traf7	-0.354404143207957	0.243962747393115	TNF receptor-associated factor 7 [MGI:3042141]
148	Traf4	-0.354776233235841	0.145902788082106	TNF receptor associated factor 4 [MGI:1202880]
149	Cd5	-0.366414672330472	0.509964398932403	CD5 antigen [MGI:88340]
150	Cd164l2	-0.368973121207851	0.665988574066049	CD164 sialomucin-like 2 [MGI:1916905]
151	Il11ra1	-0.376571620031636	0.168780101485804	interleukin 11 receptor%2C alpha chain 1 [MGI:107426]
152	Cd1d1	-0.397541152094583	0.312988256973641	CD1d1 antigen [MGI:107674]
153	Tnfaip8l1	-0.404611043775157	0.418202224200525	tumor necrosis factor%2C alpha-induced protein 8-like 1 [MGI:1913693]
154	Traf2	-0.420060597222382	0.282692536467707	TNF receptor-associated factor 2 [MGI:101835]
155	Cd19	-0.422564567738813	0.154592928678183	CD19 antigen [MGI:88319]
156	Cd7	-0.447641556066532	0.448004285696185	CD7 antigen [MGI:88344]
157	Tnfsf9	-0.465066419371445	0.626775070550494	tumor necrosis factor (ligand) superfamily%2C member 9 [MGI:1101058]
158	Il27ra	-0.466774524214885	0.433084162135974	interleukin 27 receptor%2C alpha [MGI:1355318]
159	Cd3e	-0.4747505667399	0.125574139298367	CD3 antigen%2C epsilon polypeptide [MGI:88332]
160	Ifitm6	-0.475270426065061	0.706240452331619	interferon induced transmembrane protein 6 [MGI:2686976]
161	Isg20l2	-0.477014631462374	0.0747837934208667	interferon stimulated exonuclease gene 20-like 2 [MGI:2140076]

No	GeneSymbol	log2FoldChange	pvalue	Description
162	Tnfrsf19	-0.501001972834406	0.510127042847175	tumor necrosis factor receptor superfamily%2C member 19 [MGI:1352474]
163	Cd276	-0.510407044202686	0.411913693606737	CD276 antigen [MGI:2183926]
164	Il1rap	-0.51326333372059	0.150009976554486	interleukin 1 receptor accessory protein [MGI:104975]
165	Cd22	-0.515061306956517	0.0906300279093225	CD22 antigen [MGI:88322]
166	Cd72	-0.550794885805171	0.205164487278066	CD72 antigen [MGI:88345]
167	Cd2	-0.556721259658047	0.157304927935754	CD2 antigen [MGI:88320]
168	Cd79b	-0.557460274514082	0.0449436408434704	CD79B antigen [MGI:96431]
169	Il12a	-0.59935881869844	0.461121049267269	interleukin 12a [MGI:96539]
170	Ccl21a	-0.62490063151664	0.0387577024391111	chemokine (C-C motif) ligand 21A (serine) [MGI:1349183]
171	Cxcr6	-0.635423901019398	0.246146904526919	chemokine (C-X-C motif) receptor 6 [MGI:1934582]
172	Cd177	-0.642889486682101	0.567755011171611	CD177 antigen [MGI:1916141]
173	Cd79a	-0.646303359307361	0.0285458769970964	CD79A antigen (immunoglobulin-associated alpha) [MGI:101774]
174	Tnfrsf13c	-0.665721568393233	0.0720567059126173	tumor necrosis factor receptor superfamily%2C member 13c [MGI:1919299]
175	Ccl27a	-0.681707039535101	0.417509272608422	chemokine (C-C motif) ligand 27A [MGI:1343459]
176	Ccl4	-0.685727309573982	0.539753474640845	chemokine (C-C motif) ligand 4 [MGI:98261]
177	Ccr6	-0.694476215862164	0.619737079952256	chemokine (C-C motif) receptor 6 [MGI:1333797]
178	Cxcl1	-0.700942929627174	0.654887961143336	chemokine (C-X-C motif) ligand 1 [MGI:108068]
179	Irf4	-0.72205017982433	0.161565988847389	interferon regulatory factor 4 [MGI:1096873]
180	Tnfrsf10b	-0.734814660202982	0.28905673637562	tumor necrosis factor receptor superfamily%2C member 10b [MGI:1341090]
181	Cxcr4	-0.74329812575435	0.108228075451336	chemokine (C-X-C motif) receptor 4 [MGI:109563]
182	Il34	-0.747078147721499	0.191002075584726	interleukin 34 [MGI:1923777]
183	Cd6	-0.76222624057118	0.0886634320280111	CD6 antigen [MGI:103566]
184	Ifrd2	-0.774551363074548	0.002758188167617	interferon-related developmental regulator 2 [MGI:1316708]
185	Cd59b	-0.774993782148913	0.412114269120931	CD59b antigen [MGI:1888996]
186	Ccl12	-0.781351127148692	0.730692052657286	chemokine (C-C motif) ligand 12 [MGI:108224]
187	Cd244a	-0.792001778434895	0.254272884477441	CD244 molecule A [MGI:109294]
188	Tnfrsf4	-0.813199125031461	0.329256467700555	tumor necrosis factor receptor superfamily%2C member 4 [MGI:104512]
189	Ccl25	-0.819852239000792	0.116609485135265	chemokine (C-C motif) ligand 25 [MGI:1099448]
190	Tlr5	-0.822244665532003	0.278984012966968	toll-like receptor 5 [MGI:1858171]
191	Tnfrsf12a	-0.834400169261613	0.0470276259055394	tumor necrosis factor receptor superfamily%2C member 12a [MGI:1351484]
192	Cd101	-0.843284005099002	0.56062108426356	CD101 antigen [MGI:2685862]
193	Ccl17	-0.868375100052798	0.619456556363792	chemokine (C-C motif) ligand 17 [MGI:1329039]
194	Tnfrsf8	-0.90152624948967	0.595012668763502	tumor necrosis factor receptor superfamily%2C member 8 [MGI:99908]
195	Cd209b	-0.929341041368995	0.295272710620381	CD209b antigen [MGI:1916415]
196	Tnfsf14	-0.940562116761458	0.162221343437285	tumor necrosis factor (ligand) superfamily%2C member 14 [MGI:1355317]
197	Tnfrsf14	-0.943905408063596	0.138037923609183	tumor necrosis factor receptor superfamily%2C member 14 (herpesvirus entry mediator) [MGI:2675303]
198	Ccr3	-0.944648285264076	0.030108701797512	chemokine (C-C motif) receptor 3 [MGI:104616]
199	Cd200r3	-0.989252484834151	0.585518225819119	CD200 receptor 3 [MGI:1921853]
200	Il17rd	0.00513071661934416	0.99337633602419	interleukin 17 receptor D [MGI:2159727]
201	Il4ra	0.00739254912889015	0.983169465739287	interleukin 4 receptor%2C alpha [MGI:105367]

No	GeneSymbol	log2FoldChange	pvalue	Description
202	Cd59a	0.0102116284127143	0.976010104114609	CD59a antigen [MGI:109177]
203	Cklf	0.0238214701502909	0.960563076130916	chemokine-like factor [MGI:1922708]
204	Irak1	0.026486829481437	0.885228447087486	interleukin-1 receptor-associated kinase 1 [MGI:107420]
205	Cx3cl1	0.0265295155270961	0.91849578472727	chemokine (C-X3-C motif) ligand 1 [MGI:1097153]
206	Cd9	0.027620133039154	0.88881182749348	CD9 antigen [MGI:88348]
207	Ifnar2	0.0277789424426939	0.938050551044685	interferon (alpha and beta) receptor 2 [MGI:1098243]
208	Irf9	0.0290158214364899	0.923173396286491	interferon regulatory factor 9 [MGI:107587]
209	Ilf3	0.0313177736732911	0.913577176347176	interleukin enhancer binding factor 3 [MGI:1339973]
210	Cd44	0.0314990795108843	0.954901455287546	CD44 antigen [MGI:88338]
211	Ilf2	0.0382480175469117	0.894506929752381	interleukin enhancer binding factor 2 [MGI:1915031]
212	Tirap	0.0411132352722402	0.906414663139015	toll-interleukin 1 receptor (TIR) domain-containing adaptor protein [MGI:2152213]
213	Irf2	0.0432815415110392	0.890236767135595	interferon regulatory factor 2 [MGI:96591]
214	Cx3cr1	0.0499006672647942	0.920875013226655	chemokine (C-X3-C motif) receptor 1 [MGI:1333815]
215	Cd99l2	0.0688619278449657	0.787982668090571	CD99 antigen-like 2 [MGI:2177151]
216	Il21r	0.0768406839215812	0.824595483251099	interleukin 21 receptor [MGI:1890475]
217	Irf6	0.0862563907548403	0.772758533576259	interferon regulatory factor 6 [MGI:1859211]
218	Tnip1	0.0862973184078723	0.799608191306511	TNFAIP3 interacting protein 1 [MGI:1926194]
219	Cd8a	0.0863330732448746	0.83367861044911	CD8 antigen%2C alpha chain [MGI:88346]
220	Ifi208	0.101515088829744	0.875714752187647	interferon activated gene 208 [MGI:2442822]
221	Trap1	0.105804771037286	0.693273950926281	TNF receptor-associated protein 1 [MGI:1915265]
222	Traf5	0.107089160712184	0.787431233431759	TNF receptor-associated factor 5 [MGI:107548]
223	Cd52	0.109995915639926	0.788456525738831	CD52 antigen [MGI:1346088]
224	Ifitm10	0.113447376555428	0.903940732331662	interferon induced transmembrane protein 10 [MGI:2444776]
225	Cd302	0.113459950104681	0.764224809209977	CD302 antigen [MGI:1913455]
226	Il10rb	0.113809125001997	0.667825422423451	interleukin 10 receptor%2C beta [MGI:109380]
227	Ccl9	0.12055548778658	0.808037266625029	chemokine (C-C motif) ligand 9 [MGI:104533]
228	Ifitm2	0.125432233233118	0.767097125344831	interferon induced transmembrane protein 2 [MGI:1933382]
229	Il1r2	0.129088850568251	0.958651526042557	interleukin 1 receptor%2C type II [MGI:96546]
230	Cd40lg	0.129876739731585	0.898019102218526	CD40 ligand [MGI:88337]
231	Cd200	0.130466769985956	0.717910457681922	CD200 antigen [MGI:1196990]
232	Traf6	0.131736759795426	0.586205202123286	TNF receptor-associated factor 6 [MGI:108072]
233	Cxcl12	0.131897577372543	0.636476520310303	chemokine (C-X-C motif) ligand 12 [MGI:103556]
234	Tnip2	0.132249687238207	0.763415356735593	TNFAIP3 interacting protein 2 [MGI:2386643]
235	Cd8b1	0.135740657735474	0.770648048898817	CD8 antigen%2C beta chain 1 [MGI:88347]
236	Cd209d	0.138580723875371	0.903519791727598	CD209d antigen [MGI:2157947]
237	Il13ra1	0.149009290423139	0.507221500009348	interleukin 13 receptor%2C alpha 1 [MGI:105052]
238	Cd96	0.152502446058657	0.776605127501405	CD96 antigen [MGI:1934368]
239	Ccl2	0.153529336985823	0.893853227125985	chemokine (C-C motif) ligand 2 [MGI:98259]
240	Il6ra	0.156916638037613	0.70305767374047	interleukin 6 receptor%2C alpha [MGI:105304]
241	Cd151	0.158827464688539	0.52866558889635	CD151 antigen [MGI:1096360]

No	GeneSymbol	log2FoldChange	pvalue	Description
242	Cd84	0.160278477783832	0.717403377285146	CD84 antigen [MGI:1336885]
243	Cd2ap	0.162294234715939	0.43564983956	CD2-associated protein [MGI:1330281]
244	Tnfrsf22	0.186662763039352	0.790656451244977	tumor necrosis factor receptor superfamily%2C member 22 [MGI:1930270]
245	Il1a	0.191026530637798	0.803032437723056	interleukin 1 alpha [MGI:96542]
246	Il10	0.200036963874014	0.861349734901978	interleukin 10 [MGI:96537]
247	Ifi27l2a	0.202974100024806	0.604793311260638	interferon%2C alpha-inducible protein 27 like 2A [MGI:1924183]
248	Ccr1	0.206756003253819	0.804433596149622	chemokine (C-C motif) receptor 1 [MGI:104618]
249	Tnfrsf1a	0.216758344197197	0.568820085273657	tumor necrosis factor receptor superfamily%2C member 1a [MGI:1314884]
250	Cd320	0.216796824267618	0.441663905129712	CD320 antigen [MGI:1860083]
251	Cd300lg	0.219165869267506	0.533750920504978	CD300 molecule like family member G [MGI:1289168]
252	Ccr9	0.226191808043606	0.740601009223195	chemokine (C-C motif) receptor 9 [MGI:1341902]
253	Tnfaip1	0.231173458162557	0.317045061246565	tumor necrosis factor%2C alpha-induced protein 1 (endothelial) [MGI:104961]
254	Tnfaip2	0.260127042564465	0.790594881335235	tumor necrosis factor%2C alpha-induced protein 2 [MGI:104960]
255	Ifi35	0.264990192995765	0.468459597181812	interferon-induced protein 35 [MGI:1917360]
256	Ccrl2	0.268780062209503	0.661049557026141	chemokine (C-C motif) receptor-like 2 [MGI:1920904]
257	Irf8	0.269125923434047	0.5164479892558	interferon regulatory factor 8 [MGI:96395]
258	Tnfrsf25	0.277689055569489	0.850778288101737	tumor necrosis factor receptor superfamily%2C member 25 [MGI:1934667]
259	Cxcl13	0.278198213844974	0.461421348572156	chemokine (C-X-C motif) ligand 13 [MGI:1888499]
260	Ifi27	0.280439639903934	0.313441233522925	interferon%2C alpha-inducible protein 27 [MGI:1277180]
261	Cd63-ps	0.285090156040785	0.495174123252357	CD63 antigen%2C pseudogene [MGI:105972]
262	Cd247	0.286518708570985	0.660736906784767	CD247 antigen [MGI:88334]
263	Cd36	0.297039141349428	0.47347312405646	CD36 molecule [MGI:107899]
264	Cd300a	0.30694788102953	0.738710329519867	CD300A molecule [MGI:2443411]
265	Ifi209	0.307683769336104	0.534199412949758	interferon activated gene 209 [MGI:2138243]
266	Cd80	0.308807063363836	0.726412099052183	CD80 antigen [MGI:101775]
267	Cd63	0.314169500463518	0.463002724859968	CD63 antigen [MGI:99529]
268	Cd86	0.319277438806425	0.452098037383313	CD86 antigen [MGI:101773]
269	Tnfrsf11b	0.320909783291323	0.618793761090844	tumor necrosis factor receptor superfamily%2C member 11b (osteoprotegerin) [MGI:109587]
270	Irak2	0.32185951363442	0.168398294074297	interleukin-1 receptor-associated kinase 2 [MGI:2429603]
271	Il2rg	0.323152175607058	0.522793973111981	interleukin 2 receptor%2C gamma chain [MGI:96551]
272	Ticam2	0.323168581569721	0.748766241641418	toll-like receptor adaptor molecule 2 [MGI:3040056]
273	Irak4	0.334858371752397	0.432925032431114	interleukin-1 receptor-associated kinase 4 [MGI:2182474]
274	Il17rc	0.337011365860883	0.413663287657022	interleukin 17 receptor C [MGI:2159336]
275	Irf5	0.341563089506558	0.431319270945566	interferon regulatory factor 5 [MGI:1350924]
276	Tlr9	0.359433225993958	0.438210116745059	toll-like receptor 9 [MGI:1932389]
277	Cd4	0.360696778002721	0.342583745463306	CD4 antigen [MGI:88335]
278	Tlr11	0.36084038903355	0.729457808946374	toll-like receptor 11 [MGI:3045226]
279	Cxcl16	0.360905208325492	0.409755719309216	chemokine (C-X-C motif) ligand 16 [MGI:1932682]
280	Il18	0.367461207668144	0.39996045817076	interleukin 18 [MGI:107936]
281	Cd38	0.370302109808626	0.475387175559313	CD38 antigen [MGI:107474]

No	GeneSymbol	log2FoldChange	pvalue	Description
282	Il18r1	0.370921546138932	0.564801939770483	interleukin 18 receptor 1 [MGI:105383]
283	Ccr10	0.371721899802993	0.842431419149447	chemokine (C-C motif) receptor 10 [MGI:1096320]
284	Ccl11	0.376497540726267	0.796991146135565	chemokine (C-C motif) ligand 11 [MGI:103576]
285	Il10ra	0.395846678564511	0.407341232648807	interleukin 10 receptor%2C alpha [MGI:96538]
286	Tlr4	0.414603570620502	0.513123315840282	toll-like receptor 4 [MGI:96824]
287	Cd48	0.425811066224103	0.42661165173294	CD48 antigen [MGI:88339]
288	Ccl24	0.44568136391711	0.546478513960056	chemokine (C-C motif) ligand 24 [MGI:1928953]
289	Il7	0.462283316068574	0.413779254495012	interleukin 7 [MGI:96561]
290	Tnfaip8	0.476598114851813	0.159349977018754	tumor necrosis factor%2C alpha-induced protein 8 [MGI:2147191]
291	Il3ra	0.480610921946447	0.588602239660802	interleukin 3 receptor%2C alpha chain [MGI:96553]
292	Tnfsf13b	0.486603326388042	0.442941196616557	tumor necrosis factor (ligand) superfamily%2C member 13b [MGI:1344376]
293	Cd160	0.495277421021092	0.540722679573255	CD160 antigen [MGI:1860383]
294	Cd164	0.496710439218781	0.0396289326584156	CD164 antigen [MGI:1859568]
295	Ifitm3	0.518051945601705	0.297214760905514	interferon induced transmembrane protein 3 [MGI:1913391]
296	Cd300c2	0.518948611837484	0.334547395177006	CD300C molecule 2 [MGI:2153249]
297	Xcr1	0.520060738149727	0.29462674065544	chemokine (C motif) receptor 1 [MGI:1346338]
298	Tnfrsf1b	0.526115663768481	0.339980450880836	tumor necrosis factor receptor superfamily%2C member 1b [MGI:1314883]
299	Il12b	0.534668232978742	0.739536989132326	interleukin 12b [MGI:96540]
300	Tnfrsf9	0.54613525787547	0.585404282771568	tumor necrosis factor receptor superfamily%2C member 9 [MGI:1101059]
301	Ifi30	0.549309759634616	0.245151152483171	interferon gamma inducible protein 30 [MGI:2137648]
302	Ifih1	0.568910147575179	0.230702956149094	interferon induced with helicase C domain 1 [MGI:1918836]
303	Tlr1	0.577677252560117	0.212798127307888	toll-like receptor 1 [MGI:1341295]
304	Tnfaip8l2	0.581854332709144	0.301228893597883	tumor necrosis factor%2C alpha-induced protein 8-like 2 [MGI:1917019]
305	Tnfsf10	0.585075608646759	0.257240643433837	tumor necrosis factor (ligand) superfamily%2C member 10 [MGI:107414]
306	Il15ra	0.590515300125374	0.191111001889493	interleukin 15 receptor%2C alpha chain [MGI:104644]
307	Ccl19-ps3	0.597266702594463	0.799583711791977	chemokine (C-C motif) ligand 19%2C pseudogene 3 [MGI:1891391]
308	Ifi214	0.618898299245183	0.302582353901013	interferon activated gene 214 [MGI:3584522]
309	Cd55os	0.620989017005325	0.58468517665532	CD55 molecule%2C opposite strand sequence [MGI:3783116]
310	Il36g	0.633849083375402	0.64362908428729	interleukin 36G [MGI:2449929]
311	Tlr2	0.646228293480253	0.357737146314946	toll-like receptor 2 [MGI:1346060]
312	Cd5l	0.648623696747702	0.506838322648136	CD5 antigen-like [MGI:1334419]
313	Tnfrsf23	0.658366047324219	0.347739378749646	tumor necrosis factor receptor superfamily%2C member 23 [MGI:1930269]
314	Ifit1bl1	0.673115921690766	0.178581693161335	interferon induced protein with tetratricpeptide repeats 1B like 1 [MGI:3650685]
315	Il17re	0.687493646791673	0.262483225198142	interleukin 17 receptor E [MGI:1889371]
316	Il12rb2	0.694255752399446	0.553894650240591	interleukin 12 receptor%2C beta 2 [MGI:1270861]
317	Cd68	0.706012525921867	0.444077832266292	CD68 antigen [MGI:88342]
318	Ifnk	0.707028552922134	0.610097711845782	interferon kappa [MGI:2683287]
319	Tnfaip3	0.711678512791356	0.136503202427365	tumor necrosis factor%2C alpha-induced protein 3 [MGI:1196377]
320	Xcl1	0.719149525032515	0.551658955809747	chemokine (C motif) ligand 1 [MGI:104593]
321	Il17rb	0.728733847545622	0.0823082183667041	interleukin 17 receptor B [MGI:1355292]

No	GeneSymbol	log2FoldChange	pvalue	Description
322	Cd14	0.736580778818048	NA	CD14 antigen [MGI:88318]
323	Irak3	0.748118738352667	0.259806063876885	interleukin-1 receptor-associated kinase 3 [MGI:1921164]
324	Igtp	0.75257001294549	0.436244305875846	interferon gamma induced GTPase [MGI:107729]
325	Tlr12	0.75347685573786	0.406123994744634	toll-like receptor 12 [MGI:3045221]
326	Il1rl2	0.761647538189894	0.396361836632167	interleukin 1 receptor-like 2 [MGI:1913107]
327	Cd200r1	0.761811704958546	0.404805524641853	CD200 receptor 1 [MGI:1889024]
328	Ifit1	0.769293279682255	0.103999776048812	interferon-induced protein with tetratricopeptide repeats 1 [MGI:99450]
329	Tnfrsf26	0.773408487626486	0.194270828379941	tumor necrosis factor receptor superfamily%2C member 26 [MGI:2651928]
330	Il15	0.774435062438784	0.156612911563868	interleukin 15 [MGI:103014]
331	Tnfrsf21	0.78388522183269	0.0556791543322346	tumor necrosis factor receptor superfamily%2C member 21 [MGI:2151075]
332	Il6	0.784088647651479	0.799601734734318	interleukin 6 [MGI:96559]
333	Il4i1	0.78489690225911	0.793844874361501	interleukin 4 induced 1 [MGI:109552]
334	Ifi203	0.80892999165587	0.0154327206509506	interferon activated gene 203 [MGI:96428]
335	Tnfsf12	0.82275185157462	0.0141268931997885	tumor necrosis factor (ligand) superfamily%2C member 12 [MGI:1196259]
336	Irf1	0.823566729930969	0.336165961331771	interferon regulatory factor 1 [MGI:96590]
337	Ccl19	0.826239875686573	0.086863645609314	chemokine (C-C motif) ligand 19 [MGI:1346316]
338	Tlr13	0.83655053068026	0.405969683434862	toll-like receptor 13 [MGI:3045213]
339	Tlr7	0.866985851711395	0.0348204488472539	toll-like receptor 7 [MGI:2176882]
340	Cd180	0.870181479734588	0.0226654806444274	CD180 antigen [MGI:1194924]
341	Cd300lf	0.876786396663099	0.29538710772403	CD300 molecule like family member F [MGI:2442359]
342	Ccr4	0.880698824367088	0.689411374475049	chemokine (C-C motif) receptor 4 [MGI:107824]
343	Cd209g	0.894387762610069	0.826504160611269	CD209g antigen [MGI:1917442]
344	Cd300ld5	0.894387762610069	0.826504160611269	CD300 molecule like family member D5 [MGI:3702661]
345	Cxcr1	0.894387762610069	0.826504160611269	chemokine (C-X-C motif) receptor 1 [MGI:2448715]
346	Il1rapl1	0.894387762610069	0.826504160611269	interleukin 1 receptor accessory protein-like 1 [MGI:2687319]
347	Il11ra2	0.894387762610069	0.826504160611269	interleukin 11 receptor%2C alpha chain 2 [MGI:109123]
348	Il23r	0.894387762610069	0.826504160611269	interleukin 23 receptor [MGI:2181693]
349	Tnfsf4	0.894387762610069	0.826504160611269	tumor necrosis factor (ligand) superfamily%2C member 4 [MGI:104511]
350	Il5ra	0.925051685831226	0.274124747115368	interleukin 5 receptor%2C alpha [MGI:96558]
351	Tnfaip8l3	0.936493574247947	0.317295567591398	tumor necrosis factor%2C alpha-induced protein 8-like 3 [MGI:2685363]
352	Cd109	0.946313848738431	0.512226569781659	CD109 antigen [MGI:2445221]
353	Cd74	0.94768603089005	0.215407926020065	CD74 antigen (invariant polypeptide of major histocompatibility complex%2C class II antigen-associated) [MGI:96534]
354	Iigp1	0.95105303830093	0.14068039412098	interferon inducible GTPase 1 [MGI:1926259]
355	Ccr2	0.962866303293283	0.383010006397212	chemokine (C-C motif) receptor 2 [MGI:106185]
356	Tlr3	0.978899620049289	0.0145708767837963	toll-like receptor 3 [MGI:2156367]
357	Il27	0.979079743081561	0.505610430223945	interleukin 27 [MGI:2384409]
358	Ifnlr1	0.986982264707977	0.23915438872705	interferon lambda receptor 1 [MGI:2429859]
359	Ifi47	0.988567758252489	0.299119205947221	interferon gamma inducible protein 47 [MGI:99448]
360	Cd209c	NA	NA	CD209c antigen [MGI:2157945]
361	Cd209e	NA	NA	CD209e antigen [MGI:2157948]

No	GeneSymbol	log2FoldChange	pvalue	Description
362	Cd300ld4	NA	NA	CD300 molecule like family member D4 [MGI:3702658]
363	Cd70	NA	NA	CD70 antigen [MGI:1195273]
364	Cd9-ps	NA	NA	Cd9 antigen%2C pseudogene [MGI:3645134]
365	Ccl19-ps1	NA	NA	chemokine (C-C motif) ligand 19%2C pseudogene 1 [MGI:1891387]
366	Ccl20	NA	NA	chemokine (C-C motif) ligand 20 [MGI:1329031]
367	Ccl21b	NA	NA	chemokine (C-C motif) ligand 21B (leucine) [MGI:1349182]
368	Ccl21d	NA	NA	chemokine (C-C motif) ligand 21D [MGI:5434896]
369	Ccl26	NA	NA	chemokine (C-C motif) ligand 26 [MGI:3589281]
370	Ccl27b	NA	NA	chemokine (C-C motif) ligand 27b [MGI:1891389]
371	Cxcl15	NA	NA	chemokine (C-X-C motif) ligand 15 [MGI:1339941]
372	Ifna1	NA	NA	interferon alpha 1 [MGI:107668]
373	Ifna11	NA	NA	interferon alpha 11 [MGI:109210]
374	Ifna12	NA	NA	interferon alpha 12 [MGI:2676324]
375	Ifna13	NA	NA	interferon alpha 13 [MGI:2667155]
376	Ifna14	NA	NA	interferon alpha 14 [MGI:3641425]
377	Ifna15	NA	NA	interferon alpha 15 [MGI:3649418]
378	Ifna16	NA	NA	interferon alpha 16 [MGI:3649260]
379	Ifna2	NA	NA	interferon alpha 2 [MGI:107666]
380	Ifna4	NA	NA	interferon alpha 4 [MGI:107664]
381	Ifna5	NA	NA	interferon alpha 5 [MGI:107663]
382	Ifna6	NA	NA	interferon alpha 6 [MGI:107662]
383	Ifna7	NA	NA	interferon alpha 7 [MGI:107661]
384	Ifna9	NA	NA	interferon alpha 9 [MGI:107659]
385	Ifnab	NA	NA	interferon alpha B [MGI:1097683]
386	Ifna-ps1	NA	NA	interferon alpha gene%2C pseudogene 1 [MGI:107669]
387	Ifnb1	NA	NA	interferon beta 1%2C fibroblast [MGI:107657]
388	Ifne	NA	NA	interferon epsilon [MGI:2667156]
389	Ifnl2	NA	NA	interferon lambda 2 [MGI:3647279]
390	Ifnl3	NA	NA	interferon lambda 3 [MGI:2450574]
391	Ifnz	NA	NA	interferon zeta [MGI:2448469]
392	Il1f10	NA	NA	interleukin 1 family%2C member 10 [MGI:2652548]
393	Il1rapl2	NA	NA	interleukin 1 receptor accessory protein-like 2 [MGI:1913106]
394	Il13	NA	NA	interleukin 13 [MGI:96541]
395	Il17a	NA	NA	interleukin 17A [MGI:107364]
396	Il17b	NA	NA	interleukin 17B [MGI:1928397]
397	Il19	NA	NA	interleukin 19 [MGI:1890472]
398	Il2	NA	NA	interleukin 2 [MGI:96548]
399	Il20	NA	NA	interleukin 20 [MGI:1890473]
400	Il20ra	NA	NA	interleukin 20 receptor%2C alpha [MGI:3605069]
401	Il22	NA	NA	interleukin 22 [MGI:1355307]

No	GeneSymbol	log2FoldChange	pvalue	Description
402	Il22b	NA	NA	interleukin 22B [MGI:2151139]
403	Il23a	NA	NA	interleukin 23%2C alpha subunit p19 [MGI:1932410]
404	Il25	NA	NA	interleukin 25 [MGI:2155888]
405	Il3	NA	NA	interleukin 3 [MGI:96552]
406	Il31	NA	NA	interleukin 31 [MGI:1923649]
407	Il36a	NA	NA	interleukin 36A [MGI:1859324]
408	Il36b	NA	NA	interleukin 36B [MGI:1916927]
409	Il5	NA	NA	interleukin 5 [MGI:96557]
410	Il9	NA	NA	interleukin 9 [MGI:96563]
411	Tgif1-ps	NA	NA	TGFB-induced factor homeobox 1%2C pseudogene [MGI:3779880]
412	Tgif2	NA	NA	TGFB-induced factor homeobox 2 [MGI:1915299]
413	Tgif2lx1	NA	NA	TGFB-induced factor homeobox 2-like%2C X-linked 1 [MGI:2387796]
414	Tgif2lx2	NA	NA	TGFB-induced factor homeobox 2-like%2C X-linked 2 [MGI:3800824]
415	Tgif2-ps1	NA	NA	TGFB-induced factor homeobox 2%2C pseudogene 1 [MGI:3649223]

Supplementary Table S3C

No	GeneSymbol	log2FoldChange	pvalue	Description
1	Cd300ld4	-409,992,462,243,739	1.85E+00	CD300 molecule like family member D4 [MGI:3702658]
2	Il21	-278,132,231,991,798	2.10E+09	interleukin 21 [MGI:1890474]
3	Cd209e	-275,156,098,462,265	0.00335308854523479	CD209e antigen [MGI:2157948]
4	Tgif2-ps2	-258,559,071,286,171	0.000300691960238157	TGFB-induced factor homeobox 2%2C pseudogene 2 [MGI:3805950]
5	Il13ra2	-185,023,661,987,733	8.49E-29	interleukin 13 receptor%2C alpha 2 [MGI:1277954]
6	Il4	-176,771,043,178,717	0.00587373036912097	interleukin 4 [MGI:96556]
7	Ccr1l1	-167,556,359,352,467	4.75E+07	chemokine (C-C motif) receptor 1-like 1 [MGI:104617]
8	Ccl22	-152,192,513,662,698	0.000405311671056544	chemokine (C-C motif) ligand 22 [MGI:1306779]
9	Ccr6	-149,131,418,741,072	3.37E+04	chemokine (C-C motif) receptor 6 [MGI:1333797]
10	Tnfrsf8	-143,600,061,613,556	1.01E+09	tumor necrosis factor receptor superfamily%2C member 8 [MGI:99908]
11	Il23r	-137,075,627,434,067	0.00660521731683372	interleukin 23 receptor [MGI:2181693]
12	Tnfrsf9	-129,353,417,056,352	0.0107731970294349	tumor necrosis factor receptor superfamily%2C member 9 [MGI:1101059]
13	Tnfsf8	-109,496,690,026,791	2.56E+09	tumor necrosis factor (ligand) superfamily%2C member 8 [MGI:88328]
14	Tnfrsf19	-102,516,971,073,293	2.37E+07	tumor necrosis factor receptor superfamily%2C member 19 [MGI:1352474]
15	Cd209a	-22,384,928,030,418	2.20E-58	CD209a antigen [MGI:2157942]
16	Ccl24	-14,243,966,528,479	0.000382346343649331	chemokine (C-C motif) ligand 24 [MGI:1928953]
17	Tlr2	101,307,952,521,908	1.86E+07	toll-like receptor 2 [MGI:1346060]
18	Tnfaip2	102,192,162,448,255	0.0232894217011961	tumor necrosis factor%2C alpha-induced protein 2 [MGI:104960]
19	Ifi204	104,632,502,092,079	0.0307452258855603	interferon activated gene 204 [MGI:96429]
20	Irf7	104,744,836,673,836	2.33E+00	interferon regulatory factor 7 [MGI:1859212]

No	GeneSymbol	log2FoldChange	pvalue	Description
21	Ifi205	104,860,323,791,077	0.000175037308649503	interferon activated gene 205 [MGI:101847]
22	Cd59b	105,788,880,034,178	7.10E+09	CD59b antigen [MGI:1888996]
23	Tlr8	106,872,911,519,219	7.26E+00	toll-like receptor 8 [MGI:2176887]
24	Cd209g	107,464,930,628,652	0.0269895576128606	CD209g antigen [MGI:1917442]
25	Cd300ld5	107,464,930,628,652	5.99E+09	CD300 molecule like family member D5 [MGI:3702661]
26	Cxcl2	107,464,930,628,652	1.16E-38	chemokine (C-X-C motif) ligand 2 [MGI:1340094]
27	Ifi44l	107,464,930,628,652	1.63E+02	interferon-induced protein 44 like [MGI:95975]
28	Il1rapl1	107,464,930,628,652	2.09E+05	interleukin 1 receptor accessory protein-like 1 [MGI:2687319]
29	Il11ra2	107,464,930,628,652	0.000492740545165748	interleukin 11 receptor%2C alpha chain 2 [MGI:109123]
30	Tnfsf4	107,464,930,628,652	0.000435017875268627	tumor necrosis factor (ligand) superfamily%2C member 4 [MGI:104511]
31	Tnfaip6	107,464,930,628,652	0.000928373184333609	tumor necrosis factor alpha induced protein 6 [MGI:1195266]
32	Il12b	107,665,581,283,575	0.00874689609902386	interleukin 12b [MGI:96540]
33	Ccl8	108,005,364,655,727	2.58E-03	chemokine (C-C motif) ligand 8 [MGI:101878]
34	Tlr6	110,473,157,865,015	9.46E+09	toll-like receptor 6 [MGI:1341296]
35	Tnfrsf23	111,507,117,297,136	1.08E+00	tumor necrosis factor receptor superfamily%2C member 23 [MGI:1930269]
36	Tnfsf13os	113,219,294,110,661	0.0156911201266431	tumor necrosis factor (ligand) superfamily%2C member 13%2C opposite strand [MGI:1919587]
37	Tlr13	115,547,341,421,694	1.05E+08	toll-like receptor 13 [MGI:3045213]
38	Cd300e	116,142,686,461,352	0.00272713485805022	CD300E molecule [MGI:2387602]
39	Ifi44	116,621,788,027,778	3.52E+04	interferon-induced protein 44 [MGI:2443016]
40	Ifit1bl1	118,674,903,809,899	2.95E+08	interferon induced protein with tetratricpeptide repeats 1B like 1 [MGI:3650685]
41	Ifit2	118,785,956,463,145	8.70E+05	interferon-induced protein with tetratricopeptide repeats 2 [MGI:99449]
42	Il9r	119,277,456,645,938	3.43E+09	interleukin 9 receptor [MGI:96564]
43	Ifit1	120,926,818,238,388	4.45E+00	interferon-induced protein with tetratricopeptide repeats 1 [MGI:99450]
44	Il18bp	121,778,142,923,443	0.0321899239374584	interleukin 18 binding protein [MGI:1333800]
45	Ifi211	122,826,655,903,682	7.81E+07	interferon activated gene 211 [MGI:3041120]
46	Ifi202b	124,045,377,940,071	2.90E+04	interferon activated gene 202B [MGI:1347083]
47	Ifi27l2b	125,657,187,311,355	0.000103171301877075	interferon%2C alpha-inducible protein 27 like 2B [MGI:1916390]
48	Ifit3	130,305,503,452,759	0.0025536018126923	interferon-induced protein with tetratricopeptide repeats 3 [MGI:1101055]
49	Cxcl9	131,528,690,326,603	2.36E+08	chemokine (C-X-C motif) ligand 9 [MGI:1352449]
50	Il22ra2	154,359,794,387,995	0.0162435650732066	interleukin 22 receptor%2C alpha 2 [MGI:2665114]
51	Il12rb2	175,400,760,761,824	1.04E-06	interleukin 12 receptor%2C beta 2 [MGI:1270861]
52	Cd207	178,061,945,229,975	5.13E+04	CD207 antigen [MGI:2180021]
53	Il10	183,995,911,848,254	1.15E+03	interleukin 10 [MGI:96537]
54	Cxcl3	187,324,215,869,305	7.61E+01	chemokine (C-X-C motif) ligand 3 [MGI:3037818]
55	Tnip3	187,434,628,829,558	1.65E-08	TNFAIP3 interacting protein 3 [MGI:3041165]
56	Cd200r2	191,817,943,613,238	2.84E+06	Cd200 receptor 2 [MGI:3042847]
57	Il4i1	191,817,943,613,238	0.000237475369019883	interleukin 4 induced 1 [MGI:109552]
58	Cd14	202,641,041,714,651	9.37E+02	CD14 antigen [MGI:88318]
59	Cd1d2	203,405,396,653,538	0.00832009748099911	CD1d2 antigen [MGI:107675]
60	Il1rn	209,036,679,328,658	5.41E+08	interleukin 1 receptor antagonist [MGI:96547]

No	GeneSymbol	log2FoldChange	pvalue	Description
61	Il12rb1	214,328,629,045,949	1.67E+08	interleukin 12 receptor%2C beta 1 [MGI:104579]
62	Cxcl17	230,065,376,966,381	1.37E+09	chemokine (C-X-C motif) ligand 17 [MGI:2387642]
63	Ifnk	234,286,306,906,596	0.142881181304276	interferon kappa [MGI:2683287]
64	Ifitm7	240,523,715,532,405	0.548094718159512	interferon induced transmembrane protein 7 [MGI:1921732]
65	Il11	263,539,363,972,229	0.174428187317255	interleukin 11 [MGI:107613]
66	Cd163	307,964,851,730,233	0.00964409848365247	CD163 antigen [MGI:2135946]
67	Ccl28	326,910,508,634,092	0.0496813409682616	chemokine (C-C motif) ligand 28 [MGI:1861731]
68	Cd200r4	461,113,034,685,918	0.240453497829044	CD200 receptor 4 [MGI:3036289]
69	Il33	-0.00629978217500211	0.98504438453074	interleukin 33 [MGI:1924375]
70	Tnfsfm13	-0.00817466906482611	0.988667066927702	tumor necrosis factor (ligand) superfamily%2C membrane-bound member 13 [MGI:3845075]
71	Cxcl1	-0.0101554945241577	0.989573811348828	chemokine (C-X-C motif) ligand 1 [MGI:108068]
72	Tnfaip3	-0.0111577725280029	0.977192867233086	tumor necrosis factor%2C alpha-induced protein 3 [MGI:1196377]
73	Cd300lg	-0.0118514882372231	0.970836545715984	CD300 molecule like family member G [MGI:1289168]
74	Cd302	-0.01607928149697	0.960766500470576	CD302 antigen [MGI:1913455]
75	Il1rl1	-0.0259767909914303	0.978500089981421	interleukin 1 receptor-like 1 [MGI:98427]
76	Tnfsf10	-0.0261895437367237	0.976031676986724	tumor necrosis factor (ligand) superfamily%2C member 10 [MGI:107414]
77	Ticam1	-0.0263610353273369	0.914689065062169	toll-like receptor adaptor molecule 1 [MGI:2147032]
78	Ifitm1	-0.0288819832140512	0.940062709520083	interferon induced transmembrane protein 1 [MGI:1915963]
79	Tnfsf14	-0.0337413602636778	0.961448746796842	tumor necrosis factor (ligand) superfamily%2C member 14 [MGI:1355317]
80	Cd3eap	-0.0383274668362301	0.921617383746341	CD3E antigen%2C epsilon polypeptide associated protein [MGI:1917583]
81	Cd2ap	-0.0412113202325419	0.803756408069128	CD2-associated protein [MGI:1330281]
82	Ifnar1	-0.0430406375104941	0.843152982599429	interferon (alpha and beta) receptor 1 [MGI:107658]
83	Tnfsf12	-0.0535444291910669	0.876817496308514	tumor necrosis factor (ligand) superfamily%2C member 12 [MGI:1196259]
84	Cd164l2	-0.0542948347698559	0.95137127972888	CD164 sialomucin-like 2 [MGI:1916905]
85	Il17d	-0.0548734726565384	0.966331398846415	interleukin 17D [MGI:2446510]
86	Cd27	-0.0552832870879904	0.909922328300242	CD27 antigen [MGI:88326]
87	Tnfrsf14	-0.0597458637482882	0.921080600125657	tumor necrosis factor receptor superfamily%2C member 14 (herpesvirus entry mediator) [MGI:2675303]
88	Cd200	-0.065888620135081	0.826380971074643	CD200 antigen [MGI:1196990]
89	Ilf2	-0.0733800908605041	0.758028519621434	interleukin enhancer binding factor 2 [MGI:1915031]
90	Il12a	-0.0753512095241331	0.938950560888775	interleukin 12a [MGI:96539]
91	Traf6	-0.0776167517949854	0.731896982421353	TNF receptor-associated factor 6 [MGI:108072]
92	Cd53	-0.0793009041862201	0.834350886548877	CD53 antigen [MGI:88341]
93	Cd8b1	-0.0797416789623197	0.872423932430793	CD8 antigen%2C beta chain 1 [MGI:88347]
94	Cd209b	-0.0867055620553623	0.919186918714411	CD209b antigen [MGI:1916415]
95	Cd9	-0.0918105137012682	0.654062630876517	CD9 antigen [MGI:88348]
96	Ilf3	-0.0946682120382127	0.688968896484248	interleukin enhancer binding factor 3 [MGI:1339973]
97	Il1r1	-0.0986314902781243	0.815269532352998	interleukin 1 receptor%2C type I [MGI:96545]
98	Tnf	-0.104494758922266	0.923921886421324	tumor necrosis factor [MGI:104798]
99	Cd2	-0.106099528538717	0.785411977014756	CD2 antigen [MGI:88320]
100	Ccl5	-0.109828757602888	0.763232761224308	chemokine (C-C motif) ligand 5 [MGI:98262]

No	GeneSymbol	log2FoldChange	pvalue	Description
101	Cd80	-0.115464530700793	0.89085285016756	CD80 antigen [MGI:101775]
102	Cd40	-0.116641048703766	0.798681060916854	CD40 antigen [MGI:88336]
103	Ifi213	-0.11690609119356	0.93929241234457	interferon activated gene 213 [MGI:3695276]
104	Tnip2	-0.117534016031347	0.770990485076895	TNFAIP3 interacting protein 2 [MGI:2386643]
105	Il10rb	-0.127766273894928	0.598902819763916	interleukin 10 receptor%2C beta [MGI:109380]
106	Irf2bp1	-0.139019441355806	0.527555105504382	interferon regulatory factor 2 binding protein 1 [MGI:2442159]
107	Cx3cl1	-0.146769626344419	0.570606646114902	chemokine (C-X3-C motif) ligand 1 [MGI:1097153]
108	Il7r	-0.150698958656173	0.73780887368926	interleukin 7 receptor [MGI:96562]
109	Ccr7	-0.15458459142029	0.696742247954295	chemokine (C-C motif) receptor 7 [MGI:103011]
110	Il6ra	-0.155144881866079	0.612522966622691	interleukin 6 receptor%2C alpha [MGI:105304]
111	Cxcl12	-0.169257367339871	0.444737856745629	chemokine (C-X-C motif) ligand 12 [MGI:103556]
112	Cd28	-0.181079619401626	0.660039181370002	CD28 antigen [MGI:88327]
113	Il22ra1	-0.183574474778835	0.80153624581063	interleukin 22 receptor%2C alpha 1 [MGI:2663588]
114	Cd4	-0.184882487189952	0.580026358786321	CD4 antigen [MGI:88335]
115	Il21r	-0.185138616008279	0.514824936335132	interleukin 21 receptor [MGI:1890475]
116	Ccl4	-0.193564733552129	0.864906729727093	chemokine (C-C motif) ligand 4 [MGI:98261]
117	Il18r1	-0.193914389550147	0.752024644594228	interleukin 18 receptor 1 [MGI:105383]
118	Ifngr1	-0.196405638574333	0.543758858570337	interferon gamma receptor 1 [MGI:107655]
119	Cd3e	-0.198202920139887	0.574126576200893	CD3 antigen%2C epsilon polypeptide [MGI:88332]
120	Cd226	-0.213313460706133	0.65764349447032	CD226 antigen [MGI:3039602]
121	Traf2	-0.216777829319608	0.554901227358144	TNF receptor-associated factor 2 [MGI:101835]
122	Tnfsf13b	-0.218013054064485	0.709049808140353	tumor necrosis factor (ligand) superfamily%2C member 13b [MGI:1344376]
123	Irf2bp2	-0.221676408632738	0.369273312320874	interferon regulatory factor 2 binding protein 2 [MGI:2443921]
124	Il6st	-0.225478533735707	0.26329072231112	interleukin 6 signal transducer [MGI:96560]
125	Ccl3	-0.236609322152507	0.885860916486732	chemokine (C-C motif) ligand 3 [MGI:98260]
126	Tnfsf9	-0.238228621981986	0.80962490349036	tumor necrosis factor (ligand) superfamily%2C member 9 [MGI:1101058]
127	Cd55	-0.239700983210784	0.467001666365307	CD55 molecule%2C decay accelerating factor for complement [MGI:104850]
128	Traf7	-0.251513618682982	0.267574440875536	TNF receptor-associated factor 7 [MGI:3042141]
129	Cd8a	-0.251592264980369	0.557701321712295	CD8 antigen%2C alpha chain [MGI:88346]
130	Il11ra1	-0.260378755888444	0.2923129574363	interleukin 11 receptor%2C alpha chain 1 [MGI:107426]
131	Ccr3	-0.266581944608793	0.554873843850703	chemokine (C-C motif) receptor 3 [MGI:104616]
132	Tnfrsf4	-0.271610994921636	0.744929477823971	tumor necrosis factor receptor superfamily%2C member 4 [MGI:104512]
133	Ifrd1	-0.273821012558169	0.307265979624789	interferon-related developmental regulator 1 [MGI:1316717]
134	Il27ra	-0.285733043315535	0.543494146663859	interleukin 27 receptor%2C alpha [MGI:1355318]
135	Tnfrsf26	-0.29497211485063	0.55224242338832	tumor necrosis factor receptor superfamily%2C member 26 [MGI:2651928]
136	Tirap	-0.299711310248086	0.304476610288002	toll-interleukin 1 receptor (TIR) domain-containing adaptor protein [MGI:2152213]
137	Il16	-0.300053967192227	0.309236920571565	interleukin 16 [MGI:1270855]
138	Iigp1	-0.300555307508189	0.793968990591732	interferon inducible GTPase 1 [MGI:1926259]
139	Il1rap	-0.303004158328193	0.158463453421858	interleukin 1 receptor accessory protein [MGI:104975]
140	Cd40lg	-0.310309212924606	0.718641149711387	CD40 ligand [MGI:88337]

No	GeneSymbol	log2FoldChange	pvalue	Description
141	Cd37	-0.317952925748662	0.0966132917842814	CD37 antigen [MGI:88330]
142	Irf2bpl	-0.324749578709531	0.0975055346157033	interferon regulatory factor 2 binding protein-like [MGI:2442463]
143	Il18rap	-0.354516213184245	0.469879643677596	interleukin 18 receptor accessory protein [MGI:1338888]
144	Ifitm10	-0.358972777745122	0.669985945727926	interferon induced transmembrane protein 10 [MGI:2444776]
145	Tlr11	-0.372743081176829	0.673967236319704	toll-like receptor 11 [MGI:3045226]
146	Traf3	-0.373492275552831	0.186867031441345	TNF receptor-associated factor 3 [MGI:108041]
147	Cxcr5	-0.375303014663837	0.259523145838491	chemokine (C-X-C motif) receptor 5 [MGI:103567]
148	Cd209f	-0.381010630790393	0.861824245165159	CD209f antigen [MGI:1916392]
149	Cd83	-0.381760488880864	0.263611182572541	CD83 antigen [MGI:1328316]
150	Tiaf2	-0.386392983122796	0.876080302008762	TGF-beta1-induced anti-apoptotic factor 2 [MGI:2651383]
151	Cd34	-0.388622664167885	0.181069763661684	CD34 antigen [MGI:88329]
152	Ccl11	-0.411088143679874	0.75790833309848	chemokine (C-C motif) ligand 11 [MGI:103576]
153	Cxcr6	-0.411135118697785	0.446433281145678	chemokine (C-X-C motif) receptor 6 [MGI:1934582]
154	Cd109	-0.416598686364217	0.761056249986935	CD109 antigen [MGI:2445221]
155	Ccl21a	-0.41937661386809	0.215554397477196	chemokine (C-C motif) ligand 21A (serine) [MGI:1349183]
156	Il2ra	-0.428460914802252	0.39897735589167	interleukin 2 receptor%2C alpha chain [MGI:96549]
157	Il2rb	-0.440182185458623	0.20095560648725	interleukin 2 receptor%2C beta chain [MGI:96550]
158	Cd3g	-0.441203724847344	0.319106335107068	CD3 antigen%2C gamma polypeptide [MGI:88333]
159	Cd5	-0.441779587288103	0.283587474672847	CD5 antigen [MGI:88340]
160	Cd200r3	-0.449047141257079	0.837717455079944	CD200 receptor 3 [MGI:1921853]
161	Ccl6	-0.453547332093083	0.56262185599468	chemokine (C-C motif) ligand 6 [MGI:98263]
162	Ccl25	-0.457700226668724	0.357719413822164	chemokine (C-C motif) ligand 25 [MGI:1099448]
163	Cxcr3	-0.462087798561092	0.344872466167229	chemokine (C-X-C motif) receptor 3 [MGI:1277207]
164	Cd248	-0.470560836798528	0.267708532347523	CD248 antigen%2C endosialin [MGI:1917695]
165	Isg20l2	-0.471818400009623	0.0633270412625933	interferon stimulated exonuclease gene 20-like 2 [MGI:2140076]
166	Tnfrsf12a	-0.519615000335774	0.11012196139068	tumor necrosis factor receptor superfamily%2C member 12a [MGI:1351484]
167	Cxcr4	-0.555442237390704	0.17364542410023	chemokine (C-X-C motif) receptor 4 [MGI:109563]
168	Tnfrsf13b	-0.558544783469858	0.0973502037842684	tumor necrosis factor receptor superfamily%2C member 13b [MGI:1889411]
169	Cd33	-0.569275930699256	0.220090149317365	CD33 antigen [MGI:99440]
170	Il31ra	-0.575352703656152	0.816626339345224	interleukin 31 receptor A [MGI:2180511]
171	Irak1bp1	-0.577622951133223	0.299484219963104	interleukin-1 receptor-associated kinase 1 binding protein 1 [MGI:1929475]
172	Ccl17	-0.599634714933656	0.719733054708835	chemokine (C-C motif) ligand 17 [MGI:1329039]
173	Ifit1bl2	-0.602571359173614	0.379204728456064	interferon induced protein with tetratricopeptide repeats 1B like 2 [MGI:2148249]
174	Cd72	-0.607496397043129	0.186155930976888	CD72 antigen [MGI:88345]
175	Cd19	-0.617819447152275	0.00599629604221468	CD19 antigen [MGI:88319]
176	Tnfrsf10b	-0.627869054217611	0.471692001992258	tumor necrosis factor receptor superfamily%2C member 10b [MGI:1341090]
177	Tnfsf11	-0.629584202702985	0.82132178703679	tumor necrosis factor (ligand) superfamily%2C member 11 [MGI:1100089]
178	Cd79b	-0.633370047990933	0.00403482152776518	CD79B antigen [MGI:96431]
179	Cd79a	-0.639689285205689	0.0129361315170776	CD79A antigen (immunoglobulin-associated alpha) [MGI:101774]
180	Il20rb	-0.655200241119215	0.79936626247078	interleukin 20 receptor beta [MGI:2143266]

No	GeneSymbol	log2FoldChange	pvalue	Description
181	Tnfrsf18	-0.670410054931381	0.264520681059559	tumor necrosis factor receptor superfamily%2C member 18 [MGI:894675]
182	Cd6	-0.677896022160922	0.119618547277613	CD6 antigen [MGI:103566]
183	Il17rd	-0.689325031153535	0.2216562956147	interleukin 17 receptor D [MGI:2159727]
184	Cd244a	-0.693255394507716	0.332538968020667	CD244 molecule A [MGI:109294]
185	Cxcr1	-0.717777590718248	0.835643719326519	chemokine (C-X-C motif) receptor 1 [MGI:2448715]
186	Tnfsf13	-0.750761241816689	0.483120266240697	tumor necrosis factor (ligand) superfamily%2C member 13 [MGI:1916833]
187	Cd177	-0.755706956263262	0.501422566657761	CD177 antigen [MGI:1916141]
188	Ccr4	-0.760400798645006	0.610170021009188	chemokine (C-C motif) receptor 4 [MGI:107824]
189	Tnfrsf25	-0.770035667917447	0.498586303303577	tumor necrosis factor receptor superfamily%2C member 25 [MGI:1934667]
190	Cd22	-0.80299811508737	0.00192984324178873	CD22 antigen [MGI:88322]
191	Irf4	-0.818149468017431	0.0548832319553881	interferon regulatory factor 4 [MGI:1096873]
192	Ccr8	-0.848904045355965	0.835197262016943	chemokine (C-C motif) receptor 8 [MGI:1201402]
193	Ifitm5	-0.848904045355965	0.835197262016943	interferon induced transmembrane protein 5 [MGI:1934923]
194	Il13	-0.848904045355965	0.835197262016943	interleukin 13 [MGI:96541]
195	Il17b	-0.848904045355965	0.835197262016943	interleukin 17B [MGI:1928397]
196	Il2	-0.848904045355965	0.835197262016943	interleukin 2 [MGI:96548]
197	Il5	-0.848904045355965	0.835197262016943	interleukin 5 [MGI:96557]
198	Ccl12	-0.886304743586552	0.701328271597135	chemokine (C-C motif) ligand 12 [MGI:108224]
199	Tnfrsf13c	-0.913002998914999	0.00120791088078383	tumor necrosis factor receptor superfamily%2C member 13c [MGI:1919299]
200	Ccr10	-0.930154799719504	0.514402775698829	chemokine (C-C motif) receptor 10 [MGI:1096320]
201	Tnfrsf11b	0.000802633292793072	0.998975007130411	tumor necrosis factor receptor superfamily%2C member 11b (osteoprotegerin) [MGI:109587]
202	Traf4	0.00272390597007002	0.990206336811233	TNF receptor associated factor 4 [MGI:1202880]
203	Cd2bp2	0.00902630035334023	0.965500254590719	CD2 cytoplasmic tail binding protein 2 [MGI:1917483]
204	Il7	0.0103847352566463	0.983568281409114	interleukin 7 [MGI:96561]
205	Ifitm6	0.0141311816504759	0.990339661483158	interferon induced transmembrane protein 6 [MGI:2686976]
206	Xcl1	0.0166752888139125	0.986900150094642	chemokine (C motif) ligand 1 [MGI:104593]
207	Cd1d1	0.0276119446964711	0.925870679472257	CD1d1 antigen [MGI:107674]
208	Il4ra	0.029325264385659	0.932426353923256	interleukin 4 receptor%2C alpha [MGI:105367]
209	Il13ra1	0.0300082427467002	0.892392664563753	interleukin 13 receptor%2C alpha 1 [MGI:105052]
210	Il34	0.0477939724934537	0.929612934941804	interleukin 34 [MGI:1923777]
211	Ccl19-ps3	0.0517073980438877	0.980024802672649	chemokine (C-C motif) ligand 19%2C pseudogene 3 [MGI:1891391]
212	Il17rb	0.0562227150820265	0.883310367582693	interleukin 17 receptor B [MGI:1355292]
213	Cd99l2	0.0571697896829738	0.81458434545839	CD99 antigen-like 2 [MGI:2177151]
214	Ifi209	0.0599248792133154	0.890312973442159	interferon activated gene 209 [MGI:2138243]
215	Ifrd2	0.0633501278668049	0.751943143061282	interferon-related developmental regulator 2 [MGI:1316708]
216	Cd48	0.0683313994499046	0.876091784312326	CD48 antigen [MGI:88339]
217	Trap1	0.0752175181443699	0.743427003428461	TNF receptor-associated protein 1 [MGI:1915265]
218	Ccl9	0.0769120897199726	0.908971634892831	chemokine (C-C motif) ligand 9 [MGI:104533]
219	Tab2	0.0816244837997741	0.698629652952474	TGF-beta activated kinase 1/MAP3K7 binding protein 2 [MGI:1915902]
220	Ifngr2	0.0851409421457327	0.750790863833649	interferon gamma receptor 2 [MGI:107654]

No	GeneSymbol	log2FoldChange	pvalue	Description
221	Cd93	0.085634716256371	0.766264610631491	CD93 antigen [MGI:106664]
222	Cd151	0.0865241826873063	0.716203908729262	CD151 antigen [MGI:1096360]
223	Igtp	0.0896739291158092	0.937755718583517	interferon gamma induced GTPase [MGI:107729]
224	Ccr9	0.0898644755541599	0.899368707108787	chemokine (C-C motif) receptor 9 [MGI:1341902]
225	Traf1	0.100856137121042	0.79848052646407	TNF receptor-associated factor 1 [MGI:101836]
226	Cd84	0.100934837750633	0.79596790587658	CD84 antigen [MGI:1336885]
227	Ccl7	0.102090312951765	0.964631123985084	chemokine (C-C motif) ligand 7 [MGI:99512]
228	Irf2	0.102552929990441	0.691409428110806	interferon regulatory factor 2 [MGI:96591]
229	Cx3cr1	0.10332451694409	0.818039254968363	chemokine (C-X3-C motif) receptor 1 [MGI:1333815]
230	Cd160	0.10960089576852	0.882427050741873	CD160 antigen [MGI:1860383]
231	Il36rn	0.112876634072893	0.977931224692451	interleukin 36 receptor antagonist [MGI:1859325]
232	Cd300ld3	0.112878262750551	0.97660107865223	CD300 molecule like family member D3 [MGI:2687214]
233	Tnfrsf1a	0.116723847768239	0.74342544794756	tumor necrosis factor receptor superfamily%2C member 1a [MGI:1314884]
234	Il1a	0.117805051344263	0.866397425373629	interleukin 1 alpha [MGI:96542]
235	Cd3d	0.120795502009835	0.773819073612692	CD3 antigen%2C delta polypeptide [MGI:88331]
236	Tnfaip8l3	0.122576261057012	0.89261038081675	tumor necrosis factor%2C alpha-induced protein 8-like 3 [MGI:2685363]
237	Il3ra	0.122590295542371	0.879609953477968	interleukin 3 receptor%2C alpha chain [MGI:96553]
238	Il17f	0.127855608042669	0.938479415877557	interleukin 17F [MGI:2676631]
239	Cd276	0.135270626878699	0.828498035501174	CD276 antigen [MGI:2183926]
240	Il17ra	0.138675576678205	0.528666703444255	interleukin 17 receptor A [MGI:107399]
241	Traf5	0.143390992000718	0.638316894216117	TNF receptor-associated factor 5 [MGI:107548]
242	Cd81	0.147056312965707	0.464755279192465	CD81 antigen [MGI:1096398]
243	Cd274	0.156104468095294	0.881066111960396	CD274 antigen [MGI:1926446]
244	Tnfaip1	0.156554791797204	0.429996250419903	tumor necrosis factor%2C alpha-induced protein 1 (endothelial) [MGI:104961]
245	Tlr4	0.162468415300973	0.777790292125042	toll-like receptor 4 [MGI:96824]
246	Irf8	0.16466024390241	0.65648878133411	interferon regulatory factor 8 [MGI:96395]
247	Il1r2	0.172262609245514	0.940386773467695	interleukin 1 receptor%2C type II [MGI:96546]
248	Cd59a	0.174613612950286	0.512909021318775	CD59a antigen [MGI:109177]
249	Irf5	0.176899673050131	0.657070595475548	interferon regulatory factor 5 [MGI:1350924]
250	Cd63	0.179593833594399	0.641752325580921	CD63 antigen [MGI:99529]
251	Il17re	0.179622436452869	0.725585149737307	interleukin 17 receptor E [MGI:1889371]
252	Irf3	0.181633815616392	0.356323591579499	interferon regulatory factor 3 [MGI:1859179]
253	Cd300c	0.18211226277832	0.913682227168607	CD300C molecule [MGI:3032626]
254	Cxcl14	0.187343389216895	0.613013703153836	chemokine (C-X-C motif) ligand 14 [MGI:1888514]
255	Irak1	0.197352718873187	0.263943871849461	interleukin-1 receptor-associated kinase 1 [MGI:107420]
256	Il27	0.20053187737198	0.865861003970836	interleukin 27 [MGI:2384409]
257	Irak2	0.201054394682217	0.346074600898307	interleukin-1 receptor-associated kinase 2 [MGI:2429603]
258	Cd247	0.201772919812177	0.702730661934345	CD247 antigen [MGI:88334]
259	Ccl27a	0.202987711765463	0.814466326321095	chemokine (C-C motif) ligand 27A [MGI:1343459]
260	Tnip1	0.211762723811261	0.49216665930079	TNFAIP3 interacting protein 1 [MGI:1926194]

No	GeneSymbol	log2FoldChange	pvalue	Description
261	Il17rc	0.214023146907419	0.593778977628006	interleukin 17 receptor C [MGI:2159336]
262	Ifi206	0.221291956196688	0.797940263969278	interferon activated gene 206 [MGI:3646410]
263	Ifi35	0.222629323220719	0.537964324884103	interferon-induced protein 35 [MGI:1917360]
264	Cd52	0.224169724482271	0.533577007451219	CD52 antigen [MGI:1346088]
265	Ccl19	0.226289797518027	0.615970417495101	chemokine (C-C motif) ligand 19 [MGI:1346316]
266	Cd38	0.233475038529637	0.735403603440714	CD38 antigen [MGI:107474]
267	Tradd	0.243827343155133	0.472108949518999	TNFRSF1A-associated via death domain [MGI:109200]
268	Cd320	0.245977215627021	0.358841135886861	CD320 antigen [MGI:1860083]
269	Cd209d	0.253564423950459	0.816681674108299	CD209d antigen [MGI:2157947]
270	Irf9	0.257131317958072	0.348308673354994	interferon regulatory factor 9 [MGI:107587]
271	Cd63-ps	0.268438759899135	0.493058643507591	CD63 antigen%2C pseudogene [MGI:105972]
272	Tlr1	0.270531034236844	0.521708351593235	toll-like receptor 1 [MGI:1341295]
273	Tgif1	0.275465788655244	0.385777530434536	TGFB-induced factor homeobox 1 [MGI:1194497]
274	Il1rl2	0.287315044623065	0.748554368523409	interleukin 1 receptor-like 2 [MGI:1913107]
275	Tnfsf15	0.288442616996102	0.600263354214888	tumor necrosis factor (ligand) superfamily%2C member 15 [MGI:2180140]
276	Cd164	0.294820233060877	0.168030926703047	CD164 antigen [MGI:1859568]
277	Cd300a	0.295228256828646	0.744786873780108	CD300A molecule [MGI:2443411]
278	Xcr1	0.302912320065715	0.49637127701386	chemokine (C motif) receptor 1 [MGI:1346338]
279	Cd300lf	0.303912028687229	0.672944847966544	CD300 molecule like family member F [MGI:2442359]
280	Irf6	0.30786588324277	0.336554777447571	interferon regulatory factor 6 [MGI:1859211]
281	Il18	0.308426789848844	0.442879500783312	interleukin 18 [MGI:107936]
282	Tab1	0.314489943636019	0.342049951898743	TGF-beta activated kinase 1/MAP3K7 binding protein 1 [MGI:1913763]
283	Tnfaip8	0.318142563355438	0.220496166843783	tumor necrosis factor%2C alpha-induced protein 8 [MGI:2147191]
284	Il2rg	0.320772825055357	0.653378935880815	interleukin 2 receptor%2C gamma chain [MGI:96551]
285	Cd96	0.324294131046711	0.516505804886922	CD96 antigen [MGI:1934368]
286	Cxcr2	0.331689121261392	0.781063175494958	chemokine (C-X-C motif) receptor 2 [MGI:105303]
287	Irf1	0.332762399835744	0.722540984389655	interferon regulatory factor 1 [MGI:96590]
288	Tnfrsf1b	0.336035411357627	0.668715671681376	tumor necrosis factor receptor superfamily%2C member 1b [MGI:1314883]
289	Il10ra	0.340519186208458	0.453758710302376	interleukin 10 receptor%2C alpha [MGI:96538]
290	Cd44	0.346645047152503	0.637170633814673	CD44 antigen [MGI:88338]
291	Tlr9	0.363934044859796	0.398623383530067	toll-like receptor 9 [MGI:1932389]
292	Cd86	0.368105536038396	0.338605241888879	CD86 antigen [MGI:101773]
293	Tnfrsf21	0.378292743046086	0.321126056475527	tumor necrosis factor receptor superfamily%2C member 21 [MGI:2151075]
294	Irak4	0.381334123959383	0.348310861244116	interleukin-1 receptor-associated kinase 4 [MGI:2182474]
295	Ifi27l2a	0.383796779837755	0.281889939504822	interferon%2C alpha-inducible protein 27 like 2A [MGI:1924183]
296	Cd82	0.384759910451096	0.0261674825873268	CD82 antigen [MGI:104651]
297	Cd47	0.389338253217249	0.202535636559438	CD47 antigen (Rh-related antigen%2C integrin-associated signal transducer) [MGI:96617]
298	Tab3	0.398676057065896	0.102163052491097	TGF-beta activated kinase 1/MAP3K7 binding protein 3 [MGI:1913974]
299	Il36g	0.401039587209503	0.758838888167789	interleukin 36G [MGI:2449929]
300	Ifi27	0.405622614918507	0.101056811882536	interferon%2C alpha-inducible protein 27 [MGI:1277180]

No	GeneSymbol	log2FoldChange	pvalue	Description
301	Ifi208	0.411179904062548	0.452903087458228	interferon activated gene 208 [MGI:2442822]
302	Ifitm2	0.413258970764261	0.236798201938217	interferon induced transmembrane protein 2 [MGI:1933382]
303	Cxcl16	0.420833865268051	0.288701492750494	chemokine (C-X-C motif) ligand 16 [MGI:1932682]
304	Tlr3	0.432754660864114	0.188463951398783	toll-like receptor 3 [MGI:2156367]
305	Tlr5	0.448073316977401	0.561855305277711	toll-like receptor 5 [MGI:1858171]
306	Cd46	0.453487307388179	0.615644555827261	CD46 antigen%2C complement regulatory protein [MGI:1203290]
307	Ifi30	0.465030309488951	0.271670925181171	interferon gamma inducible protein 30 [MGI:2137648]
308	Ifnlr1	0.488921798662259	0.484704580963926	interferon lambda receptor 1 [MGI:2429859]
309	Cd7	0.505671576092954	0.395635652312737	CD7 antigen [MGI:88344]
310	Cd101	0.534282023894083	0.720750854225281	CD101 antigen [MGI:2685862]
311	Cd55os	0.536788387756854	0.637597170333053	CD55 molecule%2C opposite strand sequence [MGI:3783116]
312	Ifnar2	0.538558391634504	0.110129919283211	interferon (alpha and beta) receptor 2 [MGI:1098243]
313	Il15ra	0.548386845898	0.0999837363410759	interleukin 15 receptor%2C alpha chain [MGI:104644]
314	Cklf	0.548404322819854	0.266433467951957	chemokine-like factor [MGI:1922708]
315	Cxcl13	0.562048705749398	0.0725188915487971	chemokine (C-X-C motif) ligand 13 [MGI:1888499]
316	Ifitm3	0.571341750517422	0.159972593435979	interferon induced transmembrane protein 3 [MGI:1913391]
317	Ticam2	0.572049390810405	0.560190265757297	toll-like receptor adaptor molecule 2 [MGI:3040056]
318	Il15	0.582757356460273	0.195581432573609	interleukin 15 [MGI:103014]
319	Ifi47	0.584967090192526	0.577613063771245	interferon gamma inducible protein 47 [MGI:99448]
320	Cd300lb	0.585760687680359	0.451456524359781	CD300 molecule like family member B [MGI:2685099]
321	Ifi203	0.589469501559503	0.033640040996126	interferon activated gene 203 [MGI:96428]
322	Cd24a	0.595494661440941	0.123032160175617	CD24a antigen [MGI:88323]
323	Cd180	0.61290950320405	0.0759476007481163	CD180 antigen [MGI:1194924]
324	Cxcl5	0.62685079178505	0.601609740621458	chemokine (C-X-C motif) ligand 5 [MGI:1096868]
325	Tnfrsf22	0.639831498742774	0.403911827832648	tumor necrosis factor receptor superfamily%2C member 22 [MGI:1930270]
326	Cd200r1	0.642960795315042	0.422457000481489	CD200 receptor 1 [MGI:1889024]
327	Cxcl10	0.66050248011595	0.474458043468451	chemokine (C-X-C motif) ligand 10 [MGI:1352450]
328	Ifih1	0.664031570289563	0.32247451960126	interferon induced with helicase C domain 1 [MGI:1918836]
329	Tlr7	0.672649820578578	0.0882243701935452	toll-like receptor 7 [MGI:2176882]
330	Ccr1	0.679579322234964	0.365082108987487	chemokine (C-C motif) receptor 1 [MGI:104618]
331	Tlr12	0.695080083221509	0.610482009387906	toll-like receptor 12 [MGI:3045221]
332	Cd300c2	0.703524259197462	0.15718719306917	CD300C molecule 2 [MGI:2153249]
333	Ccrl2	0.710603156943402	0.215251562334509	chemokine (C-C motif) receptor-like 2 [MGI:1920904]
334	Tnfaip8l2	0.711950936128224	0.194356611483864	tumor necrosis factor%2C alpha-induced protein 8-like 2 [MGI:1917019]
335	Ifit3b	0.713753425774395	0.104972636687951	interferon-induced protein with tetratricopeptide repeats 3B [MGI:3698419]
336	Cd69	0.719788895059801	0.181781893206846	CD69 antigen [MGI:88343]
337	Irak3	0.754542200926711	0.234802700954121	interleukin-1 receptor-associated kinase 3 [MGI:1921164]
338	Ifi207	0.757054880988353	0.382431314723057	interferon activated gene 207 [MGI:2138302]
339	Ifng	0.758034795795177	0.48261726444794	interferon gamma [MGI:107656]
340	Ccl2	0.763785046508935	0.495336819864714	chemokine (C-C motif) ligand 2 [MGI:98259]

No	GeneSymbol	log2FoldChange	pvalue	Description
341	Tnfaip8l1	0.768695123503555	0.106239354572966	tumor necrosis factor%2C alpha-induced protein 8-like 1 [MGI:1913693]
342	Ccr2	0.779159167227943	0.45385222359535	chemokine (C-C motif) receptor 2 [MGI:106185]
343	Cd300ld	0.783943906819671	0.434458130190962	CD300 molecule like family member d [MGI:2442358]
344	Cmklr1	0.789175435376237	0.429860406539231	chemokine-like receptor 1 [MGI:109603]
345	Ccr5	0.800429275833292	0.484457485301858	chemokine (C-C motif) receptor 5 [MGI:107182]
346	Tnfrsf11a	0.820343175539459	0.162248683478097	tumor necrosis factor receptor superfamily%2C member 11a%2C NFKB activator [MGI:1314891]
347	Il17c	0.828129553116289	0.73032221610689	interleukin 17C [MGI:2446486]
348	Il1b	0.856356691151241	0.275681105723864	interleukin 1 beta [MGI:96543]
349	Cd36	0.87942269789016	0.0257300903251748	CD36 molecule [MGI:107899]
350	Cd68	0.883532975068716	0.336474036004727	CD68 antigen [MGI:88342]
351	Ifi214	0.887011548556684	0.0795267973564031	interferon activated gene 214 [MGI:3584522]
352	Isg20	0.899666330216774	0.265962566090453	interferon-stimulated protein [MGI:1928895]
353	Cd5l	0.905620291997585	0.369006153767789	CD5 antigen-like [MGI:1334419]
354	Il5ra	0.909102572568407	0.268445651035497	interleukin 5 receptor%2C alpha [MGI:96558]
355	Cd74	0.918500035775561	0.158876511577439	CD74 antigen (invariant polypeptide of major histocompatibility complex%2C class II antigen-associated) [MGI:96534]
356	Cxcl11	0.940810683059146	0.23535517564965	chemokine (C-X-C motif) ligand 11 [MGI:1860203]
357	Il6	0.960223709160506	0.776054687822267	interleukin 6 [MGI:96559]
358	Tnfrsf17	0.971245430086521	0.555356586893095	tumor necrosis factor receptor superfamily%2C member 17 [MGI:1343050]
359	Ifi203-ps	0.98578251218713	0.105448014095494	interferon activated gene 203%2C pseudogene [MGI:3840117]
360	Cd209c	NA	NA	CD209c antigen [MGI:2157945]
361	Cd300ld2	NA	NA	CD300 molecule like family member D2 [MGI:3649405]
362	Cd55b	NA	NA	CD55 molecule%2C decay accelerating factor for complement B [MGI:104849]
363	Cd70	NA	NA	CD70 antigen [MGI:1195273]
364	Cd9-ps	NA	NA	Cd9 antigen%2C pseudogene [MGI:3645134]
365	Ccl1	NA	NA	chemokine (C-C motif) ligand 1 [MGI:98258]
366	Ccl19-ps1	NA	NA	chemokine (C-C motif) ligand 19%2C pseudogene 1 [MGI:1891387]
367	Ccl20	NA	NA	chemokine (C-C motif) ligand 20 [MGI:1329031]
368	Ccl21b	NA	NA	chemokine (C-C motif) ligand 21B (leucine) [MGI:1349182]
369	Ccl21d	NA	NA	chemokine (C-C motif) ligand 21D [MGI:5434896]
370	Ccl26	NA	NA	chemokine (C-C motif) ligand 26 [MGI:3589281]
371	Ccl27b	NA	NA	chemokine (C-C motif) ligand 27b [MGI:1891389]
372	Cxcl15	NA	NA	chemokine (C-X-C motif) ligand 15 [MGI:1339941]
373	Ifna1	NA	NA	interferon alpha 1 [MGI:107668]
374	Ifna11	NA	NA	interferon alpha 11 [MGI:109210]
375	Ifna12	NA	NA	interferon alpha 12 [MGI:2676324]
376	Ifna13	NA	NA	interferon alpha 13 [MGI:2667155]
377	Ifna14	NA	NA	interferon alpha 14 [MGI:3641425]
378	Ifna15	NA	NA	interferon alpha 15 [MGI:3649418]
379	Ifna16	NA	NA	interferon alpha 16 [MGI:3649260]
380	Ifna2	NA	NA	interferon alpha 2 [MGI:107666]

No	GeneSymbol	log2FoldChange	pvalue	Description
381	Ifna4	NA	NA	interferon alpha 4 [MGI:107664]
382	Ifna5	NA	NA	interferon alpha 5 [MGI:107663]
383	Ifna6	NA	NA	interferon alpha 6 [MGI:107662]
384	Ifna7	NA	NA	interferon alpha 7 [MGI:107661]
385	Ifna9	NA	NA	interferon alpha 9 [MGI:107659]
386	Ifnab	NA	NA	interferon alpha B [MGI:1097683]
387	Ifna-ps1	NA	NA	interferon alpha gene%2C pseudogene 1 [MGI:107669]
388	Ifnb1	NA	NA	interferon beta 1%2C fibroblast [MGI:107657]
389	Ifne	NA	NA	interferon epsilon [MGI:2667156]
390	Ifnl2	NA	NA	interferon lambda 2 [MGI:3647279]
391	Ifnl3	NA	NA	interferon lambda 3 [MGI:2450574]
392	Ifnz	NA	NA	interferon zeta [MGI:2448469]
393	Il1bos	NA	NA	interleukin 1 beta%2C opposite strand [MGI:3650458]
394	Il1f10	NA	NA	interleukin 1 family%2C member 10 [MGI:2652548]
395	Il1rapl2	NA	NA	interleukin 1 receptor accessory protein-like 2 [MGI:1913106]
396	Il17a	NA	NA	interleukin 17A [MGI:107364]
397	Il19	NA	NA	interleukin 19 [MGI:1890472]
398	Il20	NA	NA	interleukin 20 [MGI:1890473]
399	Il20ra	NA	NA	interleukin 20 receptor%2C alpha [MGI:3605069]
400	Il22	NA	NA	interleukin 22 [MGI:1355307]
401	Il22b	NA	NA	interleukin 22B [MGI:2151139]
402	Il23a	NA	NA	interleukin 23%2C alpha subunit p19 [MGI:1932410]
403	Il24	NA	NA	interleukin 24 [MGI:2135548]
404	Il25	NA	NA	interleukin 25 [MGI:2155888]
405	Il3	NA	NA	interleukin 3 [MGI:96552]
406	Il31	NA	NA	interleukin 31 [MGI:1923649]
407	Il36a	NA	NA	interleukin 36A [MGI:1859324]
408	Il36b	NA	NA	interleukin 36B [MGI:1916927]
409	Il9	NA	NA	interleukin 9 [MGI:96563]
410	Tgif1-ps	NA	NA	TGFB-induced factor homeobox 1%2C pseudogene [MGI:3779880]
411	Tgif2	NA	NA	TGFB-induced factor homeobox 2 [MGI:1915299]
412	Tgif2lx1	NA	NA	TGFB-induced factor homeobox 2-like%2C X-linked 1 [MGI:2387796]
413	Tgif2lx2	NA	NA	TGFB-induced factor homeobox 2-like%2C X-linked 2 [MGI:3800824]
414	Tgif2-ps1	NA	NA	TGFB-induced factor homeobox 2%2C pseudogene 1 [MGI:3649223]
415	Tnfsf18	NA	NA	tumor necrosis factor (ligand) superfamily%2C member 18 [MGI:2673064]
