# Supplementary material for: Identification of immune-associated genes with altered expression in the spleen of mice enriched with probiotic Lactobacillus species using RNA-seq profiling
Source: Anim Biosci. 2024 Aug 26;38(2):336–49. doi: 10.5713/ab.24.0280 (PMC11725755; doi:10.5713/ab.24.0280)
Supplement: Supplementary file 5 [file ab-24-0280-Supplementary-Table-4.pdf]

Supplementary Table S4A

Alig	Sublist	Cat	Term	Fold	P-Value	Count
1	1	BP	cell cycle	5.5	9.4E-46	101
2	2	BP	cell division	5.9	1.1E-31	67
3	3	BP	DNA replication	8.7	2.1E-18	29
4	4	BP	chromosome segregation	8.7	3.9E-17	27
5	5	BP	chemokine-mediated signaling pathway	12.3	2.9E-14	18
6	6	BP	immune response	3.6	6.0E-14	48
7	7	BP	DNA replication initiation	18.5	8.0E-13	13
8	8	BP	inflammatory response	3.7	1.1E-12	42
9	9	BP	mitotic spindle organization	11.2	5.5E-12	16
10	10	BP	mitotic sister chromatid segregation	15.4	1.2E-11	13
11	11	BP	mitotic cell cycle	6.6	1.4E-11	22
12	12	BP	porphyrin-containing compound biosynthetic process	25.4	1.8E-11	10
13	13	BP	chemotaxis	6.0	3.7E-11	23
14	14	BP	chromosome condensation	16.4	4.1E-11	12
15	15	BP	erythrocyte development	14.0	4.4E-11	13
16	16	BP	neutrophil chemotaxis	7.4	9.1E-10	17
17	17	BP	positive regulation of chromosome segregation	22.9	9.5E-10	9
18	18	BP	mitotic cytokinesis	8.7	1.1E-9	15
19	19	BP	cellular response to ionizing radiation	9.4	1.8E-9	14
20	20	BP	heme b biosynthetic process	31.1	1.3E-8	7
21	21	BP	positive regulation of chromosome condensation	31.1	1.3E-8	7
22	22	BP	mitotic spindle midzone assembly	27.7	3.7E-8	7
23	23	BP	heme O biosynthetic process	27.7	3.7E-8	7
24	24	BP	mitotic chromosome condensation	20.3	3.8E-8	8
25	25	BP	DNA unwinding involved in DNA replication	15.2	5.4E-8	9
26	26	BP	negative regulation of viral genome replication	8.0	5.9E-8	13
27	27	BP	heme biosynthetic process	14.5	8.3E-8	9
28	28	BP	heme a biosynthetic process	24.9	9.1E-8	7
29	29	BP	protoporphyrinogen IX biosynthetic process	24.9	9.1E-8	7
30	30	BP	positive regulation of chromosome separation	22.6	2.0E-7	7
31	31	BP	erythrocyte differentiation	6.5	6.0E-7	13
32	32	BP	DNA repair	2.8	8.9E-7	31
33	33	BP	cellular response to DNA damage stimulus	2.5	1.3E-6	37
34	34	BP	positive regulation of T cell proliferation	6.1	1.3E-6	13
35	35	BP	mitotic G2 DNA damage checkpoint	8.7	1.6E-6	10
36	36	BP	attachment of mitotic spindle microtubules to kinetochore	16.6	1.9E-6	7
37	37	BP	DNA duplex unwinding	9.7	2.7E-6	9
38	38	BP	regulation of cyclin-dependent protein serine/threonine kinase activity	9.7	2.7E-6	9
39	39	BP	spindle assembly involved in female meiosis I	35.6	3.0E-6	5
40	40	BP	kinetochore organization	35.6	3.0E-6	5
41	41	BP	calcium-mediated signaling	4.9	5.0E-6	14
42	42	BP	monocyte chemotaxis	8.7	6.9E-6	9
43	43	BP	microtubule-based movement	5.6	8.5E-6	12
44	44	BP	premeiotic DNA replication	29.6	8.8E-6	5
45	45	BP	meiotic chromosome segregation	29.6	8.8E-6	5
46	46	BP	double-strand break repair via break-induced replication	17.8	1.2E-5	6
47	47	BP	positive regulation of mitotic cell cycle spindle assembly checkpoint	17.8	1.2E-5	6
48	48	BP	lymphocyte chemotaxis	9.5	1.5E-5	8
49	49	BP	positive regulation of interferon-gamma production	4.8	1.6E-5	13
50	50	BP	cellular response to interleukin-1	5.3	1.6E-5	12
51	51	BP	meiotic chromosome condensation	25.4	2.0E-5	5
52	52	BP	cellular response to lipopolysaccharide	2.7	2.7E-5	24
53	53	BP	mitotic spindle assembly checkpoint	8.6	3.0E-5	8
54	54	BP	defense response to virus	3.0	3.1E-5	21
55	55	BP	negative regulation of cysteine-type endopeptidase activity	12.6	8.0E-5	6
56	56	BP	positive regulation of mitotic sister chromatid separation	35.6	8.6E-5	4
57	57	BP	acute-phase response	7.3	9.3E-5	8
58	58	BP	immune system process	2.1	9.7E-5	33

Alig	Sublist	Cat	Term	Fold	P-Value	Count
59	59	BP	positive regulation of mitotic cell cycle	7.1	1.1E-4	8
60	60	BP	G2/M transition of mitotic cell cycle	6.9	1.3E-4	8
61	61	BP	protein localization to CENP-A containing chromatin	11.2	1.4E-4	6
62	62	BP	replication fork processing	6.8	1.5E-4	8
63	63	BP	intrinsic apoptotic signaling pathway by p53 class mediator	6.8	1.5E-4	8
64	64	BP	mitotic metaphase plate congression	6.8	1.5E-4	8
65	65	BP	positive regulation of G2/M transition of mitotic cell cycle	8.3	1.6E-4	7
66	66	BP	monocyte differentiation	8.3	1.6E-4	7
67	67	BP	positive regulation of interleukin-1 beta production	5.0	1.6E-4	10
68	68	BP	cytokine-mediated signaling pathway	3.4	1.6E-4	15
69	69	BP	mitotic DNA replication checkpoint	16.2	1.7E-4	5
70	70	BP	eosinophil chemotaxis	10.7	1.9E-4	6
71	71	BP	nucleosome assembly	4.4	2.0E-4	11
72	72	BP	tetrapyrrole biosynthetic process	28.4	2.1E-4	4
73	73	BP	positive regulation of cytokinesis	6.3	2.4E-4	8
74	74	BP	kinetochore assembly	14.8	2.5E-4	5
75	75	BP	hemopoiesis	4.3	2.6E-4	11
76	76	BP	mitotic cell cycle phase transition	9.7	3.0E-4	6
77	77	BP	positive regulation of mitotic cytokinesis	23.7	4.1E-4	4
78	78	BP	positive regulation of cell cycle G2/M phase transition	23.7	4.1E-4	4
79	79	BP	metaphase plate congression	12.7	4.9E-4	5
80	80	BP	positive regulation of cell cycle	5.6	5.2E-4	8
81	81	BP	activation of cysteine-type endopeptidase activity	8.5	5.7E-4	6
82	82	BP	liver regeneration	6.6	6.1E-4	7
83	83	BP	cellular response to interferon-gamma	3.5	6.5E-4	12
84	84	BP	negative regulation of DNA binding	6.1	9.3E-4	7
85	85	BP	positive regulation of erythrocyte differentiation	7.6	9.9E-4	6
86	86	BP	chromosome organization	5.0	1.0E-3	8
87	87	BP	positive regulation of attachment of mitotic spindle microtubules to kinetochore	17.8	1.1E-3	4
88	88	BP	cell chemotaxis	3.8	1.2E-3	10
89	89	BP	DNA recombination	3.8	1.2E-3	10
90	90	BP	attachment of spindle microtubules to kinetochore	9.9	1.4E-3	5
91	91	BP	negative regulation of DNA recombination	9.9	1.4E-3	5
92	92	BP	double-strand break repair via homologous recombination	3.7	1.4E-3	10
93	93	BP	positive regulation of cytosolic calcium ion concentration	2.8	1.5E-3	14
94	94	BP	positive regulation of ERK1 and ERK2 cascade	2.5	1.6E-3	17
95	95	BP	hydrogen peroxide catabolic process	6.9	1.6E-3	6
96	96	BP	CENP-A containing nucleosome assembly	15.8	1.6E-3	4
97	97	BP	spindle organization	9.4	1.7E-3	5
98	98	BP	multicellular organismal iron ion homeostasis	6.7	1.8E-3	6
99	99	BP	response to interferon-gamma	6.5	2.1E-3	6
100	100	BP	response to platinum ion	35.6	2.3E-3	3
101	101	BP	heterochromatin organization	5.1	2.4E-3	7
102	102	BP	G1/S transition of mitotic cell cycle	4.3	2.5E-3	8
103	103	BP	negative regulation of gene expression, epigenetic	5.0	2.7E-3	7
104	104	BP	regulation of cell cycle	2.4	2.7E-3	16
105	105	BP	antimicrobial humoral immune response mediated by antimicrobial peptide	2.9	2.9E-3	12
106	106	BP	response to stilbenoid	8.1	3.0E-3	5
107	107	BP	positive regulation of ubiquitin protein ligase activity	12.9	3.1E-3	4
108	108	BP	positive regulation of interleukin-4 production	4.8	3.2E-3	7
109	109	BP	regulation of mitotic cell cycle	4.8	3.2E-3	7
110	110	BP	innate immune response	1.8	3.4E-3	27
111	111	BP	embryonic hemopoiesis	7.7	3.6E-3	5
112	112	BP	negative regulation of innate immune response	4.7	3.6E-3	7
113	113	BP	activation of innate immune response	4.6	3.9E-3	7
114	114	BP	protein localization to kinetochore	11.9	4.0E-3	4
115	115	BP	negative regulation of cysteine-type endopeptidase activity involved in apoptotic process	4.5	4.3E-3	7
116	116	BP	regulation of cytokinesis	5.5	4.5E-3	6
117	117	BP	protoporphyrinogen IX metabolic process	26.7	4.5E-3	3
118	118	BP	double-strand break repair	3.8	4.7E-3	8
119	119	BP	cellular oxidant detoxification	7.1	4.9E-3	5
120	120	BP	T cell costimulation	7.1	4.9E-3	5

Alig	Sublist	Cat	Term	Fold	P-Value	Count
121	121	BP	nucleotide biosynthetic process	10.9	5.1E-3	4
122	122	BP	interferon-gamma production	5.2	5.6E-3	6
123	123	BP	activation of protein kinase activity	6.6	6.5E-3	5
124	124	BP	negative regulation of interferon-gamma production	5.0	6.8E-3	6
125	125	BP	DNA-dependent DNA replication	6.4	7.4E-3	5
126	126	BP	positive regulation of hemoglobin biosynthetic process	21.3	7.4E-3	3
127	127	BP	positive regulation of natural killer cell chemotaxis	21.3	7.4E-3	3
128	128	BP	regulation of chemokine production	21.3	7.4E-3	3
129	129	BP	centrosome cycle	4.8	7.5E-3	6
130	130	BP	interspecies interaction between organisms	9.5	7.8E-3	4
131	131	BP	positive regulation of cytokine production	3.4	9.3E-3	8
132	132	BP	response to copper ion	8.9	9.4E-3	4
133	133	BP	regulation of DNA damage checkpoint	8.9	9.4E-3	4
134	134	BP	cellular response to xenobiotic stimulus	3.3	9.9E-3	8
135	135	BP	response to gamma radiation	5.7	1.1E-2	5
136	136	BP	homologous chromosome segregation	17.8	1.1E-2	3
137	137	BP	DNA replication-dependent nucleosome assembly	17.8	1.1E-2	3
138	138	BP	cytokinesis	17.8	1.1E-2	3
139	139	BP	regulation of DNA-dependent DNA replication initiation	17.8	1.1E-2	3
140	140	BP	centrosome duplication	8.4	1.1E-2	4
141	141	BP	negative regulation of extrinsic apoptotic signaling pathway in absence of ligand	5.6	1.2E-2	5
142	142	BP	negative regulation of myoblast differentiation	5.6	1.2E-2	5
143	143	BP	cellular response to interferon-beta	3.6	1.3E-2	7
144	144	BP	regulation of double-strand break repair via homologous recombination	7.9	1.3E-2	4
145	145	BP	defense response	2.7	1.3E-2	10
146	146	BP	positive regulation of interleukin-6 production	2.8	1.4E-2	9
147	147	BP	response to lipopolysaccharide	2.3	1.5E-2	12
148	148	BP	negative regulation of cytokinesis	15.2	1.5E-2	3
149	149	BP	positive regulation of fever generation	15.2	1.5E-2	3
150	150	BP	positive regulation of cell-cell adhesion mediated by integrin	15.2	1.5E-2	3
151	151	BP	immunoglobulin mediated immune response	2.2	1.5E-2	13
152	152	BP	positive regulation of T cell cytokine production	7.5	1.5E-2	4
153	153	BP	negative regulation of cell proliferation	1.8	1.6E-2	20
154	154	BP	base-excision repair	5.1	1.6E-2	5
155	155	BP	positive regulation of release of sequestered calcium ion into cytosol	5.1	1.6E-2	5
156	156	BP	T cell receptor signaling pathway	2.7	1.7E-2	9
157	157	BP	actin filament capping	7.1	1.8E-2	4
158	158	BP	regulation of mitotic spindle organization	7.1	1.8E-2	4
159	159	BP	protein polyubiquitination	2.4	1.8E-2	11
160	160	BP	platelet aggregation	4.9	1.8E-2	5
161	161	BP	positive regulation of DNA replication	4.8	2.0E-2	5
162	162	BP	positive regulation of osteoclast differentiation	4.8	2.0E-2	5
163	163	BP	positive regulation of alpha-beta T cell proliferation	13.3	2.0E-2	3
164	164	BP	porphyrin-containing compound metabolic process	13.3	2.0E-2	3
165	165	BP	positive regulation of G0 to G1 transition	13.3	2.0E-2	3
166	166	BP	TRIF-dependent toll-like receptor signaling pathway	13.3	2.0E-2	3
167	167	BP	positive regulation of spindle assembly	13.3	2.0E-2	3
168	168	BP	positive regulation of immunoglobulin production	3.8	2.0E-2	6
169	169	BP	macromolecular complex assembly	2.6	2.1E-2	9
170	170	BP	cellular response to glucose starvation	3.2	2.2E-2	7
171	171	BP	response to virus	2.8	2.3E-2	8
172	172	BP	positive regulation of exit from mitosis	11.9	2.5E-2	3
173	173	BP	DNA replication checkpoint	11.9	2.5E-2	3
174	174	BP	cellular response to tumor necrosis factor	2.4	2.5E-2	10
175	175	BP	positive regulation of T cell activation	4.4	2.5E-2	5
176	176	BP	positive regulation of T cell migration	6.2	2.6E-2	4
177	177	BP	response to cytokine	3.1	2.6E-2	7
178	178	BP	positive regulation of interleukin-2 production	4.3	2.7E-2	5
179	179	BP	adaptive immune response	1.8	2.8E-2	16
180	180	BP	killing of cells of other organism	3.0	2.9E-2	7
181	181	BP	chromosome organization involved in meiotic cell cycle	10.7	3.0E-2	3
182	182	BP	positive regulation of memory T cell activation	10.7	3.0E-2	3
183	183	BP	response to molecule of bacterial origin	10.7	3.0E-2	3

Alig	Sublist	Cat	Term	Fold	P-Value	Count
184	184	BP	response to bacterium	1.9	3.1E-2	15
185	185	BP	phospholipid biosynthetic process	3.4	3.2E-2	6
186	186	BP	regulation of DNA replication	4.0	3.4E-2	5
187	187	BP	mitotic spindle assembly	4.0	3.4E-2	5
188	188	BP	protein ubiquitination	1.6	3.5E-2	21
189	189	BP	positive regulation of interleukin-17 production	5.5	3.5E-2	4
190	190	BP	positive regulation of mitotic metaphase/anaphase transition	9.7	3.7E-2	3
191	191	BP	megakaryocyte differentiation	9.7	3.7E-2	3
192	192	BP	positive regulation of interleukin-10 production	4.0	3.7E-2	5
193	193	BP	DNA biosynthetic process	5.3	3.9E-2	4
194	194	BP	DNA damage checkpoint	3.9	4.0E-2	5
195	195	BP	positive regulation of G1/S transition of mitotic cell cycle	3.8	4.2E-2	5
196	196	BP	negative regulation of transcription, DNA-templated	1.5	4.3E-2	28
197	197	BP	positive regulation of monocyte chemotaxis	5.1	4.3E-2	4
198	198	BP	positive regulation of CD4-positive, alpha-beta T cell proliferation	8.9	4.3E-2	3
199	199	BP	positive regulation of glial cell proliferation	4.9	4.7E-2	4
200	200	BP	negative regulation of extrinsic apoptotic signaling pathway via death domain receptors	4.9	4.7E-2	4
201	201	BP	positive regulation of wound healing	4.9	4.7E-2	4
202	202	BP	positive regulation of GTPase activity	2.1	4.8E-2	10
203	203	BP	positive regulation of B cell proliferation	3.6	4.8E-2	5
204	1	CC	chromosome	3.8	8.2E-19	61
205	2	CC	chromosome, centromeric region	7.2	3.3E-17	31
206	3	CC	external side of plasma membrane	3.4	8.9E-14	51
207	4	CC	kinetochore	5.8	3.2E-12	26
208	5	CC	mitotic spindle	6.1	8.5E-11	22
209	6	CC	condensed chromosome outer kinetochore	23.9	6.7E-10	9
210	7	CC	nucleus	1.4	1.6E-9	241
211	8	CC	condensin complex	32.5	9.8E-9	7
212	9	CC	nucleoplasm	1.5	1.7E-8	149
213	10	CC	condensed chromosome, centromeric region	15.2	5.9E-8	9
214	11	CC	condensed chromosome	10.2	7.4E-8	11
215	12	CC	spindle pole	5.1	9.5E-8	18
216	13	CC	centrosome	2.5	1.0E-7	43
217	14	CC	cytoskeleton	1.9	1.5E-7	73
218	15	CC	CENP-A containing nucleosome	12.0	5.0E-7	9
219	16	CC	microtubule	3.0	7.2E-7	29
220	17	CC	cytoplasm	1.3	8.4E-7	253
221	18	CC	nucleosome	4.3	8.9E-7	18
222	19	CC	extracellular space	1.7	8.9E-7	89
223	20	CC	condensed nuclear chromosome	7.4	1.8E-6	11
224	21	CC	nuclear chromosome	11.9	3.1E-6	8
225	22	CC	spindle	4.3	5.1E-6	16
226	23	CC	CMG complex	20.3	5.5E-6	6
227	24	CC	chromatin	2.5	6.6E-6	32
228	25	CC	spindle midzone	10.6	7.0E-6	8
229	26	CC	microtubule cytoskeleton	3.4	7.3E-6	20
230	27	CC	chromosome passenger complex	31.0	7.4E-6	5
231	28	CC	spindle microtubule	8.6	7.6E-6	9
232	29	CC	midbody	3.4	7.8E-6	20
233	30	CC	cortical cytoskeleton	9.0	2.3E-5	8
234	31	CC	cytosol	1.3	3.3E-5	156
235	32	CC	mitotic spindle midzone	14.9	3.3E-5	6
236	33	CC	kinesin complex	7.0	3.8E-5	9
237	34	CC	intercellular bridge	4.3	1.0E-4	12
238	35	CC	MCM complex	16.9	1.5E-4	5
239	36	CC	replication fork	8.4	1.5E-4	7
240	37	CC	cell surface	1.9	2.6E-4	40
241	38	CC	hemoglobin complex	14.3	3.0E-4	5
242	39	CC	cyclin-dependent protein kinase holoenzyme complex	7.2	3.6E-4	7
243	40	CC	spectrin-associated cytoskeleton	24.8	3.6E-4	4
244	41	CC	haptoglobin-hemoglobin complex	13.3	4.2E-4	5
245	42	CC	chromocenter	12.4	5.5E-4	5
246	43	CC	nuclear ubiquitin ligase complex	21.2	6.2E-4	4

Ali g	Sublis t	Ca t	Term	Fol d	P-Value	Coun t
247	44	CC	mitotic spindle pole	6.3	7.4E-4	7
248	45	CC	extracellular region	1.5	9.6E-4	74
249	46	CC	condensed chromosome inner kinetochore	9.8	1.4E-3	5
250	47	CC	lateral element	9.3	1.8E-3	5
251	48	CC	macromolecular complex	1.6	3.5E-3	41
252	49	CC	Rb-E2F complex	22.3	6.8E-3	3
253	50	CC	pronucleus	9.9	6.9E-3	4
254	51	CC	high-density lipoprotein particle	6.2	8.1E-3	5
255	52	CC	chromosome, telomeric region	2.9	8.5E-3	10
256	53	CC	male germ cell nucleus	3.4	9.5E-3	8
257	54	CC	spindle pole centrosome	7.4	1.6E-2	4
258	55	CC	intracellular membrane-bounded organelle	1.4	1.8E-2	42
259	56	CC	nuclear origin of replication recognition complex	13.9	1.8E-2	3
260	57	CC	XY body	6.2	2.6E-2	4
261	58	CC	RNA polymerase II transcription factor complex	2.5	2.7E-2	9
262	59	CC	centriole	2.3	3.3E-2	10
263	60	CC	transcription factor complex	1.9	3.4E-2	15
264	61	CC	protein phosphatase type 1 complex	8.6	4.6E-2	3
265	1	MF	microtubule binding	4.1	6.4E-11	32
266	2	MF	chemokine activity	10.1	1.2E-10	15
267	3	MF	cytokine activity	4.2	3.4E-10	29
268	4	MF	structural constituent of chromatin	5.7	4.8E-9	19
269	5	MF	microtubule motor activity	7.9	2.3E-7	12
270	6	MF	single-stranded DNA-dependent ATP-dependent DNA helicase activity	13.1	1.4E-6	8
271	7	MF	nucleotide binding	1.7	1.6E-6	85
272	8	MF	ATPase activity	2.7	1.6E-6	32
273	9	MF	anaphase-promoting complex binding	20.7	4.5E-6	6
274	10	MF	nucleosomal DNA binding	6.4	6.8E-6	11
275	11	MF	single-stranded DNA binding	4.4	1.8E-5	14
276	12	MF	ATP binding	1.7	1.9E-5	74
277	13	MF	CXCR chemokine receptor binding	15.9	2.1E-5	6
278	14	MF	CCR chemokine receptor binding	8.9	2.4E-5	8
279	15	MF	double-stranded DNA binding	3.4	4.6E-5	17
280	16	MF	DNA replication origin binding	13.8	4.8E-5	6
281	17	MF	C-C chemokine binding	10.0	5.0E-5	7
282	18	MF	DNA binding	1.5	5.1E-5	84
283	19	MF	identical protein binding	1.5	1.1E-4	88
284	20	MF	heme binding	3.0	1.6E-4	17
285	21	MF	peroxidase activity	6.6	1.9E-4	8
286	22	MF	cyclin-dependent protein serine/threonine kinase regulator activity	7.3	3.3E-4	7
287	23	MF	haptoglobin binding	13.2	4.1E-4	5
288	24	MF	cytokine receptor activity	5.6	5.0E-4	8
289	25	MF	C-C chemokine receptor activity	8.3	6.6E-4	6
290	26	MF	ATP-dependent microtubule motor activity, plus-end-directed	11.5	7.4E-4	5
291	27	MF	oxygen binding	7.9	8.0E-4	6
292	28	MF	oxygen transporter activity	10.8	9.6E-4	5
293	29	MF	core promoter proximal region sequence-specific DNA binding	3.7	1.5E-3	10
294	30	MF	coreceptor activity	5.2	2.0E-3	7
295	31	MF	protein kinase binding	1.8	2.8E-3	30
296	32	MF	chemokine receptor activity	8.2	2.8E-3	5
297	33	MF	DNA helicase activity	5.7	3.6E-3	6
298	34	MF	histone binding	2.6	4.3E-3	13
299	35	MF	protein homodimerization activity	1.6	4.8E-3	39
300	36	MF	protein heterodimerization activity	1.9	4.9E-3	22
301	37	MF	organic acid binding	10.6	5.6E-3	4
302	38	MF	transcription factor binding	2.8	5.7E-3	11
303	39	MF	3'-5' DNA helicase activity	9.8	7.0E-3	4
304	40	MF	chromatin binding	1.7	7.0E-3	28
305	41	MF	CXCR3 chemokine receptor binding	20.7	7.9E-3	3
306	42	MF	enzyme binding	1.8	8.9E-3	24
307	43	MF	interleukin-1 receptor binding	8.6	1.0E-2	4
308	44	MF	kinetochore binding	17.2	1.2E-2	3
309	45	MF	tumor necrosis factor receptor binding	5.2	1.5E-2	5

Alig	Sublist	Cat	Term	Fold	P-Value	Count
310	46	MF	protein binding	1.2	1.5E-2	183
311	47	MF	helicase activity	2.6	1.6E-2	10
312	48	MF	bubble DNA binding	14.8	1.6E-2	3
313	49	MF	tumor necrosis factor receptor superfamily binding	14.8	1.6E-2	3
314	50	MF	CCR1 chemokine receptor binding	14.8	1.6E-2	3
315	51	MF	magnesium ion binding	2.0	2.3E-2	14
316	52	MF	glycine transmembrane transporter activity	11.5	2.6E-2	3
317	53	MF	DNA polymerase binding	5.7	3.1E-2	4
318	54	MF	hemoglobin binding	10.3	3.2E-2	3
319	55	MF	receptor agonist activity	2.8	3.7E-2	7
320	56	MF	monooxygenase activity	2.5	3.8E-2	8
321	57	MF	DNA-(apurinic or apyrimidinic site) lyase activity	9.4	3.9E-2	3
322	58	MF	transferase activity	1.3	4.4E-2	65
323	59	MF	lyase activity	2.1	4.5E-2	10
324	60	MF	kinase activity	1.5	4.7E-2	28
325	61	MF	iron-sulfur cluster binding	3.0	5.0E-2	6
326	62	MF	damaged DNA binding	3.0	5.0E-2	6

Supplementary Table S4B

Align	Sublist	Category	Term	Fold	P-Value	Count
1	1	BP	immune response	8.0	1.6E-18	31
2	2	BP	inflammatory response	7.3	2.2E-13	24
3	3	BP	defense response to virus	9.7	2.3E-13	20
4	4	BP	chemokine-mediated signaling pathway	28.3	3.3E-13	12
5	5	BP	chemotaxis	14.3	3.8E-13	16
6	6	BP	positive regulation of interleukin-1 beta production	22.4	4.6E-13	13
7	7	BP	neutrophil chemotaxis	17.9	6.1E-11	12
8	8	BP	negative regulation of viral genome replication	21.1	9.3E-10	10
9	9	BP	negative regulation of cysteine-type endopeptidase activity	50.4	3.0E-9	7
10	10	BP	monocyte differentiation	32.6	3.6E-9	8
11	11	BP	immune system process	4.9	4.5E-9	22
12	12	BP	cellular response to ionizing radiation	20.8	1.0E-8	9
13	13	BP	positive regulation of T cell proliferation	16.1	1.1E-8	10
14	14	BP	activation of cysteine-type endopeptidase activity	34.3	4.1E-8	7
15	15	BP	intrinsic apoptotic signaling pathway by p53 class mediator	23.3	4.3E-8	8
16	16	BP	negative regulation of gene expression, epigenetic	19.6	1.5E-7	8
17	17	BP	positive regulation of cytosolic calcium ion concentration	8.3	2.2E-7	12
18	18	BP	negative regulation of innate immune response	18.5	2.3E-7	8
19	19	BP	activation of innate immune response	18.1	2.6E-7	8
20	20	BP	cellular response to lipopolysaccharide	5.9	2.7E-7	15
21	21	BP	positive regulation of tyrosine phosphorylation of STAT protein	17.2	3.8E-7	8
22	22	BP	positive regulation of inflammatory response	12.4	6.4E-7	9
23	23	BP	negative regulation of DNA binding	20.9	9.3E-7	7
24	24	BP	cellular response to interferon-beta	14.2	1.4E-6	8
25	25	BP	response to virus	11.0	1.6E-6	9
26	26	BP	erythrocyte development	22.3	6.6E-6	6
27	27	BP	monocyte chemotaxis	19.9	1.2E-5	6
28	28	BP	positive regulation of osteoclast differentiation	19.9	1.2E-5	6
29	29	BP	eosinophil chemotaxis	30.6	1.8E-5	5
30	30	BP	innate immune response	3.8	2.3E-5	16
31	31	BP	antimicrobial humoral immune response mediated by antimicrobial peptide	7.5	2.7E-5	9
32	32	BP	heme biosynthetic process	27.8	2.7E-5	5
33	33	BP	cellular response to glucose starvation	11.0	4.1E-5	7
34	34	BP	cytokine-mediated signaling pathway	6.9	4.8E-5	9
35	35	BP	killing of cells of other organism	10.3	5.9E-5	7
36	36	BP	positive regulation of cytokine production	10.2	6.3E-5	7
37	37	BP	positive regulation of monocyte chemotaxis	21.9	7.4E-5	5
38	38	BP	lymphocyte chemotaxis	20.4	9.7E-5	5

Align	Sublist	Category	Term	Fold	P-Value	Count
39	39	BP	hydrogen peroxide catabolic process	19.7	1.1E-4	5
40	40	BP	microglial cell activation	19.1	1.3E-4	5
41	41	BP	calcium-mediated signaling	8.5	1.8E-4	7
42	42	BP	positive regulation of calcidiol 1-monooxygenase activity	122.4	2.0E-4	3
43	43	BP	positive regulation of interleukin-6 production	7.6	3.2E-4	7
44	44	BP	positive regulation of interleukin-10 production	13.6	4.8E-4	5
45	45	BP	cellular response to interferon-gamma	7.0	4.9E-4	7
46	46	BP	defense response	6.4	8.0E-4	7
47	47	BP	positive regulation of ERK1 and ERK2 cascade	4.5	9.0E-4	9
48	48	BP	positive regulation of NF-kappaB transcription factor activity	6.2	9.7E-4	7
49	49	BP	positive regulation of chemokine production	11.3	9.7E-4	5
50	50	BP	cellular oxidant detoxification	19.6	1.1E-3	4
51	51	BP	positive regulation of macrophage chemotaxis	19.6	1.1E-3	4
52	52	BP	positive regulation of gene expression	2.9	1.2E-3	14
53	53	BP	positive regulation of peptidyl-serine phosphorylation	7.5	1.2E-3	6
54	54	BP	negative regulation of transcription, DNA-templated	2.7	1.3E-3	15
55	55	BP	positive regulation of fever generation	52.5	1.3E-3	3
56	56	BP	positive regulation of MAP kinase activity	9.6	1.8E-3	5
57	57	BP	positive regulation of cytokine production involved in inflammatory response	15.8	2.0E-3	4
58	58	BP	macrophage differentiation	15.8	2.0E-3	4
59	59	BP	positive regulation of GTPase activity	5.1	2.5E-3	7
60	60	BP	positive regulation of cell adhesion	8.5	2.8E-3	5
61	61	BP	positive regulation of release of sequestered calcium ion into cytosol	14.0	2.8E-3	4
62	62	BP	positive regulation of transcription from RNA polymerase II promoter	2.0	3.1E-3	21
63	63	BP	cytoskeleton organization	6.0	3.3E-3	6
64	64	BP	T cell chemotaxis	33.4	3.4E-3	3
65	65	BP	response to lipopolysaccharide	4.7	3.8E-3	7
66	66	BP	cellular response to interleukin-1	7.6	4.3E-3	5
67	67	BP	regulation of insulin secretion	11.9	4.5E-3	4
68	68	BP	positive regulation of cell migration	3.9	4.6E-3	8
69	69	BP	negative regulation of interferon-gamma production	11.4	5.1E-3	4
70	70	BP	cellular response to interleukin-17	26.2	5.6E-3	3
71	71	BP	interspecies interaction between organisms	24.5	6.4E-3	3
72	72	BP	cell chemotaxis	6.6	7.0E-3	5
73	73	BP	positive regulation of membrane protein ectodomain proteolysis	23.0	7.3E-3	3
74	74	BP	cellular response to tumor necrosis factor	4.9	7.4E-3	6
75	75	BP	positive regulation of interferon-gamma production	6.4	7.8E-3	5
76	76	BP	tumor necrosis factor-mediated signaling pathway	9.6	8.2E-3	4
77	77	BP	osteoclast differentiation	9.4	8.7E-3	4
78	78	BP	response to bacterium	3.4	8.8E-3	8
79	79	BP	positive regulation of nitric oxide biosynthetic process	9.2	9.1E-3	4
80	80	BP	negative regulation of interleukin-6 production	9.1	9.6E-3	4
81	81	BP	negative regulation of cysteine-type endopeptidase activity involved in apoptotic process	8.9	1.0E-2	4
82	82	BP	extrinsic apoptotic signaling pathway	8.7	1.1E-2	4
83	83	BP	negative regulation of transcription from RNA polymerase II promoter	2.0	1.1E-2	17
84	84	BP	positive regulation of myoblast fusion	18.4	1.1E-2	3
85	85	BP	positive regulation of interleukin-8 production	8.2	1.3E-2	4
86	86	BP	negative regulation of cell proliferation	2.8	1.5E-2	9
87	87	BP	positive regulation of sequence-specific DNA binding transcription factor activity	5.2	1.6E-2	5
88	88	BP	positive regulation of vitamin D biosynthetic process	122.4	1.6E-2	2
89	89	BP	positive regulation of fever generation by positive regulation of prostaglandin secretion	122.4	1.6E-2	2
90	90	BP	positive regulation of ERK1 and ERK2 cascade via TNFSF11-mediated signaling	122.4	1.6E-2	2
91	91	BP	positive regulation of I-kappaB kinase/NF-kappaB signaling	4.0	1.7E-2	6
92	92	BP	T cell proliferation	7.2	1.8E-2	4
93	93	BP	erythrocyte differentiation	6.9	2.0E-2	4
94	94	BP	positive regulation of activated T cell proliferation	13.6	2.0E-2	3
95	95	BP	I-kappaB kinase/NF-kappaB signaling	6.7	2.2E-2	4
96	96	BP	positive regulation of vascular endothelial growth factor production	12.7	2.3E-2	3

Align	Sublist	Category	Term	Fold	P-Value	Count
97	97	BP	positive regulation of bone resorption	12.7	2.3E-2	3
98	98	BP	negative regulation of thrombin-activated receptor signaling pathway	81.6	2.4E-2	2
99	99	BP	inflammatory response to antigenic stimulus	12.2	2.5E-2	3
100	100	BP	cell-cell signaling	6.1	2.7E-2	4
101	101	BP	negative regulation of extrinsic apoptotic signaling pathway in absence of ligand	11.5	2.8E-2	3
102	102	BP	response to interleukin-1	11.1	2.9E-2	3
103	103	BP	positive regulation of protein serine/threonine kinase activity	11.1	2.9E-2	3
104	104	BP	response to interferon-gamma	11.1	2.9E-2	3
105	105	BP	positive regulation of cell proliferation	2.2	3.1E-2	11
106	106	BP	sequestering of triglyceride	61.2	3.2E-2	2
107	107	BP	interleukin-6 production	10.2	3.4E-2	3
108	108	BP	liver regeneration	9.7	3.8E-2	3
109	109	BP	acute-phase response	9.4	4.0E-2	3
110	110	BP	fever generation	49.0	4.0E-2	2
111	111	BP	positive regulation of natural killer cell chemotaxis	49.0	4.0E-2	2
112	112	BP	tetrapyrrole biosynthetic process	49.0	4.0E-2	2
113	113	BP	positive regulation of nitrogen compound metabolic process	49.0	4.0E-2	2
114	114	BP	positive regulation of hemoglobin biosynthetic process	49.0	4.0E-2	2
115	115	BP	interferon-gamma production	9.0	4.4E-2	3
116	116	BP	response to xenobiotic stimulus	2.7	4.4E-2	7
117	117	BP	positive regulation of neuron apoptotic process	5.0	4.5E-2	4
118	118	BP	negative regulation of osteoclast differentiation	8.7	4.6E-2	3
119	119	BP	TNFSF11-mediated signaling pathway	40.8	4.8E-2	2
120	120	BP	cytokinesis	40.8	4.8E-2	2
121	121	BP	positive regulation of smooth muscle cell apoptotic process	40.8	4.8E-2	2
122	122	BP	response to fructose	40.8	4.8E-2	2
123	123	BP	positive regulation of interleukin-12 production	8.3	5.0E-2	3
124	1	CC	external side of plasma membrane	6.3	4.8E-14	28
125	2	CC	extracellular space	2.8	1.5E-9	43
126	3	CC	extracellular region	2.6	6.5E-8	39
127	4	CC	cell surface	3.1	2.1E-5	20
128	5	CC	hemoglobin complex	38.5	1.3E-4	4
129	6	CC	haptoglobin-hemoglobin complex	35.8	1.7E-4	4
130	7	CC	cortical cytoskeleton	15.2	2.3E-3	4
131	8	CC	CENP-A containing nucleosome	13.4	2.1E-2	3
132	9	CC	cytosol	1.3	2.6E-2	46
133	10	CC	nucleosome	4.1	3.5E-2	5
134	11	CC	actin filament	4.9	4.9E-2	4
135	12	CC	mitochondrial matrix	3.0	5.1E-2	6
136	13	CC	endosome	1.9	7.3E-2	11
137	14	CC	cleavage furrow	6.5	7.8E-2	3
138	15	CC	cytoplasmic vesicle	1.7	8.4E-2	12
139	16	CC	basolateral plasma membrane	2.5	9.6E-2	6
140	1	MF	cytokine activity	12.1	5.6E-19	25
141	2	MF	chemokine activity	27.0	5.1E-13	12
142	3	MF	double-stranded DNA binding	7.3	2.7E-6	11
143	4	MF	CXCR chemokine receptor binding	44.2	3.6E-6	5
144	5	MF	cytokine receptor activity	14.1	6.4E-5	6
145	6	MF	identical protein binding	2.0	1.1E-4	35
146	7	MF	CCR chemokine receptor binding	18.5	1.4E-4	5
147	8	MF	transcription factor binding	6.9	1.7E-4	8
148	9	MF	organic acid binding	35.3	1.7E-4	4
149	10	MF	haptoglobin binding	35.3	1.7E-4	4
150	11	MF	cytokine binding	15.1	3.2E-4	5
151	12	MF	oxygen transporter activity	28.7	3.3E-4	4
152	13	MF	peroxidase activity	13.7	4.7E-4	5
153	14	MF	CXCR3 chemokine receptor binding	68.9	7.3E-4	3
154	15	MF	oxygen binding	17.7	1.4E-3	4
155	16	MF	transmembrane signaling receptor activity	4.3	2.5E-3	8
156	17	MF	tumor necrosis factor receptor binding	13.9	2.9E-3	4
157	18	MF	hemoglobin binding	34.5	3.2E-3	3
158	19	MF	interleukin-1 receptor binding	21.5	8.2E-3	3
159	20	MF	chemokine receptor activity	16.4	1.4E-2	3

Align	Sublist	Category	Term	Fold	P-Value	Count
160	21	MF	Hsp70 protein binding	7.5	1.6E-2	4
161	22	MF	structural constituent of chromatin	5.0	1.7E-2	5
162	23	MF	C-C chemokine binding	14.4	1.8E-2	3
163	24	MF	C-C chemokine receptor activity	13.8	2.0E-2	3
164	25	MF	heme binding	3.6	2.6E-2	6
165	26	MF	interleukin-20 binding	76.6	2.6E-2	2
166	27	MF	interleukin-22 receptor activity	76.6	2.6E-2	2
167	28	MF	chemokine (C-C motif) ligand 5 binding	57.4	3.4E-2	2
168	29	MF	hemoglobin beta binding	57.4	3.4E-2	2
169	30	MF	polyubiquitin binding	8.6	4.7E-2	3
170	31	MF	cytokine receptor binding	8.4	4.9E-2	3

Supplementary Table S4C

Align	Sublist	Category	Term	Fold Enrichment	P-Value	Count
1	1	BP	immune response	13.8	3.6E-20	24
2	2	BP	defense response to virus	18.4	5.7E-16	17
3	3	BP	inflammatory response	11.5	8.8E-13	17
4	4	BP	innate immune response	8.4	4.4E-10	16
5	5	BP	chemokine-mediated signaling pathway	42.0	7.0E-10	8
6	6	BP	chemotaxis	19.9	1.6E-9	10
7	7	BP	positive regulation of T cell proliferation	28.7	1.1E-8	8
8	8	BP	positive regulation of interferon-gamma production	22.7	5.5E-8	8
9	9	BP	cytokine-mediated signaling pathway	15.5	1.1E-7	9
10	10	BP	positive regulation of interleukin-12 production	37.2	5.2E-7	6
11	11	BP	immune system process	6.4	6.2E-7	13
12	12	BP	response to virus	19.1	1.6E-6	7
13	13	BP	cellular response to interferon-beta	23.7	5.0E-6	6
14	14	BP	positive regulation of interleukin-1 beta production	23.1	5.8E-6	6
15	15	BP	neutrophil chemotaxis	20.0	1.2E-5	6
16	16	BP	positive regulation of cytokine production	19.5	1.3E-5	6
17	17	BP	antimicrobial humoral immune response mediated by antimicrobial peptide	13.0	1.5E-5	7
18	18	BP	interspecies interaction between organisms	72.8	2.0E-5	4
19	19	BP	positive regulation of interleukin-10 production	30.3	2.1E-5	5
20	20	BP	response to bacterium	7.7	7.6E-5	8
21	21	BP	cellular response to diacyl bacterial lipopeptide	204.7	7.7E-5	3
22	22	BP	cellular response to interferon-gamma	13.4	8.0E-5	6
23	23	BP	positive regulation of activated T cell proliferation	40.4	1.2E-4	4
24	24	BP	cellular response to lipopolysaccharide	7.0	1.3E-4	8
25	25	BP	toll-like receptor signaling pathway	39.0	1.4E-4	4
26	26	BP	positive regulation of defense response to virus by host	33.1	2.3E-4	4
27	27	BP	positive regulation of ERK1 and ERK2 cascade	7.8	2.6E-4	7
28	28	BP	positive regulation of inflammatory response	15.3	3.0E-4	5
29	29	BP	interleukin-12-mediated signaling pathway	102.4	3.6E-4	3
30	30	BP	positive regulation of cytosolic calcium ion concentration	9.3	4.5E-4	6
31	31	BP	MyD88-dependent toll-like receptor signaling pathway	91.0	4.6E-4	3
32	32	BP	response to molecule of bacterial origin	81.9	5.7E-4	3
33	33	BP	regulation of adaptive immune response	81.9	5.7E-4	3
34	34	BP	positive regulation of T-helper 1 type immune response	74.4	6.9E-4	3
35	35	BP	positive regulation of interleukin-6 production	12.1	7.5E-4	5
36	36	BP	negative regulation of innate immune response	20.6	9.3E-4	4
37	37	BP	activation of innate immune response	20.2	9.8E-4	4
38	38	BP	positive regulation of immunoglobulin production	19.5	1.1E-3	4
39	39	BP	negative regulation of viral genome replication	18.8	1.2E-3	4
40	40	BP	negative regulation of inflammatory response	10.3	1.4E-3	5
41	41	BP	defense response	10.2	1.4E-3	5
42	42	BP	negative regulation of cysteine-type endopeptidase activity	48.2	1.7E-3	3
43	43	BP	negative regulation of interleukin-12 production	43.1	2.1E-3	3
44	44	BP	eosinophil chemotaxis	40.9	2.3E-3	3

Align	Sublist	Category	Term	Fold Enrichment	P-Value	Count
45	45	BP	positive regulation of myoblast fusion	40.9	2.3E-3	3
46	46	BP	negative regulation of interleukin-17 production	40.9	2.3E-3	3
47	47	BP	cellular response to interferon-alpha	39.0	2.6E-3	3
48	48	BP	negative regulation of interleukin-10 production	37.2	2.8E-3	3
49	49	BP	cellular response to interleukin-1	13.5	3.2E-3	4
50	50	BP	killing of cells of other organism	13.2	3.4E-3	4
51	51	BP	activation of cysteine-type endopeptidase activity	32.8	3.7E-3	3
52	52	BP	lymphocyte chemotaxis	27.3	5.2E-3	3
53	53	BP	monocyte differentiation	27.3	5.2E-3	3
54	54	BP	positive regulation of transcription from RNA polymerase II promoter	2.6	5.4E-3	12
55	55	BP	microglial cell activation	25.6	6.0E-3	3
56	56	BP	immunoglobulin mediated immune response	6.5	6.9E-3	5
57	57	BP	toll-like receptor TLR6:TLR2 signaling pathway	273.0	7.2E-3	2
58	58	BP	detection of diacyl bacterial lipopeptide	273.0	7.2E-3	2
59	59	BP	monocyte chemotaxis	22.1	7.9E-3	3
60	60	BP	negative regulation of DNA binding	20.0	9.6E-3	3
61	61	BP	interferon-gamma production	20.0	9.6E-3	3
62	62	BP	intrinsic apoptotic signaling pathway by p53 class mediator	19.5	1.0E-2	3
63	63	BP	nitric oxide metabolic process	182.0	1.1E-2	2
64	64	BP	cellular response to triacyl bacterial lipopeptide	182.0	1.1E-2	2
65	65	BP	response to bacterial lipoprotein	182.0	1.1E-2	2
66	66	BP	defense response to protozoan	18.2	1.2E-2	3
67	67	BP	positive regulation of cell migration	5.4	1.3E-2	5
68	68	BP	negative regulation of gene expression, epigenetic	16.4	1.4E-2	3
69	69	BP	defense response to nematode	136.5	1.4E-2	2
70	70	BP	positive regulation of interferon-beta production	15.7	1.5E-2	3
71	71	BP	positive regulation of interleukin-4 production	15.7	1.5E-2	3
72	72	BP	positive regulation of tumor necrosis factor production	7.5	1.6E-2	4
73	73	BP	cellular response to ionizing radiation	15.5	1.6E-2	3
74	74	BP	positive regulation of chemokine production	15.2	1.6E-2	3
75	75	BP	B cell adhesion	109.2	1.8E-2	2
76	76	BP	negative regulation of complement-dependent cytotoxicity	109.2	1.8E-2	2
77	77	BP	positive regulation of tyrosine phosphorylation of STAT protein	14.4	1.8E-2	3
78	78	BP	positive regulation of interleukin-8 production	13.6	2.0E-2	3
79	79	BP	response to glucocorticoid	13.2	2.1E-2	3
80	80	BP	positive regulation of NK T cell activation	91.0	2.1E-2	2
81	81	BP	regulation of tyrosine phosphorylation of STAT protein	78.0	2.5E-2	2
82	82	BP	interleukin-11-mediated signaling pathway	78.0	2.5E-2	2
83	83	BP	negative regulation of regulatory T cell differentiation	78.0	2.5E-2	2
84	84	BP	T cell proliferation	12.0	2.5E-2	3
85	85	BP	negative regulation of tumor necrosis factor production	11.7	2.7E-2	3
86	86	BP	cell surface pattern recognition receptor signaling pathway	68.2	2.9E-2	2
87	87	BP	B cell proliferation	10.6	3.2E-2	3
88	88	BP	germinal center B cell differentiation	60.7	3.2E-2	2
89	89	BP	cellular response to glucose starvation	10.5	3.2E-2	3
90	90	BP	positive regulation of viral life cycle	54.6	3.6E-2	2
91	91	BP	cellular response to lipoteichoic acid	54.6	3.6E-2	2
92	92	BP	peptidyl-tyrosine phosphorylation	9.6	3.8E-2	3
93	93	BP	negative regulation of heterotypic cell-cell adhesion	49.6	3.9E-2	2
94	94	BP	positive regulation of MHC class II biosynthetic process	45.5	4.3E-2	2
95	95	BP	cell chemotaxis	8.8	4.5E-2	3
96	96	BP	positive regulation of B cell activation	42.0	4.6E-2	2
97	97	BP	interleukin-10 production	42.0	4.6E-2	2
98	1	CC	external side of plasma membrane	12.5	5.5E-20	25
99	2	CC	extracellular region	4.1	1.0E-10	28
100	3	CC	extracellular space	3.8	1.2E-9	27
101	4	CC	cell surface	4.9	4.2E-6	14
102	5	CC	receptor complex	9.4	9.4E-5	7
103	6	CC	Toll-like receptor 2-Toll-like receptor 6 protein complex	278.4	7.1E-3	2
104	7	CC	interleukin-23 receptor complex	139.2	1.4E-2	2
105	8	CC	membrane raft	4.7	5.1E-2	4
106	1	MF	cytokine activity	17.0	1.8E-14	16
107	2	MF	chemokine activity	39.7	1.0E-9	8

Align	Sublist	Category	Term	Fold Enrichment	P-Value	Count
108	3	MF	cytokine receptor activity	36.1	3.5E-8	7
109	4	MF	NAD+ nucleotidase, cyclic ADP-ribose generating	56.2	4.4E-5	4
110	5	MF	NAD(P)+ nucleosidase activity	56.2	4.4E-5	4
111	6	MF	signaling receptor activity	9.6	8.2E-5	7
112	7	MF	lipopeptide binding	126.5	2.2E-4	3
113	8	MF	cytokine binding	26.6	4.3E-4	4
114	9	MF	cytokine receptor binding	24.7	5.4E-4	4
115	10	MF	CXCR chemokine receptor binding	58.4	1.1E-3	3
116	11	MF	transmembrane signaling receptor activity	7.2	1.4E-3	6
117	12	MF	CCR chemokine receptor binding	24.5	6.5E-3	3
118	13	MF	interleukin-23 receptor activity	253.0	7.8E-3	2
119	14	MF	diacyl lipopeptide binding	253.0	7.8E-3	2
120	15	MF	interleukin-23 binding	253.0	7.8E-3	2
121	16	MF	interleukin-12 receptor binding	126.5	1.6E-2	2
122	17	MF	growth factor activity	6.7	2.2E-2	4
123	18	MF	lipoteichoic acid binding	72.3	2.7E-2	2
124	19	MF	double-stranded DNA binding	5.9	3.0E-2	4

Supplementary Table S5A

Sublist	Term	Fold Enrichment	P-Value	Count
1	Cell cycle	8.6	1.5E-28	44
2	Cytokine-cytokine receptor interaction	5.2	1.4E-22	51
3	Viral protein interaction with cytokine and cytokine receptor	8.0	2.7E-15	25
4	Hematopoietic cell lineage	5.8	8.8E-9	18
5	Chemokine signaling pathway	3.9	1.7E-8	25
6	DNA replication	9.5	1.2E-7	11
7	Oocyte meiosis	4.0	9.7E-6	16
8	Systemic lupus erythematosus	3.4	3.1E-5	17
9	Porphyrin metabolism	6.3	6.8E-5	9
10	Malaria	5.3	9.2E-5	10
11	Progesterone-mediated oocyte maturation	3.9	2.1E-4	12
12	p53 signaling pathway	4.1	6.2E-4	10
13	Inflammatory bowel disease	4.4	9.2E-4	9
14	Cellular senescence	2.7	9.3E-4	16
15	Rheumatoid arthritis	3.5	2.2E-3	10
16	Human T-cell leukemia virus 1 infection	2.2	3.3E-3	18
17	Virion - Human immunodeficiency virus	12.1	3.6E-3	4
18	Neutrophil extracellular trap formation	2.3	3.6E-3	16
19	JAK-STAT signaling pathway	2.5	4.3E-3	14
20	Epstein-Barr virus infection	2.1	8.5E-3	16
21	Asthma	6.0	8.5E-3	5
22	African trypanosomiasis	4.7	8.7E-3	6
23	Homologous recombination	4.4	1.1E-2	6
24	Viral carcinogenesis	2.0	1.8E-2	15
25	Base excision repair	3.9	1.9E-2	6
26	Toll-like receptor signaling pathway	2.6	2.1E-2	9
27	Antigen processing and presentation	2.8	2.4E-2	8
28	Motor proteins	2.0	2.9E-2	13
29	IL-17 signaling pathway	2.6	3.4E-2	8
30	Tuberculosis	2.0	3.6E-2	12
31	Pyrimidine metabolism	3.2	3.7E-2	6
32	Alcoholism	1.9	3.9E-2	13
33	Virion - Flavivirus	9.1	4.1E-2	3
