# Supplementary material for: Identification of immune-associated genes with altered expression in the spleen of mice enriched with probiotic Lactobacillus species using RNA-seq profiling
Source: Anim Biosci. 2024 Aug 26;38(2):336–49. doi: 10.5713/ab.24.0280 (PMC11725755; doi:10.5713/ab.24.0280)
Supplement: Supplementary file 6 [file ab-24-0280-Supplementary-Table-5.pdf]

Align	Sublist	Category	Term	Fold Enrichment	P-Value	Count
108	3	MF	cytokine receptor activity	36.1	3.5E-8	7
109	4	MF	NAD+ nucleotidase, cyclic ADP-ribose generating	56.2	4.4E-5	4
110	5	MF	NAD(P)+ nucleosidase activity	56.2	4.4E-5	4
111	6	MF	signaling receptor activity	9.6	8.2E-5	7
112	7	MF	lipopeptide binding	126.5	2.2E-4	3
113	8	MF	cytokine binding	26.6	4.3E-4	4
114	9	MF	cytokine receptor binding	24.7	5.4E-4	4
115	10	MF	CXCR chemokine receptor binding	58.4	1.1E-3	3
116	11	MF	transmembrane signaling receptor activity	7.2	1.4E-3	6
117	12	MF	CCR chemokine receptor binding	24.5	6.5E-3	3
118	13	MF	interleukin-23 receptor activity	253.0	7.8E-3	2
119	14	MF	diacyl lipopeptide binding	253.0	7.8E-3	2
120	15	MF	interleukin-23 binding	253.0	7.8E-3	2
121	16	MF	interleukin-12 receptor binding	126.5	1.6E-2	2
122	17	MF	growth factor activity	6.7	2.2E-2	4
123	18	MF	lipoteichoic acid binding	72.3	2.7E-2	2
124	19	MF	double-stranded DNA binding	5.9	3.0E-2	4

Supplementary Table S5A

Sublist	Term	Fold Enrichment	P-Value	Count
1	Cell cycle	8.6	1.5E-28	44
2	Cytokine-cytokine receptor interaction	5.2	1.4E-22	51
3	Viral protein interaction with cytokine and cytokine receptor	8.0	2.7E-15	25
4	Hematopoietic cell lineage	5.8	8.8E-9	18
5	Chemokine signaling pathway	3.9	1.7E-8	25
6	DNA replication	9.5	1.2E-7	11
7	Oocyte meiosis	4.0	9.7E-6	16
8	Systemic lupus erythematosus	3.4	3.1E-5	17
9	Porphyrin metabolism	6.3	6.8E-5	9
10	Malaria	5.3	9.2E-5	10
11	Progesterone-mediated oocyte maturation	3.9	2.1E-4	12
12	p53 signaling pathway	4.1	6.2E-4	10
13	Inflammatory bowel disease	4.4	9.2E-4	9
14	Cellular senescence	2.7	9.3E-4	16
15	Rheumatoid arthritis	3.5	2.2E-3	10
16	Human T-cell leukemia virus 1 infection	2.2	3.3E-3	18
17	Virion - Human immunodeficiency virus	12.1	3.6E-3	4
18	Neutrophil extracellular trap formation	2.3	3.6E-3	16
19	JAK-STAT signaling pathway	2.5	4.3E-3	14
20	Epstein-Barr virus infection	2.1	8.5E-3	16
21	Asthma	6.0	8.5E-3	5
22	African trypanosomiasis	4.7	8.7E-3	6
23	Homologous recombination	4.4	1.1E-2	6
24	Viral carcinogenesis	2.0	1.8E-2	15
25	Base excision repair	3.9	1.9E-2	6
26	Toll-like receptor signaling pathway	2.6	2.1E-2	9
27	Antigen processing and presentation	2.8	2.4E-2	8
28	Motor proteins	2.0	2.9E-2	13
29	IL-17 signaling pathway	2.6	3.4E-2	8
30	Tuberculosis	2.0	3.6E-2	12
31	Pyrimidine metabolism	3.2	3.7E-2	6
32	Alcoholism	1.9	3.9E-2	13
33	Virion - Flavivirus	9.1	4.1E-2	3

Sublist	Term	Fold Enrichment	P-Value	Count
34	Glycerophospholipid metabolism	2.5	4.3E-2	8
35	Allograft rejection	3.0	4.7E-2	6
36	Graft-versus-host disease	3.0	4.7E-2	6

Supplementary Table S5B

Sublist	Term	Count	P-Value	Fold Enrichment
1	Cytokine-cytokine receptor interaction	38	4.1E-32	13.0
2	Viral protein interaction with cytokine and cytokine receptor	18	2.2E-17	19.1
3	Chemokine signaling pathway	15	4.5E-9	7.9
4	IL-17 signaling pathway	10	2.8E-7	10.8
5	JAK-STAT signaling pathway	12	8.2E-7	7.1
6	Rheumatoid arthritis	9	2.0E-6	10.4
7	Hematopoietic cell lineage	9	3.6E-6	9.6
8	Toll-like receptor signaling pathway	9	7.6E-6	8.7
9	Malaria	7	1.9E-5	12.4
10	Inflammatory bowel disease	7	3.0E-5	11.4
11	African trypanosomiasis	6	3.6E-5	15.5
12	Th17 cell differentiation	6	3.7E-3	5.8
13	Virion - Human immunodeficiency virus	3	4.1E-3	30.2
14	Kaposi sarcoma-associated herpesvirus infection	8	6.0E-3	3.6
15	Cytosolic DNA-sensing pathway	5	8.5E-3	6.1
16	Chagas disease	5	1.8E-2	4.9
17	NF-kappa B signaling pathway	5	2.0E-2	4.8
18	Amoebiasis	5	2.1E-2	4.7
19	C-type lectin receptor signaling pathway	5	2.4E-2	4.5
20	TNF signaling pathway	5	2.6E-2	4.4
21	Leishmaniasis	4	3.1E-2	5.8
22	Tuberculosis	6	3.2E-2	3.4
23	Antifolate resistance	3	3.3E-2	10.4
24	Osteoclast differentiation	5	4.4E-2	3.7

Supplementary Table S5C

Sublist	Term	Fold Enrichment	P-Value	Count
1	Cytokine-cytokine receptor interaction	17.7	3.3E-28	28
2	JAK-STAT signaling pathway	14.1	5.5E-11	13
3	Viral protein interaction with cytokine and cytokine receptor	19.5	1.3E-9	10
4	Inflammatory bowel disease	23.9	2.9E-8	8
5	Chemokine signaling pathway	8.7	6.4E-6	9
6	Hematopoietic cell lineage	13.8	9.1E-6	7
7	Toll-like receptor signaling pathway	12.5	1.6E-5	7
8	Amoebiasis	12.1	1.9E-5	7
9	Tuberculosis	8.2	4.0E-5	8
10	Legionellosis	15.5	2.7E-4	5
11	C-type lectin receptor signaling pathway	9.9	2.9E-4	6
12	Lipid and atherosclerosis	6.0	9.1E-4	7
13	Measles	7.7	9.6E-4	6
14	Virion - Human immunodeficiency virus	55.7	1.2E-3	3
15	Virion - Flavivirus	55.7	1.2E-3	3

Sublist	Term	Fold Enrichment	P-Value	Count
16	Phagosome	6.2	2.4E-3	6
17	Leishmaniasis	10.6	5.9E-3	4
18	Kaposi sarcoma-associated herpesvirus infection	5.0	6.0E-3	6
19	Asthma	22.3	7.6E-3	3
20	Rheumatoid arthritis	8.5	1.1E-2	4
21	Th1 and Th2 cell differentiation	8.4	1.1E-2	4
22	Chagas disease	7.2	1.7E-2	4
23	Th17 cell differentiation	7.1	1.8E-2	4
24	Intestinal immune network for IgA production	12.9	2.2E-2	3
25	Alcoholic liver disease	5.3	3.8E-2	4
26	Allograft rejection	9.3	4.0E-2	3
